# Unraveling Cecal Alterations in *Clostridioides difficile* Colonized Mice through Comprehensive Metabolic Profiling

**DOI:** 10.1021/acs.jproteome.4c00578

**Published:** 2024-10-31

**Authors:** Olga Deda, Emily G. Armitage, Thomai Mouskeftara, Melina Kachrimanidou, Ioannis Zervos, Andigoni Malousi, Neil J. Loftus, Ioannis Taitzoglou, Helen Gika

**Affiliations:** † Laboratory of Forensic Medicine & Toxicology, Department of Medicine, Aristotle University of Thessaloniki, 54124 Thessaloniki, Greece; ‡ Biomic AUTh, Center for Interdisciplinary Research and Innovation (CIRI-AUTH), Balkan Center B1.4, 10th km Thessaloniki-Thermi Road, GR 57001 Thessaloniki, Greece; § Shimadzu Corporation, Manchester M17 1GP, U.K.; ∥ 1^st^ Laboratory of Microbiology, Department of Medicine, Aristotle University of Thessaloniki, 54124 Thessaloniki, Greece; ⊥ Laboratory of Animal Physiology, Faculty of Veterinary Medicine, School of Health Sciences, Aristotle University of Thessaloniki, 54124 Thessaloniki, Greece; # Laboratory of Biological Chemistry, Department of Medicine, Aristotle University of Thessaloniki, 54124 Thessaloniki, Greece; ∇ Laboratory of Development-Breeding of Animal Models and Biochemical Research, School of Health Sciences, Aristotle University of Thessaloniki, 54124 Thessaloniki, Greece

**Keywords:** Gut−brain axis, *Clostridioides difficile* infection (CDI), Cecal metabolome, Antibiotic administration, Metabolomics, Fecal microbiota transplantation (FMT), Biochemical pathways, Metabolites, Metabolic profiling, Mice

## Abstract

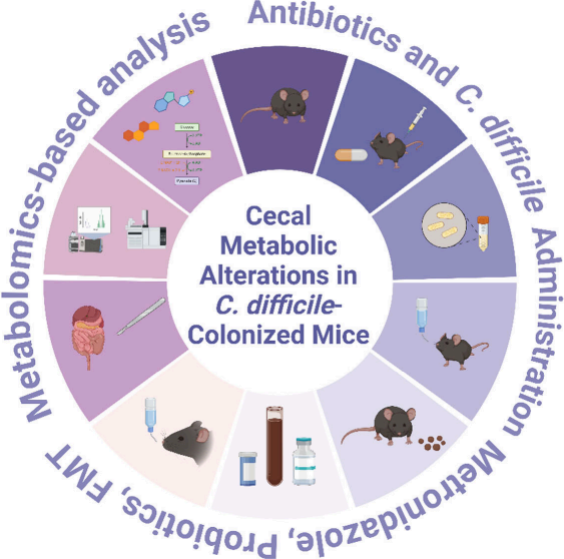

The disruption of gut microbiota caused by antibiotics favors the intestinal colonization of *Clostridioides difficile* – a Gram-positive, spore-forming anaerobic bacterium that causes potentially fatal gastrointestinal infections. In an endeavor to elucidate the complexities of the gut-brain axis in the context of *Clostridium difficile* infection (CDI), a murine model has been used to investigate the potential effects of antibiotic administration and subsequent colonization by *C. difficile*, as well as the impact of three different 10-day treatments (metronidazole, probiotics, and fecal microbiota transplantation), on the cecal metabolome for the first time. This follows our previous research which highlighted the metabolic effect of CDI and these treatments in the brain and employs the same four different metabolomics-based methods (targeted GC-MS/MS, targeted HILIC-MS/MS, untargeted RP-LC-HRMS/MS and untargeted GC-MS). A total of 286 unique metabolites have been identified in the mouse cecal profiles and statistical analysis revealed that CDI, as well as the subsequent treatments, significantly alters cecal metabolites and lipids implicated in various biochemical pathways centered around amino acid metabolism, glycerophospholipid metabolism, and central carbon metabolism. To our knowledge, this study represents the first exploration of the effects of *C. difficile*-induced colitis and potential treatments on the cecal tissue metabolome.

## 1 Introduction

*Clostridioides difficile (C. difficile*), a Gram-positive, spore-forming anaerobic bacterium, causes a spectrum of gastrointestinal infections that can be mild, severe, or even fatal and can affect individuals of all ages.^1^ While it is present in the microbiome of healthy infants and adults (approximately 53%), this percentage increases in the elderly and immunocompromised individuals. Antibiotic use disturbs the ecological balance of the gut microbiome by providing a barrier against pathogens which leads to favorable conditions for *C. difficile* colonization. In healthcare settings, *C. difficile* presents a notable nosocomial threat, contributing substantially to an estimated annual treatment cost of $5 billion in the USA^2−4^. Effective prevention and treatment strategies are imperative, necessitating a nuanced understanding of the intricate dynamics between the microbiome, antibiotic exposure, and individual susceptibility to *C. difficile* infections. Addressing this issue is critical, given its impact on public health and the considerable economic burden it imposes.

The application of metabolomics in investigating *C. difficile*-induced colitis has the potential to provide a comprehensive understanding of the altered biochemical pathways in response to infection. Metabolomics studies facilitate the early detection, diagnosis, and monitoring of treatment efficacy. Given that many factors influence the metabolome, the metabolomics-based approach could suggest more tailored and personalized treatments and improved outcomes for patients.

In a recent study, cecal samples from a mouse model were extensively analyzed to investigate the impact of different antibiotic treatments on susceptibility to *Clostridium difficile* infection (CDI) and its persistence. Metabolomics was employed as part of a multiomics approach revealing that the choice of antibiotic treatment regimen had a pronounced effect on CDI outcomes.^5^ CDI induced notable metabolic changes, significantly pronounced in mice pretreated with cefoperazone and streptomycin. Furthermore, CDI was associated with elevated levels of Stickland fermentation substrates, such as proline conjugates, arabonate/xylonate, and 5-aminovalerate, as well as a substantial reduction in secondary bile acids. Clindamycin pretreatment had a unique impact by reducing both the inputs and outputs of Stickland fermentation compared to untreated mice, highlighting the antibiotic’s specific influence on this metabolic pathway.

In another study, LC/MS-based untargeted metabolomics was used to explore the effects of cefoperazone treatment on the cecal microbiota of wild-type (WT) and major histocompatibility complex class I-related molecule MR1^–/–^ knockout (KO) mice.^6^ The results indicated that KO mice experienced less disruption in their cecal microbiota in response to the treatment, while WT mice exhibited an increase in carbohydrates.

Antibiotic treatment can reduce resistance to *C. difficile* colonization, resulting in an altered gut microbiome that further promotes C. difficile growth through higher nutrient availability. In a recent study using cecal samples,^7^ the importance of amino acids and other nutrients in the early stages *of C. difficile* colonization and disease progression was highlighted. Researchers utilized a murine model, employing mass spectrometry and RNA sequencing to monitor perturbations for up to 30 h after acute CDI infection. Amino acids, particularly proline and branched-chain amino acids, as well as carbohydrates, decreased over time in the mouse cecum samples, aligning with *C. difficile* gene expression patterns.

*C. difficile* is known to influence host tryptophan metabolism. A recent study^8^ focused on this pathway demonstrated that when tryptophan catabolism was inhibited in indoleamine 2,3-dioxygenase (IDO1) knockout mice, it resulted in increased mucosal damage, cecal bleeding, and elevated IFN-γ production during CDI. The study concluded that *C. difficile* induces tryptophan catabolism as a self-regulatory mechanism, which limits immunopathology and the accumulation of IFN-γ-expressing neutrophils during infection.

In a separate study, cecal metabolomics analysis revealed that the supplementation of high doses of *Bifidobacterium breve YH68*, either alone or in conjunction with vancomycin and metronidazole, significantly outperformed the combination of these two antibiotics in treating primary CDI in mice.^9^ This approach led to the reduction of *C. difficile* levels, decreased toxin production, and improved the survival rates of the mice. It also positively impacted cecal microbiota diversity by enhancing beneficial bacteria such as Bifidobacterium, hence increasing crucial metabolites such as secondary bile and butyric acids. These results highlight the potential of probiotics in the therapy for CDI, as evidenced through cecal metabolomics and *C. difficile* analysis.

In our earlier investigation,^10^ we examined the effects of three distinct 10-day treatments—metronidazole, probiotics, and fecal microbiota transplantation—on brain samples from C57BL/6 mice colonized by a nontoxigenic strain of *C. difficile* after antibiotic administration. These treatments were designed based on the concept of gut-brain interaction. Metabolic profiles from mice administered one of these treatments were compared to those from uninfected mice, as well as from CDI-infected but untreated mice, using four metabolomics-based methods, including both targeted and untargeted approaches.

Dysbiosis resulting from antibiotic administration and the colonization of *C. difficile* spores affected small molecules acting as second messengers and metabolic suppliers and precursors. The selected treatments included widely consumed probiotics and the commonly used clinical first-choice antibiotic, metronidazole (Flagyl), along with the promising approach of fecal microbiota transplantation (FMT). Over 200 metabolites were identified in the brain extracts including glycerophospholipids and other glycerolipids, amino acids, carbohydrates, and free fatty acids. The impact of the therapeutic interventions against CDI led to significant and substantial alterations in the brain metabolome of antibiotic-treated mice colonized with *C. difficile* compared to the controls. Notably, none of the 10-day treatments fully restored the altered gut microbiome ecology.

Building on this research and using the same murine model, we have now focused on the cecal metabolome. This was prompted by evidence demonstrating that while *C. difficile* can proliferate throughout various parts of the mouse intestinal tract, the production of toxins specifically occurs within the cecum and colon.^11^ Furthermore, the cecum is also important in microbial fermentation and exhibits high microbial diversity, like the colon and rectum. Its microbial community structure closely resembles that of the lower gastrointestinal tract and shows similarities across different rodent species, making fecal samples a useful proxy for studying the microbiota of the cecum.^12^

In this context, we present results derived from the application of four metabolomic-based methods: targeted Gas Chromatography coupled with Tandem Mass Spectrometry (GC-MS/MS), targeted Hydrophilic Interaction Liquid Chromatography coupled with Tandem Mass Spectrometry (HILIC-MS/MS), untargeted Reversed-Phase Liquid Chromatography coupled with High-Resolution Mass Spectrometry (RP-LC-HRMS/MS) and untargeted Gas Chromatography coupled with Mass Spectrometry (GC-MS). The utilization of these analytical methods provides a more comprehensive understanding of the systemic impact on different sample types within the same experimental framework. We examined cecal tissue because the cecum is a primary site of bacterial metabolism, enabling us to evaluate functional differences within the microbiota and its associated metabolites through the symbiotic relationship. To our knowledge, this study represents the first exploration of the effects of *C. difficile*-induced colitis and potential treatments, applying advanced analytical platforms directly on cecum tissue extracts.

## 2 Materials and Methods

### 2.1 Reagents and Materials

Methanol (MeOH), *N*-Methyl-N-(trimethylsilyl) trifluoroacetamide (MSTFA), trimethylchlorosilane (TMCS), Methoxyamine hydrochloride (MeOX) and pyridine anhydrous were purchased from Sigma-Aldrich (Merck, Darmstadt, Germany). Internal standards, myristic acid-d27 for GC-MS untargeted analysis, 4-phenylbutyric for organic acids analysis and injection standard N-pentadecane were also obtained from Sigma-Aldrich (Merck, Darmstadt, Germany). Methyl-*tert*-butyl-ester (MTBE), acetonitrile (ACN), and ammonium formate were obtained from CHEM-LAB NV (Zedelgem, Belgium). Deionized water (ddH_2_O) was ultrapurified using a Millipore instrument (Bedford, MA, USA) delivering water quality of a resistivity ≥18.2 MΩ•cm. LC-MS grade acetonitrile and water used in the RP-LC-HRMS/MS analysis was purchased from Romil Ltd., Cambridge, UK. Metabolite standards and formic acid used in the RP-LC-HRMS/MS analysis were purchased from Sigma-Aldrich (Gillingham, UK). Antibiotics, including metronidazole, vancomycin, kanamycin, gentamycin, colistin, and clindamycin, were purchased from Sigma–Aldrich (St. Louis, MO, USA).

### 2.2 *Clostridioides difficile* Spore Purification

A nontoxigenic strain of *C. difficile*, lacking tcdA, tcdB, cdtA, and cdtB genes,^13^ was cultured on Columbia blood agar at 37 °C for 48 h.^14^ Subsequently, the culture was inoculated into 40 mL autoclaved Clospore liquid medium^15^ and was anaerobically incubated at 37 °C for 16 days.^16^ Spore harvesting was carried out with three 20 min centrifugation cycles at 10,000 × g, followed by washing and storage at 4 °C. Before the *in vivo* experiment, spores underwent heat treatment at 65 °C for 20 min to eliminate vegetative cells. The spores were then diluted to achieve a density of 2,000 spores/20 μL and verified through microscopic examination^17^ and viable spore enumeration on taurocholate, cefoxitin, cycloserine, and fructose (TCCFA) agar.^14^

Colonization of *C. difficile* was confirmed by culturing homogenized mouse fecal samples on TCCFA plates after ethanol treatment. All procedures were conducted under biosafety level II conditions. The detailed methodology can be found in a previously published article.^10^

### 2.3 Animal Experiment

The animal study was conducted at the Laboratory of Development–Breeding of Animal Models and Biomedical Research, School of Health Science, Aristotle University of Thessaloniki, Greece. The protocol adhered to the 2010/63/EU Directive, as well as the Presidential Decree No. 56/2013 of Greek legislation, and was approved by the Department of Rural Economy and Veterinary Medicine, Prefecture of Central Macedonia, Hellenic Republic [Protocol number: 634754(2485)].

The detailed experimental design is outlined in a previously published article on the same concept concerning brain samples.^10^ In brief, 50 C57BL/6 male mice were bred in regulated dark cycles and controlled temperature and humidity conditions. The number of animals used in this project adhered to the 3Rs principle, and statistical significance was ensured based on the power analysis performed using MetaboAnalyst 6.0.^18^ Following a one-week acclimatization period, the 12-year-old animals were divided into five groups (ten animals per group) (Table 1). Groups G1 to G4 were exposed to an antibiotic cocktail in their drinking water for 4 days. On the sixth day, a single dose of clindamycin (10 mg/kg) was injected intraperitoneally.^19,20^ The next day, these animals were challenged with nontoxigenic *C. difficile via* drinking water,^16^ while G5 (control group) remained untreated.

**Table 1:** Experimental Groups with Their Respective Conditions and Treatments^a^

Experimental Group	Antibiotic Cocktail and Clindamycin Administration	*C. difficile* Infection	Treatment
G1	+	+	Metronidazole
G2	+	+	Probiotics
G3	+	+	FMT
G4	+	+	-
G5	_	-	-

a Groups G1 to G4 received an antibiotic cocktail and clindamycin followed by *C. difficile* infection, with different treatments applied to each group. Group G5 served as the control group and did not receive any antibiotic cocktail, clindamycin, or *C. difficile* infection. Treatments included metronidazole, probiotics, and fecal microbiota transplantation (FMT).

Three days postinfection, groups G1, G2, and G3 underwent distinct 10-day treatments via daily drinking water. Group G1 received metronidazole (50 mg/kg/day),^21^ group G2 a probiotic product (a commercially available probiotic product containing 5 × 10^9^ viable strains: *L. casei, B. lactis, B. longum, B. bifidum, L. salivarius, L. bulgaricus, L. plantarum, L. rhamnosus, L. acidophilus, S. thermophiles, Lactococcus lactis*), while group G3 received Fecal Microbiota Transplantation (FMT).^22^ Group G4 remained untreated. Tissues were collected post-mortem, washed, frozen, and stored at −80 °C. Procedures were conducted under a laminar flow hood with appropriate protective equipment.

### 2.4 Metabolite Extraction for Multiple Analyses

Cecum tissue samples were thawed at room temperature for 1 h and homogenized using a Bead mill Homogenizer (BEAD RUPTOR ELITE, Omni International, Kennesaw, Georgia). To enable sample analysis for each analytical approach, two successive extractions were performed for extraction of both hydrophilic and lipophilic metabolites utilizing a two-step procedure. Tissues were weighed (250 mg ±16) and transferred to 1.5 mL tubes containing 1.0 mm ceramic beads. To extract the hydrophilic compounds, the organic solvent mixture of MeOH/IPA/H_2_O 1:1:2 (v/v/v) was added in a ratio of tissue weight/solvent volume of 1:3 (w_ceacum_/v_solvent_). The samples were vortexed, sonicated, homogenized (3 cycles of 30 s, at a speed of 6.00 m/s) and the homogenates were centrifuged for 20 min at 11.180*g*. The supernatants were collected and divided into 2 aliquots; 200 μL were retrieved for HILIC-MS/MS analysis and 100 μL for GC-MS analysis. For the extraction of lipophilic metabolites, a solvent mixture of MTBE/MeOH 3:1 (v/v) was added to the dry pellet in a proportion of tissue weight/solvent volume 1:3 (w_cecum_/v_solvent_). Subsequently, the samples were vortexed for 20 min, then centrifuged (30 min at 11.180*g*), and 600 μL of the supernatants were transferred to 1.5 mL Eppendorf tubes. All aliquots were evaporated to dryness *under vacuo* (SpeedVac, Eppendorf Austria GmbH, Wien, Austria) and the dried extracts were stored at −80 °C until analyzed.

### 2.5 Sample Analysis

Cecum tissue extracts were analyzed by four different methods: untargeted GC-MS, targeted GC-MS/MS, targeted HILIC-MS/MS, and RP-LC-HRMS/MS as described previously by Deda et al.^10^

In all cases, samples were analyzed in a randomized order after ten equilibration injections. For GC-MS analyses, QC samples were prepared by mixing equal volumes of all samples after derivatization reactions and were analyzed every ten samples within the batch.

Briefly, for GC-MS global profiling and GC-MS/MS organic acids analysis, 10 μL of myristic acid-d27 (IS, 100 μg/mL) and 10 μL 4-phenylbutyric acid (IS, 100 μg/mL) were added to each extract before the evaporation. Sixty-five (65) μL of 2% MeOX in anhydrous pyridine were added to the dried extracts. The samples were incubated at 70 °C for 2 h and left to cool down at room temperature for 10 min. After the end of the first derivatization, 125 μL of MSTFA 1% TMCS were added, followed by incubation for 1 h at 70 °C, aiming to the formation of TMS derivatives. By the end of the derivatizations, 10 μL of the injection standard (N-pentadecane, 100 μg/mL) were added and the samples were split into 2 vials for the analysis in targeted and untargeted mode. The detailed conditions for Bruker GC-MS for both analyses are provided in our previous publications.^23,10^

For HILIC-MS/MS analysis, the dried extracts were reconstituted in 70 μL ACN/H_2_O, 95:5 (v/v), and injected onto an ACQUITY UPLC H-Class system coupled with a Xevo TQD mass spectrometer operating in MRM mode in both positive and negative ionization modes. Metabolite separation was performed on an ACQUITY UPLC BEH Amide column (Waters Ltd., Elstree, UK) with a flow rate of 0.5 mL/min and a temperature of 40 °C, using a binary solvent system consisting from solvent A: ACN/H_2_O 95:5 (v/v) and B ACN/H_2_O 30:70 (v/v), both containing 10 mM ammonium formate buffer at pH 6.^24^

Concerning RP-LC-HRMS/MS, dried hydrophilic and lipophilic extracts were reconstituted in 150 μL methanol each, then vortex-mixed and shaken vigorously for 45 min. The two extracts were subsequently combined, then samples were centrifuged at 16,000 × g for 20 min and supernatants transferred to LC-MS vials. QC samples were prepared by mixing equal volumes of all samples, which were analyzed at the start of the batch (10 injections) and throughout the batch after every five samples analyzed in random order. The samples were analyzed in positive and negative ESI modes using data-independent acquisition (DIA)-MS/MS based on previously published methods.^10,25^ The RP-LC-HRMS/MS system was a Nexera X2 LC coupled to an LCMS-9030 Q-TOF (Shimadzu Corporation, Kyoto, Japan). Chromatographic separations were performed using an ACQUITY UPLC BEH C18 column (1.7 μm, 2.1 × 100 mm, Waters Ltd., Elstree, UK) with a 35 min binary gradient of Solvent A (water with 0.1% formic acid) and Solvent B (acetonitrile with 0.1% formic acid). The method acquired a single TOF MS scan (*m*/*z* 65–1,000) followed by 27 DIA-MS/MS mass scans over a mass range of *m*/*z* 40–1,000. Each DIA-MS/MS mass scan had a precursor isolation width of 35 Da and a collision energy spread of 5–55 V, resulting in a cycle time of <1 s. This allowed the collection of fragmentation data for all masses in the spectra across the entire LC gradient.

### 2.6 Data Analysis

Data from GC-MS/MS organic acids analysis were processed using Bruker MSWS8 software, and peak areas were obtained. For HILIC-MS/MS data analysis was performed by TargetLynx (v4.1) (Waters, Milford, MA, USA), and peak areas were considered. RP-LC-HRMS/MS data were processed using LabSolutions Insight 3.8 software and MS peak areas were considered for statistical analysis. An in-house built MS/MS library of metabolites and lipids commonly detected with this analytical method was used to identify metabolites. Library MS/MS spectra were acquired from authentic reference material when available. Raw data from untargeted GC-MS analysis was converted to CDF file format through NetCDF, and imported to AMDIS for peak deconvolution and identification of metabolites using the NIST17 Mass Spectral Library (mainlib library).^26^ AMDIS files and CDF files were further processed using the Gavin3 script in Matlab software for peak alignment and integration.

Prior to statistical analysis, untargeted GC-MS and RP-LC-HRMS/MS data were evaluated concerning the quality of the obtained results. Only metabolites that were present in 80% of the analyzed samples were considered. Τhe injection standard, N-pentadecane (100 μg/mL), was used to normalize the obtained data from untargeted GC-MS analysis, resulting in lower CV% in the quality control samples for each compound. Normalization was performed by dividing the area of each compound by the area of the injection standard, and this value was used for further statistical analysis. For organic acid analysis using GC-MS/MS, 4-phenylbutyric acid (IS, 100 μg/mL) was used as the internal standard, and the obtained data were normalized as mentioned earlier. The data were also assessed through clustering of quality control (QC samples) in principal components analysis (PCA) and only the compounds with a value of coefficient of variation (CV) of less than 30% in the QC samples were included in further processing.

Regarding multivariate statistical analysis, SIMCA 13.0.3 (UMETRICS AB, Umea, Sweden),^27^ was used and biomarker evaluation *via* Variable Importance for the Projection plots (VIP), p(corr), loading plots and S-plots was performed. Principal Components Analysis (PCA) and Orthogonal Projection to Latent Structures Discriminant Analysis (OPLS-DA) models were generated to compare metabolic profiles from each of the treatment groups compared to controls. Model validation parameters were evaluated (R2X: fraction of the variation in X explained by the model, R2Y: total sum of variation in Y explained by the model and Q2Y: goodness of prediction), permutation plots were constructed, and CV-ANOVA p-values were calculated to enhance the reliability of the models. Univariate analysis, including p-values from two-tailed Student’s *t* tests and Log2 fold change calculations, was performed using Microsoft Excel 365. MetaboAnalyst 6.0 was used for pathway analysis and interpretation of the metabolic procedures.^18^ R version 4.3.2 was used to build the plots of the pathway analysis results.

## 3 Results

### 3.1 GC-MS/MS Analysis

In the analysis of mouse cecal samples from five distinct groups (G1 metronidazole treatment, G2 probiotics treatment, G3 FMT treatment, G4 infected but untreated controls G5 uninfected and untreated controls), 33 organic acids were identified and 20 remained undetected using the validated targeted GC-MS method (Table S1.).^23^

Upon investigating the impact of *C. difficile* infection by comparing groups G4 and G5, seven organic acids exhibited statistically significant differences between the two groups, as determined by univariate statistics: 3-hydroxybutyric, 4-hydroxybenzoic, citric, ethylmalonic, hippuric, lactic, and malonic acids. Notably, 3-hydroxybutyric and lactic acids displayed the most pronounced differentiation. Furthermore, both hippuric acid and malonic acid demonstrated a substantial log 2FC difference, which was greater than 2 times. In the infected groups, malonic acid levels increased, while hippuric acid levels decreased. In the evaluation of multivariate analysis (Table S2.), four organic acids had VIP values ≥1.5: lactic, 3-hydroxybutyric, citric, and malonic acids, with VIP values of 2.0, 1.8, 1.5, and 1.5, respectively.

In the comparative analysis between *C. difficile*-infected but untreated mice and those undergoing treatment (G4-G1, G4-G2, G4-G3), the discerning factor among the groups emerged through the identification of organic acids. Metronidazole treatment had a significant impact on 15 acids, particularly 4-aminobutyric acid, which was diminished by metronidazole treatment and exhibited the most pronounced differentiation with *p* ≤ 0.01 and a |log2FC| exceeding 1.5. 2-hydroxyisovaleric, 4-hydroxyphenylacetic, adipic acid and hippuric acid all demonstrated statistical significance and a |log2FC| greater than 1.5, while glyceric acid also exhibited a VIP value ≥1.5.

Based on organic acids, probiotic treatment was the second most effective; it influenced 10 significant metabolic alterations, including 4-aminobutyric, 5-hydroxyindole-3-acetic, ethylmalonic, fumaric, glyceric, lactic, malic, methylmalonic, pyroglutamic, and vanillylmandelic acids. Once again, 4-aminobutyric acid met all three criteria of p-value ≤0.01, |Log2 FC| ≥ 1.5, and VIP ≥ 1.5, while lactic acid fulfilled two criteria of p-value ≤0.01 and VIP ≥ 1.5.

Fecal Microbiota Transplantation (FMT) demonstrated a slightly lower impact with 9 differentiated compounds, including 2-Hydroxybutyric, 4-aminobutyric, azelaic, ethylmalonic, lactic, methylmalonic, pyroglutamic, succinic, and vanillylmandelic acids. Methylmalonic acid also exhibited a VIP value ≥1.5.

**Figure 1:**
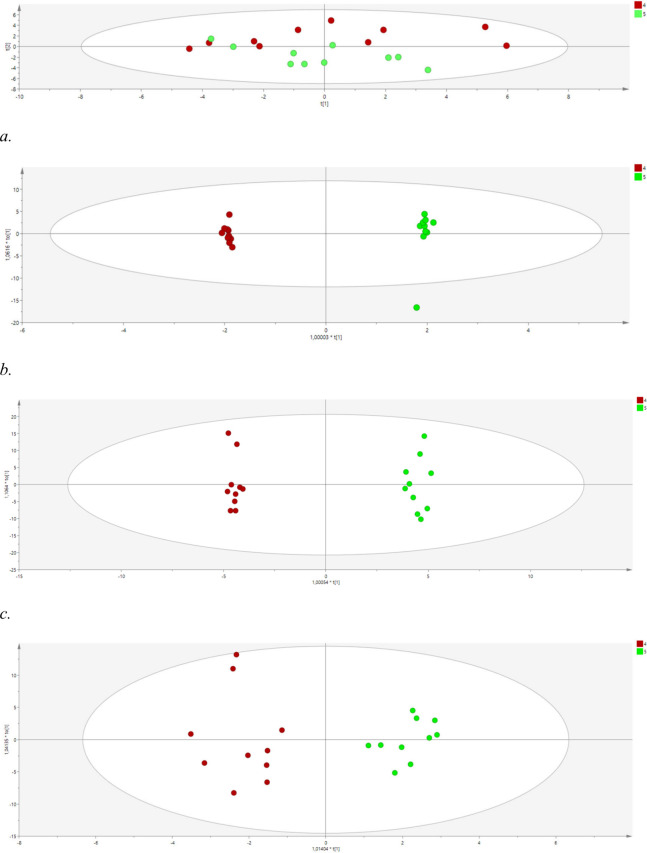
a. PCA score plot of the constructed model from the targeted GC-MS/MS analysis shows Group 4 (C. difficile untreated group, in red) and Group 5 (control, in green), demonstrating a clear cluster along the t[2] principal component, 1b. OPLS-DA score plot of the constructed model from the targeted HILC-MS/MS analysis of Group 4 (C. difficile untreated group, in red) and Group 5 (control, in green) with a distinct cluster and CV-ANOVA p value of 2.78399 × 10^–04^, 1c. OPLS-DA score plot of the constructed model from untargeted of Group 4 (C. difficile untreated group, in red) and Group 5 (control, in green) with a CV-ANOVA value of 2.09597 × 10^–03^, 1d. OPLS-DA score plot of the constructed model from the untargeted GC-MS of Group 4 (C. difficile untreated group, in red) and Group 5 (control, in green) with a CV-ANOVA value of 7.92885 × 10^–03^.

PCA revealed a separation between the *C. difficile*-infected group (G4) and the controls (Figure 1a.). OPLS-DA of each treated group (G1, G2, G3) vs the infected untreated group (G4) was used to investigate the metabolic effect of each treatment (Figure S1.).

In the investigation into whether infected mice would revert post-treatment to baseline metabolic levels observed in controls, focusing on a targeted profile of 33 organic acids, none of the treatments led to a complete restoration. Even after a 10-day treatment, the cecal metabolome did not fully recover. The Metronidazole group exhibited differentiation from the control group in 16 out of 33 metabolites, while the Probiotics group showed significant alterations in 15 out of 33, and the FMT group demonstrated differences in only 9. Particularly notable compounds for the Metronidazole group included 4-aminobutyric, 4-hydroxyphenylacetic, adipic, and citric acid. In the Probiotics group, 4-aminobutyric acid met the criteria with a p-value >0.01 and |log2FC| ≥ 1.5. Remarkably, the OPLS-DA models for the comparison between G2 and G3 versus G5 did not meet the set criterion of a CV-ANOVA value greater than or equal to 0.05.

Summarizing the extensive assessment of the 33 organic acids and their influence on the investigated cecal metabolome in the mouse model, a key observation is the central role of 4-aminobutyric acid. It demonstrated alterations in 8 out of 10 total comparisons across the five groups. Additionally, lactic acid showed significant changes in 7 comparisons, while 4-hydroxybenzoic, ethylmalonic, glyceric, methylmalonic, and vanillylmandelic acids displayed statistical differentiation in 6 out of 10 comparisons.

### 3.2 HILIC-MS/MS Analysis

Cecal samples from mice were subjected to analysis using the HILIC-MS/MS method^24^ specifically developed for small polar metabolites. This method, previously employed in the analysis of mouse brain samples from the same animal model,^10^ successfully detected 42 metabolites in the mouse cecum tissue extracts (Table S3.). Once again, QC samples were incorporated to assess the analytical performance and the stability of the analytical system. Additionally, peak area relative standard deviation percentage (RSD%) of the QC samples was calculated to evaluate the precision and reproducibility of analytical measurements, ensuring the stability and consistency of the method over time.

In the context of investigating the impact of *C. difficile* infection (G4-G5), 8 compounds were identified as statistically significant differentiators. These included creatinine, cytosine, hypotaurine, methylamine, monoisamylamine, thymidine, thymine, and trimethylamine-N-oxide. Notably, hypotaurine exhibited a |Log2 FC| ≥ 1.5, indicating reduced levels during infection. Hypotaurine, monoisamylamine, and trimethylamine-N-oxide further displayed VIP values of 1.9, 1.7, and 1.5, respectively, based on multivariate statistical analysis.

The PCA scores plot revealed a distinct clustering trend between the infected (G4) group and the controls (G5), a pattern that was more pronounced and reinforced in the OPLS-DA scores plot, in the first two components (Figure 1b). The cross-validated analysis of variance (CV-ANOVA) value was determined to be 2.78399 × 10^–04^, affirming the robustness of the model.

In assessing the impact of three treatments - metronidazole, probiotics, and FMT - on small polar metabolites from cecal mouse samples, distinct effects were observed. Probiotics emerged as the most influential, significantly altering 13 metabolites, followed by metronidazole, which impacted 10 metabolites. A comparison between the FMT-treated group (G3) and the untreated group (G4) revealed only 6 differentiating compounds, indicating a closer alignment of G3 with the infected mouse metabolome. Metronidazole induced notable changes in adenine, adenosine, creatinine, histamine, inosine, leucine, thiamine, thymidine, trimethylamine-n-oxide, tryptophan, and adenosine; the effect on adenosine was also significant based on multivariate statistics. For probiotics, again, adenosine, histamine, inosine, and thiamine exhibited significance. Additionally, creatine, cytosine, monoisamylamine, riboflavin, taurine, uridine, and trimethylamine were also significant, while hypotaurine displayed a high |log2FC| value. Taurine, monoisamylamine, and histamine, with VIP values of 1.6, 1.5, and 1.5, respectively, played crucial roles in discriminating between treatment groups, based on multivariate statistics. Hypotaurine stood out as profoundly significant when comparing G3 and G4, alongside betaine, creatine, creatinine, guanine, and hypoxanthine, emphasizing their relevance in distinguishing metabolic profiles. For the last comparison, the OPLS-DA constructed model did not pass the criterion of CV-ANOVA values of ≤0.05, and thus, no VIP values for this comparison are considered (Table S4).

When examining the post-treatment metabolome, it became evident that the administration of metronidazole induced variations in 9 compounds compared to uninfected mice. Additionally, the two other treatments displayed differences in 4 compounds each, suggesting a metabolic shift toward a profile more akin to the uninfected control group. Focusing solely on the metabolites obtained through the HILIC-MS/MS method, this observation hints at a potential reversion to a more baseline metabolic state.

The metronidazole-treated group exhibited alterations in the levels of specific compounds, including adenine, adenosine, anthranilic acid, histamine, hypotaurine, isoleucine, leucine, monoisoamylamine, and phenylalanine. Particularly noteworthy were the significant |log2FC| values observed for adenosine and monoisoamylamine, as per the predetermined significance criterion. Conversely, in the probiotics-treated group, variations were detected in four compounds—methylamine, putrescine, trimethylamine-N-oxide, and uridine—when compared to the controls. Distinctive changes in anthranilic acid, cotinine, monoisoamylamine, and uridine were observed in the FMT-treated mice relative to the control group. Multivariate analysis underscored the significance of histamine, adenosine, and monoisoamylamine for G1 versus G5, meeting the criterion of a VIP value ≥1.5. Although cytosine and taurine did not attain statistical significance in univariate analyses, they demonstrated significance in the constructed OPLS-DA model between G2 and G5, as evidenced by the VIP criterion. Moreover, when comparing G3 to G5, monoisoamylamine, anthranilic acid, and uridine exhibited VIP values of 1.7, 1.5, and 1.5, respectively. These findings highlight the critical importance of adopting a comprehensive and integrated analytical approach that combines both univariate and multivariate methodologies.

In summary, the targeted HILIC-MS/MS method identified 42 cecal compounds in the investigated murine model. Notably, monoisoamylamine exhibited alterations in 6 out of 10 comparisons, while hypotaurine showed changes in half of the comparisons. Additionally, adenine, adenosine, creatine, cytosine, histamine, and leucine were consistently altered in 4 out of 10 comparisons. These findings emphasize the potential significance of these compounds within the metabolic landscape of the studied murine model.

### 3.3 RP-LC-HRMS/MS Analysis

Untargeted RP-LC-HRMS/MS analysis using data independent acquisition was conducted in line with previous research into the effect of microbiome disruption by *C. difficile* colonization in brain.^10^ A total of 204 metabolites could be identified by mass, retention time and MS/MS spectral correspondence to an in-house built MS/MS library of metabolites and lipids commonly detected with this analytical method including MS/MS spectra from authentic reference material when available (Table S5.). Consistent with each methodology employed for sample analysis, an evaluation of the analytical system’s stability was undertaken. Peak area RSD% for these compounds were all <30, with 90% of the compounds having RSD% < 10. Among the identified compounds, acetylcarnitine, adenine, adenosine, alanine, betaine, choline, creatine, creatinine, cytosine, hypoxanthine, inosine, isoleucine, leucine, methionine, nicotinamide, nicotinic acid, pantothenate, phenylalanine, proline, riboflavin, taurine, thymine, tryptophan, tyrosine, uridine, and xanthine were also detected using the targeted HILIC-MS/MS method. Additionally, malic acid and succinic acid were identified using the targeted GC-MS method for the organic compounds. To maintain the focus on the targeted detection of specific compounds, these compounds were excluded from the RP-LC-MS/MS data set, leaving 176 unique compounds for statistical analysis.

In the investigation of the impact of *C. difficile* infection (G4-G5), 27 compounds exhibited statistically significant alterations with p values ≤0.05. Among these, 16 compounds demonstrated higher significance with p values ≤0.01, including 5′-methylthioadenosine, asparagine, cholesterol, cytidine, deoxycholic acid, dimethylglycine, glutamine, glutathione disulfide, LPC 15:0 sn-1, LPC 15:0 sn-2, LPC 17:0 sn-1, LPC 17:0 sn-2, LPC 22:6 sn-1, LPE 17:0 sn-1, LPE 17:0 sn-2, LPG 18:0 sn-1, LPG 18:0 sn-2, LPG 22:6 sn-2, LPI 16:0 sn-2, LPI 18:0 sn-2, N-arachidonoyl taurine, N-linoleoyl taurine, ornithine, orotidine, phenylacetylglycine, taurocholic acid, and ursodeoxycholic acid. Orotidine, and ursodeoxycholic acid exhibited |log2FC| greater than 1.5. Multivariate statistical analysis revealed that asparagine, N-linoleoyl taurine, N-arachidonoyl taurine, LPC 17:0 sn-2, LPE 17:0 sn-2,, glutathione disulfide,, LPG 22:6 sn-2, dimethylglycine, deoxycholic acid, phenylacetylglycine, ursodeoxycholic acid, LPC 15:0 sn-1, LPC 17:0 sn-1, cytidine, orotidine, and LPE 17:0 sn-1 were responsible for group differentiation, as indicated by descending VIP values ranging from 2 to 1.5.

When comparing untreated *C. difficile*-infected mice to those undergoing different treatments (G4-G1, G4-G2, G4-G3) after infection, it was observed that metronidazole exhibited the most pronounced effect, impacting 20 metabolites. Probiotics closely followed with 19 altered metabolites, and FMT showed a notable impact on 17 compounds, as presented in Table S5. Remarkably, among the differentiated metabolites, 13 were altered in two out of the three treatments, while five compounds—guanosine, LPE 16:1 sn-2, phenylacetylglycine, spermidine, and uracil—were altered by all three treatments, with guanosine and uracil showing the highest scores, and phenylacetylglycine exhibiting a |log2FC| according to the set criterion. It is noteworthy that none of the three constructed discriminant OPLS-DA models showed significance in the CV-ANOVA (Table S6). Consequently, VIP values were not computed.

In the investigation into whether infected mice would revert post-treatment to the baseline metabolic levels observed in controls, none of the treatments had such an effect. Despite the 10-day treatment, the cecal metabolome did not fully recover. Notably, the metronidazole group exhibited the most pronounced differentiation from the control group, with an alteration of 54 metabolites observed in both targeted methods. This highlights the substantial impact of the used antibiotics on the cecal metabolome. Probiotics followed with 41 compounds, and FMT demonstrated a closer proximity to controls with 37 altered compounds, albeit still distinct.

Multivariate statistics provided strong, statistically significant models for these comparisons (Figure S2.). In the metronidazole group, 5 compounds—LPG 22:6 sn-2 cholesterol, deoxycholic acid, orotidine, dimethylglycine—were identified with VIP values meeting the set criterion. For probiotics, compounds such as glucose 6-phosphate, dimethylglycine, orotidine, cytidine, ursodeoxycholic acid, n2-acetyllysine, glutamic acid, LPC 17:0 sn-2, LPE 16:1 sn-2, LPE 18:1 sn-2 contributed to the differentiation, each with descending VIP values. In the group 3 versus group 5 comparison, multivariate statistics based on obtained VIP values revealed asparagine, LPG 22:6 sn-2, orotidine, dimethylglycine, arginine, LPE 22:4 sn-2, aspartic acid, glutathione disulfide, lysine, glutamic acid, LPI 20:4 sn-2, cyclic adenosine monophosphate, LPC 22:4 sn-2, LPI 22:6 sn-2 as key contributors, in descending order.

Summarizing the extensive assessment of the 176 compounds and their influence on the investigated cecal metabolome in the mouse model, a key observation is the central role of LPE 16:1 sn-2. It demonstrated alterations in 7 out of 10 total comparisons across the five groups, followed by 6 out of 10 for LPE 18:1 sn-2, maleic acid, n-linoleoyl taurine, uracil, and 5 for asparagine, cytidine, and glutamic acid. Most importantly, in the specific assessment of treatment (G4-G1, G4-G2, G4-G3), there were no significant alterations in the metabolites and lipids detected using RP-LC-HRMS/MS, suggesting that these metabolites are not key in the mechanism of action or cecal response to treatment.

### 3.4 Untargeted GC-MS Analysis

Finally, the last approach used for the analysis of cecal samples was the untargeted GC-MS analysis Table S7.). A panel of 59 metabolites was detected and identified using AMDIS software followed by further processing to gain peak areas for relative comparison with the Gavin 3.0 script in Matlab. From the detected compounds, α-linolenic acid, arachidonic acid, cholesterol, citric acid, glucose, ornithine, glycine, inosine, glutamic acid, leucine, lysine, valine, serine, tryptophan, tyrosine, uracil, uridine, deoxycholic acid, methyl palmitoleate (16:1) orthophosphate, phosphoethanolamine, pipecolic acid were excluded from this data set, since they were previously detected with the RP-LC-HRMS/MS method. Similarly, hypoxanthine and putrescine were detected by the HILIC-MS/MS methods hence they were also excluded. Pentadecane was used for the normalization of the obtained peak areas before statistical analysis. The stability of the analytical system was confirmed using QC samples as previously described.

The untargeted GC-MS analysis revealed just three significantly different metabolites when evaluating the impact of *C. difficile* (G4-G5): Ethyl alpha-d-glucopyranoside, Glycerol, and Oleic acid. Ethyl alpha-d-glucopyranoside in particular displayed statistical significance and also had a VIP value above the preset criterion of VIP ≥ 1.5.

Upon investigating differences among treatments and infected mice (G4-G1, G2, G3), Metronidazole treatment had the most pronounced impact, with 12 altered metabolites (allose, lactose, galactaric acid, gluconic acid, γ-lactone, glycerol, hydroxyoctanoic acid, arabitol, threonine, methyl aminoacetate, oleic acid, palmitic acid, ribonolactone). This was followed by the FMT treated group with 9 differentiated compounds (2-palmitoylglycerol, glycerol monostearate, hydroxyoctanoic acid, methyl aminoacetate, myo-inositol, oleic acid, palmitic acid, ribitol, stearic acid), and last the probiotic-treated group with only 6 altered metabolites (allocholic acid, lactose, galactaric acid, arabitol, methyl aminoacetate, myo-inositol). However, in multivariate analysis, although statistically significant discriminant models were constructed for Metronidazole and probiotics compared to infected mice (Table S.8), VIP values did not surpass the preset criterion of VIP ≥ 1.5.

Furthermore, the untargeted approach, consistent with the findings described above, indicated that even after a 10-day treatment, the cecal metabolome did not fully recover. In the metronidazole group, 20 metabolites out of 36 (allose, arachidic acid, fructose, lactose, ribulose, xylose, ethyl alpha-d-glucopyranoside, galactaric acid, gluconic acid, γ-lactone, hydroxyoctanoic acid, arabitol, galactose, sorbose, threonine, methyl alpha-D-ribofuranoside, methyl aminoacetate, palmitic acid, propane-1,3-diol, ribitol, ribonolactone) showed differentiation from the control group, while the probiotics and FMT groups demonstrated distinctions in 18 (allose, arachidic acid, cholestan-3-ol, fructose, galactose, lactose, ethanolamine, ethyl alpha-d-glucopyranoside, galactaric acid, gluconic acid, γ-lactone, arabitol, sorbose, methyl aminoacetate, myo-inositol, propane-1,3-diol, ribitol, ribonolactone, stearic acid) and 12 metabolites (2-palmitoylglycerol, allose, arachidic acid, cholestan-3-ol, galactose, xylose, ethanolamine, ethyl alpha-d-glucopyranoside, methyl aminoacetate, ribitol, ribonolactone, stearic acid), respectively. Notably, ethyl alpha-d-glucopyranoside consistently stood out, passing the VIP threshold in all three comparisons.

The impact of allose and lactose, as well as galactaric acid, arabitol, and methyl aminoacetate, was highlighted, as they were statistically significant in multiple comparisons. Allose and lactose showed significance in 5 comparisons out of 10, while the rest of the compounds demonstrated significance in 6 comparisons out of 10. These findings highlight the importance of these specific metabolites in understanding the metabolic response of the cecum to CDI and to treatments.

## 4 Discussion

While discerning the impact of *C. difficile* infection on the metabolome in CDI human patients proves to be challenging, employing murine models under stable and controlled conditions offers a more feasible approach for interpretation, albeit with inherent limitations. For instance, preinfection administration of antibiotics is necessary to render the gut microflora susceptible to colonization, introducing a confounding variable.

Through the application of four distinct metabolomic methodologies covering a metabolic profile of nearly 300 metabolites, the alterations in the cecal metabolome associated with *C. difficile* colonization have been investigated for the first time. Furthermore, the effects of three prevalent treatment strategies—antibiotics, probiotics, and FMT—have been explored using a murine model. The present findings not only contribute novel insights into the cecal metabolome dynamics but also complement our prior research focused on the brain metabolome within the same murine model.

**Figure 2:**
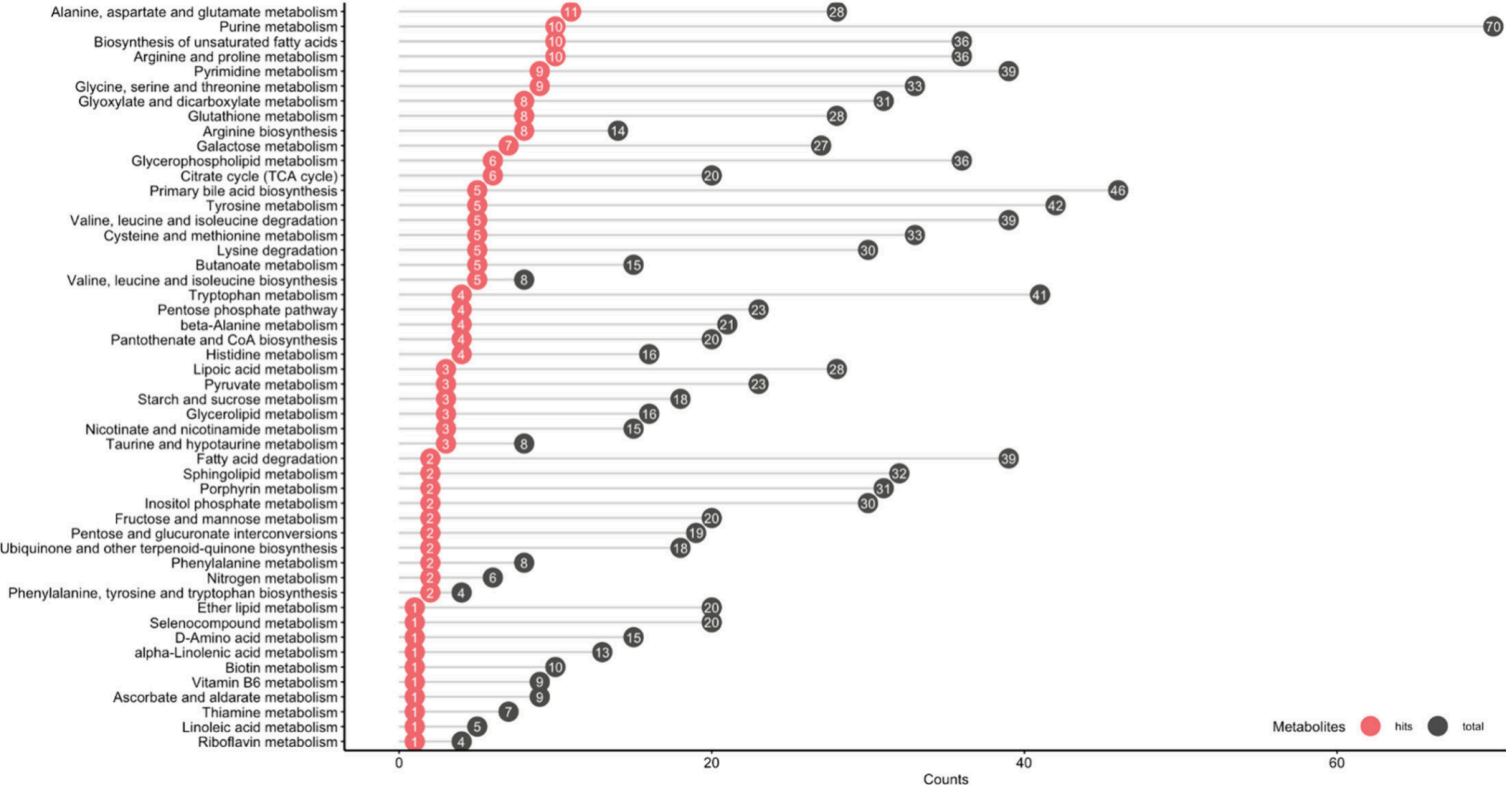
Enrichment analysis of the compounds included in the panel derived from the four methods.

The application of a wide variety of analytical techniques proved to be highly beneficial to cover a wider perspective of the metabolome. As evidenced in this research, focusing solely on the metabolites detected through the HILIC-MS/MS method would lead to the conclusion that baseline metabolic state can be restored by probiotics or FMT. Considering the global profile from all techniques this conclusion would not necessarily be drawn. Likewise, combining the results from all techniques enabled global interpretation of data in the context of metabolic pathways (Figure 2.).

**Figure 3:**
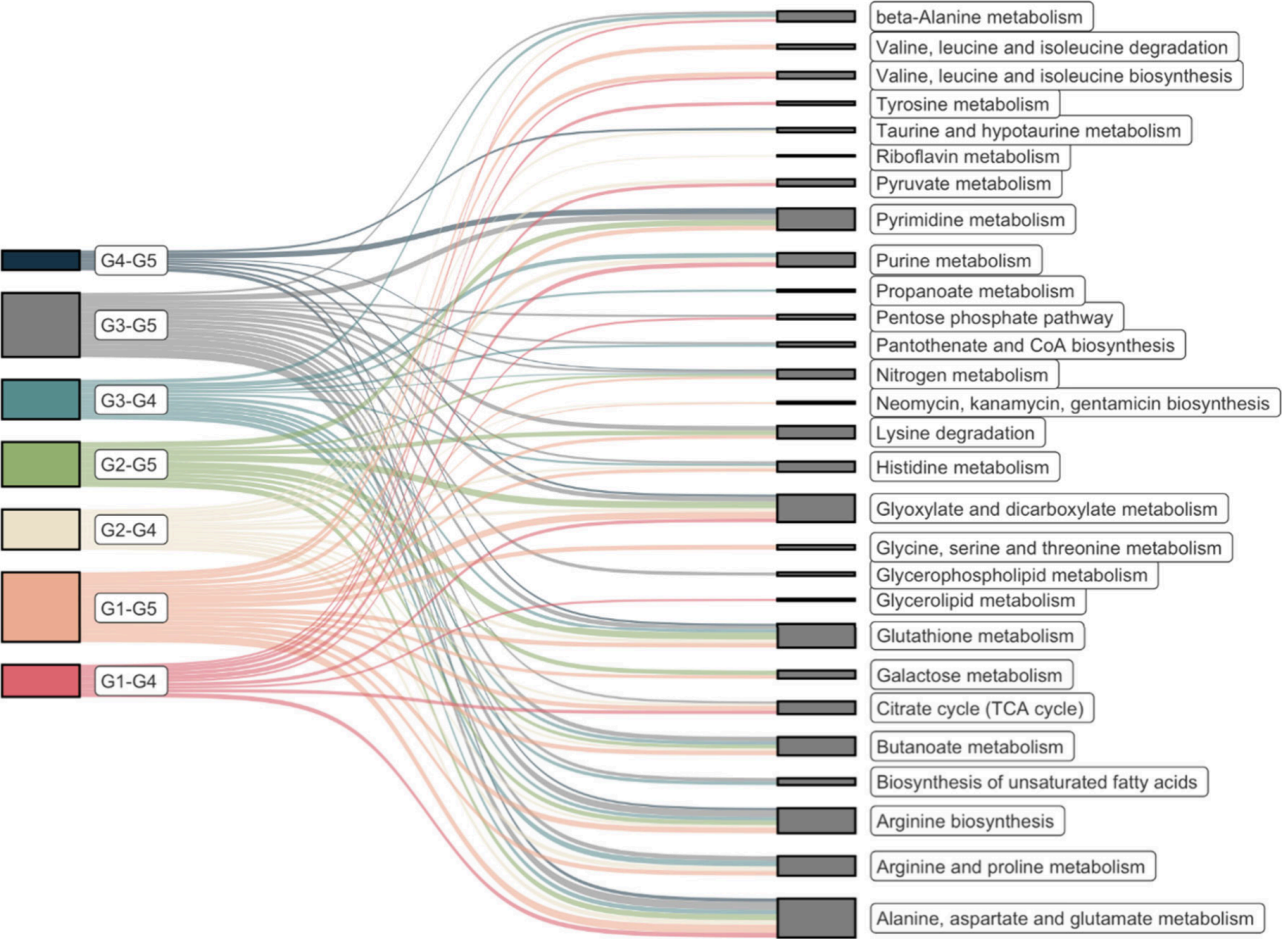
Pathway analysis of the differentiated metabolites in the comparisons between the groups.

To elucidate the potential biochemical pathways responsible for the metabolomic differences between groups, a comprehensive table (Tables 2 and S9.) was constructed, which summarizes the alterations in metabolite profiles detected by the applied methods for each meaningful comparison. Subsequently, pathway analysis was conducted using MetaboAnalyst 6.0 to identify statistically significant pathways (Figure 3).

**Table 2:**
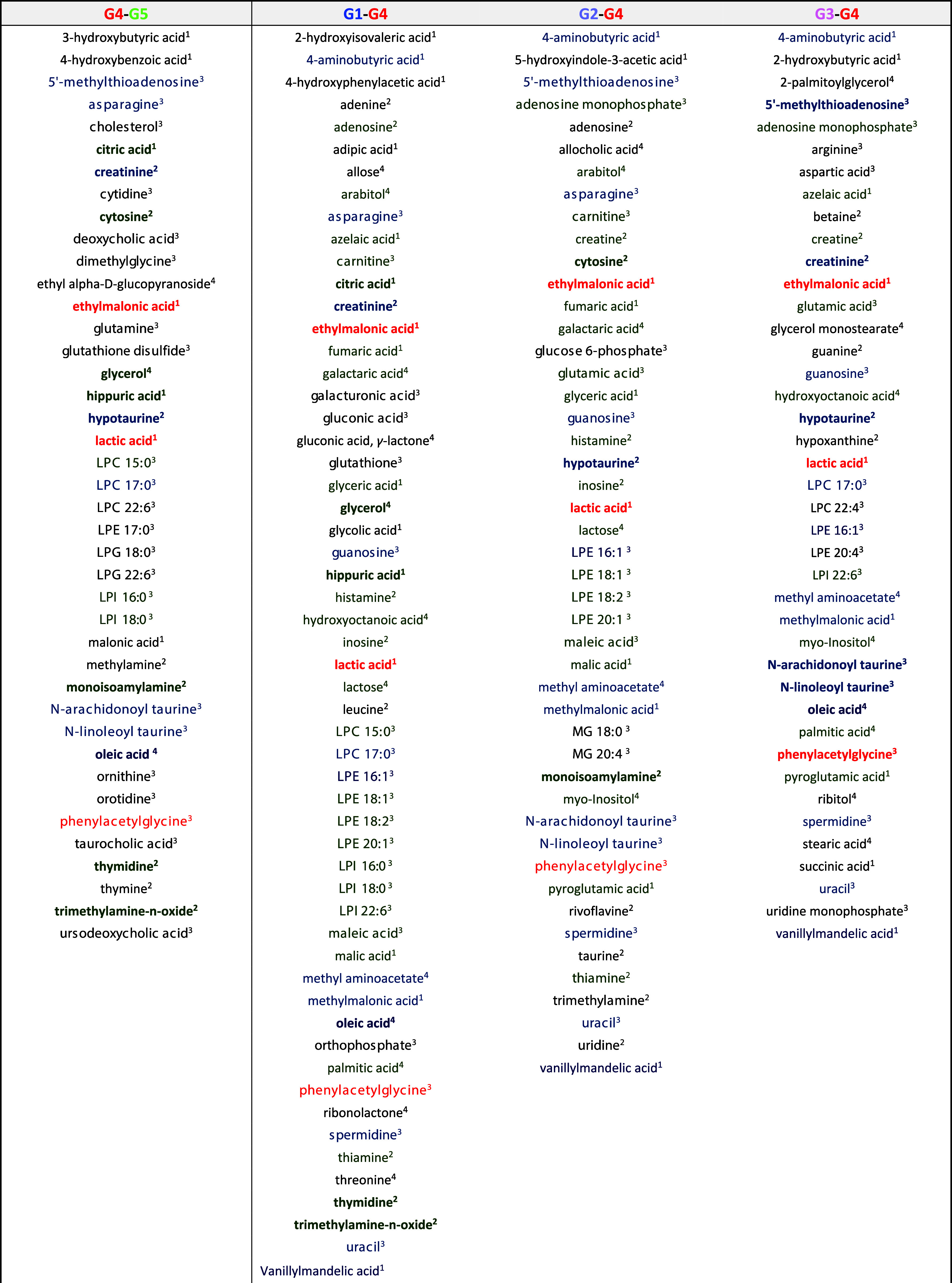
Summary of the Statistically Significant Compounds through Univariate Statistics among the 286 Compounds Analyzed, with Elucidation of the Distinctions between Infected Mice and the Control Group as Well as Variations within Mice Treated with Three Different Approaches (G1: Metronidazole; G2: Probiotics; G3: FMT; and G5: Uninfected Controls)^a^

a ^1^GC-MS/MS. ^2^HILIC-MS/MS. ^3^RP-LC-HRMS/MS. ^4^GC-MS. Color coding indicates significant alterations: red for 4 comparisons, blue for 3, and green for 2. Bold colors highlight compounds significantly changed in the G4-G5 group.

**Figure 4:**
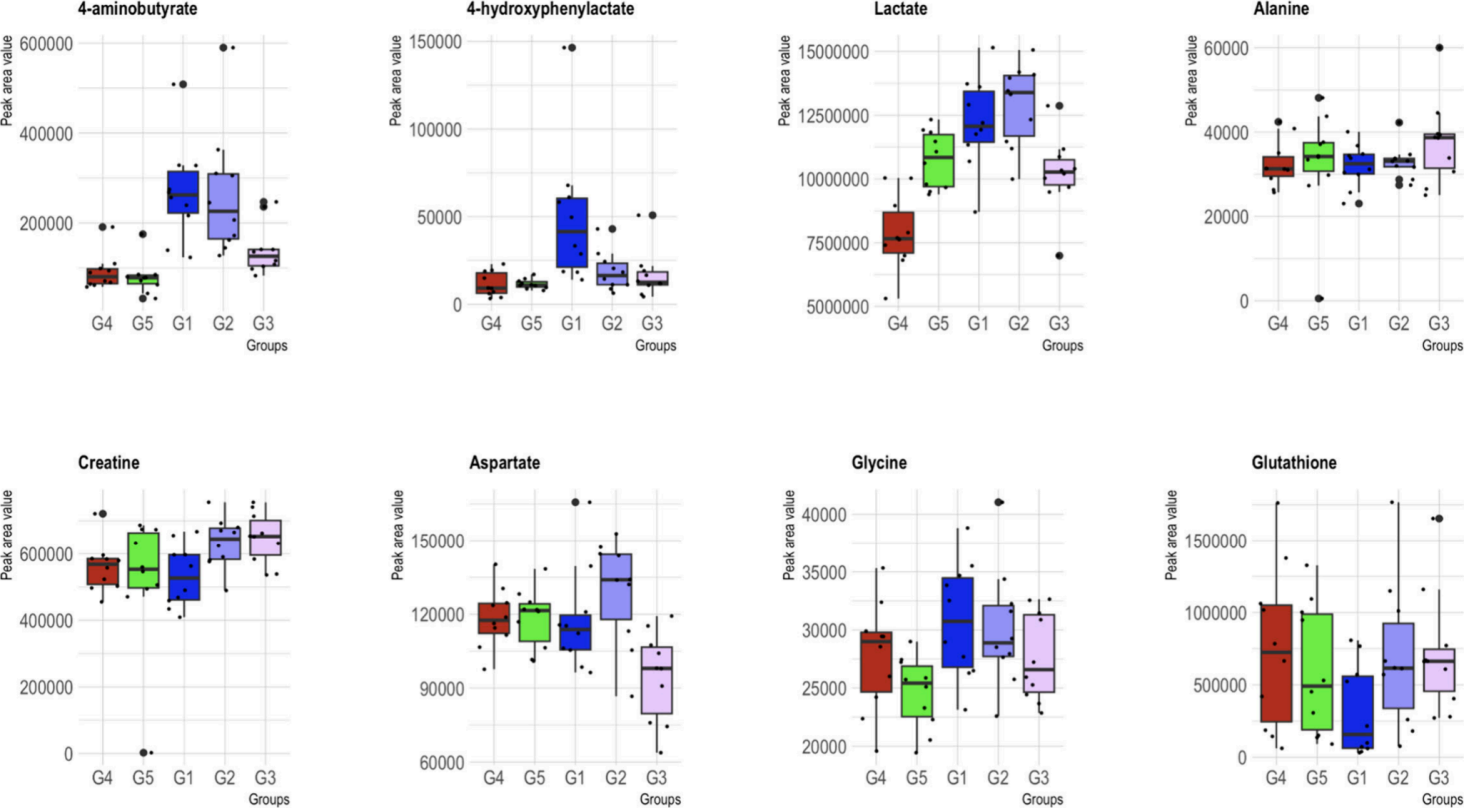
Box plots of the five studied mice groups for eight characteristic metabolites. Group 1 (metronidazole treated) is denoted in blue, Group 2 (probiotics treated) in light blue, and Group 3 (FMT treated) in purple. Group 4 (C. difficile untreated group) is represented in red, and Group 5 (control) is in green.

When investigating the impact of *C. difficile* infection in isolation (Group 4 versus uninfected control Group 5), 44 metabolites exhibited statistically significant differences between the two groups. Notably, the biochemical pathways involving alanine, aspartate, and glutamate metabolism, taurine and hypotaurine metabolism, pyrimidine metabolism and biosynthesis of arginine showed significant p-values. Box plots of characteristic metabolites for each altered pathway are presented in Figure 4.

Upon comparing the untreated *C. difficile* group with the three treatment groups, distinct metabolic perturbations were observed. Specifically, alanine, aspartate, and glutamate metabolism, pyruvate metabolism, purine metabolism and the citrate cycle were significantly affected after metronidazole and probiotics administration. Interestingly, alanine, aspartate, and glutamate, along with arginine and proline metabolism, emerged as common findings for both probiotics and FMT treatments.

The administration of antibiotics impacted pathways related to valine, leucine, and isoleucine biosynthesis, tyrosine metabolism, as well as glyoxylate and dicarboxylate pathways. Conversely, regarding the treatment with probiotics, primary bile acid biosynthesis and taurine and hypotaurine metabolism were statistically significant.

When comparing infected and treated mice with intact controls to assess the extent to which treatment can reverse the effects of infection, several pathways exhibited consistent disturbances. Alanine, aspartate, and glutamate metabolism, glyoxylate and dicarboxylate metabolism, pyrimidine metabolism and arginine biosynthesis were commonly disturbed in all comparisons. Furthermore, citrate cycle and galactose metabolism were disrupted in comparisons of metronidazole and probiotics groups versus controls.

Moreover, alterations in glutathione metabolism and lysine degradation were observed with both probiotics and FMT treatments versus uninfected controls (G2 versus G5 and G3 versus G5 respectively). Additionally, butanoate metabolism was significantly altered in the metronidazole and FMT groups compared to controls, alongside perturbations in glycine, serine, and threonine metabolism, as well as valine, leucine, and isoleucine biosynthesis and degradation, in the metronidazole treated mice (G1 versus G5 comparison). Furthermore, ascorbate and aldarate metabolism, pyruvate metabolism in G2, and arginine and proline metabolism in G3 exhibited significant changes.

The biological significance of these perturbed pathways points toward *C.difficile* metabolism rather than host metabolism. For example, disruptions in valine, leucine, and isoleucine metabolism likely affects the production of Stickland products that are crucial for its growth.^28^ Similarly, perturbations in 4-hydroxyphenylacetate from tyrosine metabolism indicate changes in substrate availability. Moreover, the availability of substrates influences the production of fermentation products such as acetate, valerate, propanoate, butanoate, and 3-hydroxybutanoate, which are derived from acetyl-CoA and/or propanoyl-CoA.^28^ The observed disturbances in the sources of acetyl-CoA, such as threonine, glycine, alanine, and glucose, highlight *C. difficile*’s reliance across diverse metabolic pathways for adaptation within the host environment.

The ecological success of the pathogen is attributed to its adaptability and sophisticated metabolic management. *C. difficile* efficiently exploits abundant gut nutrients, such as amino acids from proteins, utilizing multiple pathways, including amino acid fermentation *via* the Stickland pathway, and carbohydrate catabolism of dietary sugars, with enzymes like pyruvate formate-lyase playing pivotal roles. Alongside the branched-chain and aromatic products of Stickland reactions, *C. difficile* generates various straight-chain organic acids, including acetate, lactate, propionate, and butyrate. It is intriguing to note that alterations in lactate were observed in 5 out of 7 significant comparisons. Pyruvate, sourced from carbohydrates and amino acids, emerges as a crucial metabolite in both fermentation and central carbon metabolism^29^ and our findings indicate alterations in pyruvate levels in response to both *C. difficile* colonization and treatments.

*C. difficile* adapts to nutrient unavailability by utilizing alternative resources, such as phospholipids including phosphatidylethanolamine from both bacterial and host cells, released during cellular damage. Phosphatidylethanolamine is hydrolyzed to ethanolamine and glycerol, while ethanolamine is broken down into ammonia and acetaldehyde, providing nitrogen and supporting biosynthetic pathways. Ammonia serves as a nitrogen source, and acetaldehyde can be converted to ethanol or acetyl-CoA for the TCA cycle.^30^ Glycerophospholipids metabolism was found to be affected, whether studying the impact of *C. difficile* infection, treatment, or return to a basal metabolic state.

The alterations observed in the cecal metabolome cannot solely be attributed to *C. difficile* metabolism but also to the symbiome, involving both the host and gut microbiome. When equilibrium is disrupted and pathogenic bacteria such as *C. difficile* colonize, their metabolic impact is greatly influenced by the gut symbiome. Nutrients derived from the host’s diet, such as amino acids like proline and hydroxyproline, are crucial for *C. difficile* survival *via* the Stickland pathway. When these substrates are depleted, *C. difficile* resorts to the Wood–Ljungdahl pathway to sustain carbohydrate fermentation as a source of energy. Additionally, the production of ornithine by immunometabolism, thought to contribute to the persistence of the infection,^30^ represents another adaptive strategy employed by *C. difficile* to maintain homeostasis, which is in accordance with our findings.

Furthermore, recent research^31^ has revealed additional adaptive mechanisms employed by *C. difficile* for survival. The bacterium produces p-cresol, a molecule with antibacterial effects, particularly against Gram-negative bacteria. The hpdBCA operon plays a pivotal role in fermenting tyrosine to p-hydroxyphenylacetate, providing *C. difficile* with a competitive advantage in the gut environment. Our findings align with this, as changes in tyrosine and p-hydroxyphenylacetate concentrations were observed following metronidazole treatment.

Based on our findings, a significant shift toward anaerobic metabolism could be speculated to favor *C. difficile* infection.^32^ Metabolites such as 3-hydroxybutyric acid and 2-hydroxybutyric acid, which are ketone bodies, and altered levels of succinate, along with amino acids involved in Stickland fermentation, and bile acids like deoxycholic and ursodeoxycholic acid are commonly associated with anaerobic conditions in the gut.

While the healthy gut microbiota is dominated by obligate anaerobes, inflammation and infection increase facultative anaerobes, resulting in dysbiosis.^33,34^ Prior to *C. difficile* infection, beneficial bacteria such as butyrate producers are diminished, predisposing the host to an inflammatory state.^35,36^

There have only been a small number of metabolomics studies to date that have focused on cecal tissue to improve our understanding of the gut microbiome response to bacterial infection and the interaction of therapeutic treatments. Previous research has highlighted that antibiotic choice significantly affects CDI outcomes; for instance, cefoperazone and streptomycin increased CDI susceptibility and altered metabolites like Stickland fermentation substrates.^5^ LC/MS-based metabolomics showed cefoperazone’s differential impact on cecal microbiota in WT and KO mice, with metabolites such as GABA and riboflavin playing a role in CDI.^6^ Antibiotic treatments promoted *C. difficile* growth through gut microbiome changes, impacting amino acids and carbohydrates.^7^ Furthermore, tryptophan metabolism influenced by *C. difficile* affected mucosal damage and IFN-γ production.^8^

The present study has employed a comprehensive multimethod metabolomics approach and marks the first exploration of these effects directly on cecum tissue extracts using advanced analytical platforms.

While we utilized four distinct metabolomic methods, the interpretation of metabolomic changes can be influenced by the sensitivity and specificity of these platforms. Some metabolites, particularly those present at low concentrations, may not have been detected, potentially limiting our ability to capture the full spectrum of metabolic perturbations.

Looking forward, our research trajectory transcends this individual study. We intend to amalgamate the findings from this investigation with prior publication centered on brain tissue metabolomics and anticipated research on fecal metabolites. This integrative endeavor will serve as the foundation for a systematic meta-analysis with an emphasis on elucidating the complexities of the gut-brain axis in the context of *Clostridium difficile*-associated pathologies. Through the harmonization of these disparate data sets, our overarching objective is to furnish a nuanced and mechanistic understanding of the interplay between *C. difficile* colonization, its therapeutic interventions, and their collective impact on gut-brain interaction metabolomics.

## Supporting Information Available

Table S1: p-values, log2 fold changes, organic acids Table S2: Model characteristics, targeted GC-MS/MS Table S3: p-values, log2 fold changes, polar compounds Table S4: Model characteristics, targeted HILIC-MS/MS Table S5: p-values, log2 fold changes, untargeted RP-LC-HRMS/MS Table S6: Model characteristics, untargeted RP-LC-HRMS/MS Table S7: p-values, log2 fold changes, untargeted GC-MS Table S8: Model characteristics, untargeted GC-MS Table S9: Statistically significant compounds, univariate analysis Figure S1: OPLS-DA score plots, targeted GC-MS/MS Figure S2: OPLS-DA score plots, untargeted RP-LC-HRMS/MS (PDF)
