# Supplementary material for: Unraveling Cecal Alterations in Clostridioides difficile Colonized Mice through Comprehensive Metabolic Profiling
Source: J Proteome Res. 2024 Oct 31;23(12):5462–75. doi: 10.1021/acs.jproteome.4c00578 (PMC11629376; doi:10.1021/acs.jproteome.4c00578)
Supplement: Supplementary file 1 — pr4c00578_si_001.pdf [file pr4c00578_si_001.pdf]

Unraveling Cecal Alterations in *Clostridioides difficile* Colonized Mice through Comprehensive Metabolic Profiling

Olga Deda*[1,2], Emily G. Armitage[3], Thomai Mouskeftara[1,2], Melina Kachrimanidou[4], Ioannis Zervos[5], Andigoni Malousi[6], Neil J. Loftus[3], Ioannis Taitzoglou[7], Helen Gika[1,2]*

[1] Laboratory of Forensic Medicine & Toxicology, Department of Medicine, Aristotle University of Thessaloniki, 54124 Thessaloniki, Greece

[2]Biomic AUTh, Center for Interdisciplinary Research and Innovation (CIRI-AUTH), Balkan Center B1.4, 10th km Thessaloniki-Thermi Rd., GR 57001 Thessaloniki, Greece

[3]Shimadzu Corporation, Manchester M17 1GP, UK

[4]1st Laboratory of Microbiology, Department of Medicine, Aristotle University of Thessaloniki,
54124 Thessaloniki, Greece

[5] Laboratory of Animal Physiology, Faculty of Veterinary Medicine, School of Health Sciences, Aristotle University of Thessaloniki, 54124 Thessaloniki, Greece

[6]Laboratory of Biological Chemistry, Department of Medicine, Aristotle University of Thessaloniki, 54124 Thessaloniki, Greece

[7]Laboratory of Development-Breeding of Animal Models and Biochemical Research, School of Health Sciences, Aristotle University of Thessaloniki

* Correspondence: oliadmy@gmail.com (O.D.); gkikae@auth.gr (H.G.)

Table S1. displays p and log2 fold change values for each pairwise comparison among the five groups of organic acids detected in targeted GC-MS/MS method. Bold values indicate p ≤ 0.05, while red values indicate p ≤ 0.01. Blue-colored values indicate |Log2 Fold Change| ≥ 1.5………S4-S7

Table S2. outlines the characteristics of the constructed unsupervised and supervised models for pairwise comparisons among the studied groups from the targeted GC-MS/MS method. The first column identifies the groups under comparison, while the second column designates the model type, with N denoting the number of samples. R2X(cum) and Q2(cum) apply to both unsupervised and supervised models, whereas R2Y(cum) and CV-ANOVA specifically relate to unsupervised models. Bold indicates the comparisons where supervised models yielded statistically significant results………S7-S8

Table S3. summarizes p-values and log2 fold changes from pairwise comparisons of small polar compounds using targeted HILIC-MS/MS. Bold indicates p ≤ 0.05, red for p ≤ 0.01, and blue for |Log2 Fold Change| ≥ 1.5………S8-S11

Table S4. outlines the characteristics of the constructed unsupervised and supervised models for pairwise comparisons among the studied groups from the targeted HILIC-MS/MS method. The first column identifies the groups under comparison, while the second column designates the model type, with N denoting the number of samples. R2X(cum) and Q2(cum) apply to both unsupervised and supervised models, whereas R2Y(cum) and CV-ANOVA specifically relate to unsupervised models. Bold indicates the comparisons where supervised models yielded statistically significant results………S12

Table S5. presents p-values and log2 fold changes from pairwise comparisons of the untargeted RP-LC-HRMS/MS method. Bold indicates p ≤ 0.05, red for p ≤ 0.01, and blue for |Log2 Fold Change| ≥ 1.5………S13-S28

Table S6. outlines the characteristics of the constructed unsupervised and supervised models for pairwise comparisons among the studied groups from the untargeted RP-LC-HRMS/MS method. The first column identifies the groups under comparison, while the second column designates the model type, with N denoting the number of samples. R2X(cum) and Q2(cum) apply to both unsupervised and supervised models, whereas R2Y(cum) and CV-ANOVA specifically relate to unsupervised models. Bold indicates the comparisons where supervised models yielded statistically significant results………S28-S29

Table S7. presents p-values and log2 fold changes from pairwise comparisons of the untargeted GC-MS method. Bold indicates p ≤ 0.05, red for p ≤ 0.01, and blue for |Log2 Fold Change| ≥ 1.5……S29-S32

Table S8. outlines the characteristics of the constructed unsupervised and supervised models for pairwise comparisons among the studied groups from the untargeted GC-MS method. The first column identifies the groups under comparison, while the second column designates the model type, with N denoting the number of samples. R2X(cum) and Q2(cum) apply to both unsupervised and supervised models, whereas R2Y(cum) and CV-ANOVA specifically relate to unsupervised models. Bold indicates the comparisons where supervised models yielded statistically significant results………..S32-S33

Table S9. compiles the statistically significant compounds identified via univariate statistics among the 286 compounds analyzed, focusing on comparisons between the control group and three distinct treatments (G1: metronidazole; G2: probiotics; G3: FMT; G4: untreated controls)………S33-S36

Figure S1. *OPLS-DA score plots from the targeted GC-MS/MS method illustrate the model constructed to discern between G4 (C. difficile untreated group, represented in red) and three treatments: a. metronidazole (depicted in blue), b. probiotics (light blue), and c. FMT (purple). In each of the three discriminant models, the clear clustering of samples along the first component (t[1]) distinctly highlights alterations in the metabolome post-treatment for C. difficile infection. These results underscore the effectiveness of the treatments in inducing discernible metabolic shifts. Table X distinctly showcases statistically significant and strong group differentiation, as underscored by the CV-ANOVA results in their respective models………..S37-S39*

Figure S2. *OPLS-DA score plots from the untargeted RP-LC-HRMS/MS analysis illustrate the constructed models between the C. difficile-infected mice group treated with three different treatments: a. metronidazole (depicted in blue), b. probiotics (light blue), and c. FMT (purple), compared to the control group. The clear clusters of the groups in every treatment, along with the low CV-ANOVA value characteristic of a strong model, indicate that none of the treatments could restore the metabolome to the baseline level. These results suggest that the changes made to the metabolome after antibiotic treatment, followed by C. difficile infection and subsequent treatment, led to irreversible changes in the cecal metabolome. Table X presents in detail the characteristics of the constructed models……………………………………………………………………S39-S40*

Table S1. displays p and log2 fold change values for each pairwise comparison among the five groups of organic acids detected in targeted GC-MS/MS method. Bold values indicate p ≤ 0.05, while red values indicate p ≤ 0.01. Blue-colored values indicate |Log2 Fold Change| ≥ 1.5.

		G1-G2	G1-G3	G1-G4	G1-G5	G2-G3	G2-G4	G2-G5	G3-G4	G3-G5	G4-G5
2-Hydroxybutyric acid	p	8.39126E-01	1.68273E-01	6.39148E-02	8.99446E-01	1.33224E-01	1.33033E-01	9.33375E-01	**1.56934E-02**	1.37263E-01	9.24752E-02
	log2FC	5.18314E-02	-4.68998E-01	4.81962E-01	3.01072E-02	-5.20829E-01	4.30131E-01	-2.17242E-02	9.50960E-01	4.99105E-01	-4.51855E-01
2-hydroxyisovaleric acid	p	7.92053E-02	6.00837E-01	**4.53232E-02**	1.54919E-01	1.78757E-01	2.26154E-01	3.12865E-01	9.65411E-02	3.54499E-01	6.04635E-02
	log2FC	1.36361E+00	3.84998E-01	**1.74891E+00**	9.99478E-01	-9.78612E-01	3.85305E-01	-3.64131E-01	1.36392E+00	6.14480E-01	-7.49436E-01
2-Ketoglutaric acid	p	1.36319E-01	6.56567E-01	6.59572E-01	1.20132E-01	1.32695E-01	1.33324E-01	9.19353E-01	9.81694E-01	1.01554E-01	1.05148E-01
	log2FC	1.19585E+00	3.10442E-01	2.97478E-01	1.23606E+00	-8.85403E-01	-8.98367E-01	4.02142E-02	-1.29640E-02	9.25617E-01	9.38581E-01
3-methyl-2-oxovaleric acid	p	**4.00475E-02**	7.32948E-02	1.01148E-01	**3.93983E-02**	4.44898E-01	3.82498E-01	9.65631E-01	6.90350E-01	4.28785E-01	3.71993E-01
	log2FC	1.09251E+00	9.53961E-01	8.38494E-01	1.10045E+00	-1.38546E-01	-2.54013E-01	7.94369E-03	-1.15467E-01	1.46490E-01	2.61957E-01
3,4-Dihydroxyhydrocinnmaic acid	p	1.66913E-01	7.23023E-01	2.51382E-01	2.23390E-01	3.22364E-01	5.08755E-01	**4.02319E-02**	3.02457E-01	2.52727E-01	1.62895E-01
	log2FC	**-1.85131E+00**	-6.07704E-01	**-2.79562E+00**	**3.03989E+00**	1.24361E+00	-9.44303E-01	**4.89121E+00**	**-2.18791E+00**	**3.64760E+00**	**5.83551E+00**
3-hydroxybutyric acid	p	7.10035E-01	3.41887E-01	7.17119E-01	**1.30224E-03**	3.40327E-01	5.37558E-01	**4.73460E-03**	6.04993E-01	**4.81389E-04**	**6.20055E-04**
	log2FC	-1.36688E-01	2.46316E-01	9.70824E-02	-1.23437E+00	3.83004E-01	2.33771E-01	-1.09768E+00	-1.49234E-01	-1.48069E+00	-1.33146E+00
3-phenyllactic acid	p	7.01943E-02	6.04508E-01	5.01192E-02	1.06194E-01	1.08447E-01	4.36633E-01	4.95890E-01	7.00011E-02	1.84142E-01	2.03932E-01
	log2FC	**1.65691E+00**	3.97875E-01	**1.97326E+00**	1.37088E+00	-1.25903E+00	3.16350E-01	-2.86032E-01	**1.57538E+00**	9.73000E-01	-6.02381E-01
4-aminobutyric acid	p	9.25888E-01	**9.54835E-03**	**2.54884E-04**	**1.31175E-04**	**2.70377E-02**	**1.45543E-03**	**8.18201E-04**	**4.28927E-02**	**1.47701E-02**	5.19264E-01
	log2FC	3.03093E-02	9.27763E-01	**1.56486E+00**	**1.75896E+00**	8.97453E-01	**1.53455E+00**	**1.72865E+00**	6.37099E-01	8.31200E-01	1.94101E-01
4-hydroxyphenylacetic acid	p	**3.63731E-02**	**3.80931E-02**	**1.06972E-02**	**9.95007E-03**	7.19257E-01	9.98735E-02	6.36186E-02	3.21077E-01	2.77940E-01	9.80747E-01

	log2FC	1.40614E+00	**1.57821E+00**	**2.10742E+00**	**2.10011E+00**	1.72067E-01	7.01274E-01	6.93970E-01	5.29207E-01	5.21903E-01	-7.30455E-03
4-hydroxybenzoic acid	p	**3.30293E-02**	**7.14708E-03**	7.29112E-02	**2.70602E-03**	**4.44740E-02**	7.66761E-01	**7.25113E-03**	1.09679E-01	5.69950E-01	**4.28275E-02**
	log2FC	6.42561E-01	9.95294E-01	5.78143E-01	1.08826E+00	3.52733E-01	-6.44183E-02	4.45694E-01	-4.17151E-01	9.29612E-02	5.10113E-01
5-hydroxyindole-3-acetic acid	p	**8.20627E-03**	5.71005E-01	7.25519E-01	4.17160E-01	5.76482E-02	**1.36417E-02**	**4.98808E-02**	7.79196E-01	8.71730E-01	6.16721E-01
	log2FC	-5.16574E-01	-1.40272E-01	-7.61011E-02	-1.76918E-01	3.76302E-01	4.40473E-01	3.39656E-01	6.41713E-02	-3.66455E-02	-1.00817E-01
Adipic acid	p	1.87188E-01	**5.06520E-03**	**1.02960E-02**	**3.06710E-03**	**2.77443E-02**	8.99759E-02	**1.93617E-02**	2.85122E-01	8.12034E-01	2.39835E-01
	log2FC	8.89354E-01	**3.89624E+00**	**2.54289E+00**	**4.00584E+00**	**3.00688E+00**	**1.65354E+00**	**3.11648E+00**	-1.35334E+00	1.09599E-01	1.46294E+00
Azelaic acid	p	2.66008E-01	6.03830E-01	**2.29142E-02**	6.95745E-02	3.80400E-01	1.13597E-01	3.81039E-01	**5.35128E-03**	**4.11657E-02**	3.37036E-01
	log2FC	3.68399E-01	1.52091E-01	8.16460E-01	5.99751E-01	-2.16308E-01	4.48061E-01	2.31352E-01	6.64369E-01	4.47660E-01	-2.16709E-01
Benzoic acid	p	4.87449E-01	2.89663E-01	3.65218E-01	**4.46011E-02**	7.03487E-01	7.73473E-01	8.84493E-02	9.81377E-01	1.13965E-01	1.51709E-01
	log2FC	9.71360E-02	1.36408E-01	1.33793E-01	3.79689E-01	3.92719E-02	3.66572E-02	2.82553E-01	-2.61473E-03	2.43281E-01	2.45895E-01
Citramalic acid	p	6.76568E-01	6.59467E-01	9.92407E-01	5.96964E-01	8.80555E-01	3.26465E-01	7.25110E-01	3.13833E-01	8.65417E-01	2.23816E-01
	log2FC	2.53605E-01	2.89265E-01	5.50623E-03	3.32286E-01	3.56601E-02	-2.48099E-01	7.86806E-02	-2.83759E-01	4.30205E-02	3.26780E-01
Citric acid	p	3.82192E-01	5.65931E-02	**1.39425E-02**	**1.28233E-04**	4.81876E-01	2.67722E-01	**2.02344E-02**	5.97495E-01	**8.62399E-03**	**1.13060E-02**
	log2FC	4.01281E-01	7.92960E-01	1.01495E+00	**2.19445E+00**	3.91679E-01	6.13669E-01	**1.79317E+00**	2.21990E-01	1.40149E+00	1.17950E+00
Ethylmalonic acid	p	2.18188E-01	**1.91681E-02**	**1.42567E-03**	**3.27763E-02**	2.75321E-01	**2.71543E-02**	4.23070E-01	**1.87776E-02**	6.03841E-01	**1.72737E-02**
	log2FC	3.36870E-01	5.97853E-01	9.44016E-01	5.24841E-01	2.60983E-01	6.07147E-01	1.87971E-01	3.46164E-01	-7.30117E-02	-4.19175E-01
Fumaric acid	p	2.40071E-01	1.03308E-01	**5.82702E-03**	**3.66349E-04**	**4.25033E-02**	**9.41885E-03**	**2.49271E-03**	4.94346E-01	1.15101E-01	2.11562E-01
	log2FC	-3.01783E-01	3.39706E-01	4.90039E-01	7.17177E-01	6.41489E-01	7.91821E-01	1.01896E+00	1.50333E-01	3.77472E-01	2.27139E-01
Glutaric acid	p	4.67717E-01	6.37923E-01	7.00373E-01	3.30742E-01	7.56634E-01	2.56382E-01	7.78953E-01	3.79433E-01	5.32644E-01	1.70194E-01
	log2FC	3.24007E-01	2.10149E-01	-1.83670E-01	4.18335E-01	-1.13858E-01	-5.07676E-01	9.43278E-02	-3.93818E-01	2.08186E-01	6.02004E-01

Glyceric acid	p	9.43451E-02	**2.71194E-03**	**1.08296E-05**	**3.89167E-06**	2.92375E-01	**1.36293E-02**	**6.39510E-03**	6.28556E-02	**2.49681E-02**	6.01239E-01
	log2FC	2.79947E-01	4.81424E-01	7.77118E-01	8.48624E-01	2.01477E-01	4.97171E-01	5.68677E-01	2.95694E-01	3.67200E-01	7.15060E-02
Glycolic acid	p	**4.40806E-02**	**2.46429E-02**	**4.99417E-03**	**9.89063E-03**	4.73950E-01	1.18075E-01	2.56883E-01	4.53664E-01	7.34484E-01	6.56610E-01
	log2FC	3.45017E-01	4.48989E-01	5.75569E-01	5.04072E-01	1.03971E-01	2.30552E-01	1.59055E-01	1.26580E-01	5.50833E-02	-7.14971E-02
Hippuric acid	p	8.80635E-01	1.24012E-01	**3.51926E-02**	6.28008E-01	2.05942E-01	9.45508E-02	5.90573E-01	3.15368E-01	1.87463E-01	**4.17294E-02**
	log2FC	-1.29372E-01	1.36965E+00	**2.25754E+00**	3.35719E-01	1.49903E+00	**2.38691E+00**	4.65091E-01	8.87884E-01	-1.03394E+00	**-1.92182E+00**
Indole-3-acetic acid	p	7.76693E-02	4.87150E-01	8.01165E-02	1.20576E-01	9.65029E-02	9.99860E-01	4.94330E-01	1.03273E-01	1.63815E-01	5.07673E-01
	log2FC	7.29568E-01	2.50568E-01	7.29513E-01	5.63468E-01	-4.79000E-01	-5.51320E-05	-1.66100E-01	4.78945E-01	3.12900E-01	-1.66045E-01
Lactic acid	p	3.93998E-01	**2.72456E-02**	**3.27977E-05**	5.68966E-02	**1.74790E-03**	**8.30568E-07**	**2.67099E-03**	**3.82296E-03**	3.81521E-01	**1.01213E-04**
	log2FC	-8.08751E-02	2.58041E-01	6.31578E-01	1.81531E-01	3.38916E-01	7.12453E-01	2.62406E-01	3.73537E-01	-7.65096E-02	-4.50047E-01
Malic acid	p	7.95968E-01	9.02575E-02	**9.56572E-03**	**1.47767E-03**	8.75925E-02	**1.51116E-02**	**3.23413E-03**	3.38750E-01	5.13182E-02	1.36785E-01
	log2FC	-6.59038E-02	4.12349E-01	5.95943E-01	8.42423E-01	4.78253E-01	6.61847E-01	9.08327E-01	1.83594E-01	4.30074E-01	2.46480E-01
Malonic acid	p	8.34110E-02	7.33333E-01	1.26104E-01	**3.03761E-03**	1.76468E-01	5.34504E-01	**2.31619E-02**	3.29723E-01	2.39994E-01	**2.30470E-02**
	log2FC	-1.48919E+00	-2.80310E-01	-1.06941E+00	9.56395E-01	1.20888E+00	4.19783E-01	**2.44559E+00**	-7.89098E-01	1.23671E+00	**2.02580E+00**
Methylmalonic acid	p	2.56659E-01	9.33705E-02	**1.17483E-03**	**8.27016E-04**	4.42342E-01	**1.26140E-03**	**7.90875E-04**	**5.55366E-04**	**3.92947E-04**	6.10035E-01
	log2FC	2.42395E-01	3.59274E-01	8.56733E-01	9.27898E-01	1.16879E-01	6.14338E-01	6.85503E-01	4.97459E-01	5.68624E-01	7.11648E-02
Methylsuccinic acid	p	4.28102E-01	2.38599E-01	1.03322E-01	1.60477E-01	6.96774E-01	3.88760E-01	5.73574E-01	5.96719E-01	8.74694E-01	6.62047E-01
	log2FC	2.93591E-01	4.29214E-01	5.87135E-01	4.72616E-01	1.35623E-01	2.93544E-01	1.79025E-01	1.57921E-01	4.34016E-02	-1.14519E-01
Pyroglutamic acid	p	2.68004E-01	1.72879E-01	5.43047E-02	5.03710E-02	3.78576E-01	**1.46776E-02**	**1.05771E-02**	**3.91330E-02**	**2.50152E-02**	8.51081E-01
	log2FC	4.60528E-01	6.21622E-01	9.30479E-01	9.52754E-01	1.61094E-01	4.69952E-01	4.92226E-01	3.08857E-01	3.31132E-01	2.22749E-02

Pyruvic acid	p	1.07406E-01	9.79602E-02	2.11980E-01	5.91426E-02	5.57592E-01	6.73813E-01	1.45197E-01	5.17447E-01	2.72274E-01	3.32736E-01
	log2FC	1.15696E+00	1.30864E+00	9.27728E-01	1.49139E+00	1.51677E-01	-2.29234E-01	3.34427E-01	-3.80911E-01	1.82750E-01	5.63661E-01
Succinic acid	p	1.30622E-01	6.43125E-01	2.21368E-01	1.86119E-01	**1.72469E-02**	4.57964E-01	7.17577E-01	**3.89858E-02**	**3.23224E-02**	7.70742E-01
	log2FC	1.36246E+00	-3.09290E-01	9.84329E-01	1.14585E+00	**-1.67175E+00**	-3.78128E-01	-2.16603E-01	1.29362E+00	1.45514E+00	1.61525E-01
Tartaric acid	p	2.77561E-01	4.05606E-01	2.25177E-01	5.64207E-01	7.38632E-01	8.20869E-01	4.00995E-01	5.90657E-01	4.55430E-01	3.80986E-01
	log2FC	4.73155E-01	3.49547E-01	5.70407E-01	-8.04270E-01	-1.23608E-01	9.72521E-02	-1.27742E+00	2.20861E-01	-1.15382E+00	-1.37468E+00
Vanillylmandelic acid	p	9.68638E-01	3.02221E-01	**4.23753E-02**	**4.37380E-02**	2.09086E-01	**1.64495E-02**	**1.84046E-02**	**1.52231E-02**	**3.87207E-02**	8.18090E-01
	log2FC	-7.91444E-03	1.87382E-01	3.80589E-01	4.01065E-01	1.95297E-01	3.88504E-01	4.08980E-01	1.93207E-01	2.13683E-01	2.04759E-02

Table S2. outlines the characteristics of the constructed unsupervised and supervised models for pairwise comparisons among the studied groups from the targeted GC-MS/MS method. The first column identifies the groups under comparison, while the second column designates the model type, with N denoting the number of samples. R2X(cum) and Q2(cum) apply to both unsupervised and supervised models, whereas R2Y(cum) and CV-ANOVA specifically relate to unsupervised models. Bold indicates the comparisons where supervised models yielded statistically significant results.

Model	Type	N	R2X(cum)	R2Y(cum)	Q2(cum)	CV-ANOVA
G1-G2	PCA-X	20	5.59000E-01		2.53000E-01	
G1-G3	PCA-X	20	5.54000E-01		1.38000E-01	
G1-G4	PCA-X	20	5.71000E-01		2.37000E-01	
G1-G5	PCA-X	20	6.83000E-01		1.60000E-01	
G2-G3	PCA-X	20	6.73000E-01		2.02000E-01	
G2-G4	PCA-X	20	6.44000E-01		1.66000E-01	
G2-G5	PCA-X	20	7.40000E-01		3.15000E-01	
G3-G4	PCA-X	20	4.50000E-01		2.36000E-02	
G3-G5	PCA-X	20	6.09000E-01		1.17000E-01	

G4-G5	PCA-X	20	4.53000E-01		1.58000E-01	
G1-G2	OPLS-DA	20	4.51000E-01	7.70000E-01	4.03000E-01	8.38629E-02
G1-G3	OPLS-DA	20	7.22000E-01	9.73000E-01	6.28000E-01	9.75745E-02
**G1-G4**	OPLS-DA	20	6.22000E-01	9.57000E-01	8.49000E-01	**1.09316E-04**
**G1-G5**	OPLS-DA	20	5.80000E-01	8.69000E-01	7.92000E-01	**5.41842E-05**
G2-G3	OPLS-DA	20	4.12000E-01	6.95000E-01	3.87000E-01	9.96880E-02
**G2-G4**	OPLS-DA	20	4.43000E-01	7.92000E-01	5.12000E-01	**2.21971E-02**
G2-G5	OPLS-DA	20	4.09000E-01	8.11000E-01	3.23000E-01	1.83320E-01
**G3-G4**	OPLS-DA	20	4.90000E-01	9.38000E-01	6.89000E-01	**8.58080E-03**
G3-G5	OPLS-DA	20	4.11000E-01	7.82000E-01	3.81000E-01	1.05688E-01
**G4-G5**	OPLS-DA	20	2.59000E-01	8.78000E-01	5.51000E-01	**1.25834E-02**

Table S3. summarizes p-values and log2 fold changes from pairwise comparisons of small polar compounds using targeted HILIC-MS/MS. Bold indicates p ≤ 0.05, red for p ≤ 0.01, and blue for |Log2 Fold Change| ≥ 1.5.

		G1-G2	G1-G3	G1-G4	G1-G5	G2-G3	G2-G4	G2-G5	G3-G4	G3-G5	G4-G5
Acetyl-L-Carnitine	p	7.19074E-01	1.63003E-01	6.31024E-01	1.74916E-01	2.16577E-01	8.52196E-01	2.27368E-01	3.92185E-01	4.63504E-01	2.86228E-01
	log2FC	-5.08776E-02	-1.87013E-01	-8.35674E-02	-3.76643E-01	-1.36136E-01	-3.26898E-02	-3.25766E-01	1.03446E-01	-1.89630E-01	-2.93076E-01
Adenine	p	3.17905E-01	**6.71309E-03**	**6.65772E-03**	**2.12868E-02**	**3.49927E-02**	8.68781E-02	9.40187E-01	2.92547E-01	2.98628E-01	9.49291E-01
	log2FC	-3.93370E-01	-1.23585E+00	-9.40052E-01	-8.82544E-01	-8.42476E-01	-5.46682E-01	-4.89174E-01	2.95794E-01	3.53302E-01	5.75081E-02
Adenosine	p	1.65224E-01	**2.11643E-02**	**4.90492E-03**	**3.48731E-03**	8.87074E-02	**3.30382E-02**	6.83184E-02	9.09133E-01	4.07710E-01	4.31229E-01
	log2FC	-8.93900E-01	-2.05974E+00	-2.14080E+00	-2.52433E+00	-1.16584E+00	-1.24690E+00	-1.63043E+00	-8.10525E-02	-4.64584E-01	-3.83531E-01
Alanine	p	6.39447E-01	1.61243E-01	8.21863E-01	8.92842E-01	2.57485E-01	8.52688E-01	5.13582E-01	2.40962E-01	3.80270E-01	9.96280E-01
	log2FC	-4.55908E-02	-2.28543E-01	-6.33476E-02	-2.66182E-02	-1.82952E-01	-1.77568E-02	1.89725E-02	1.65195E-01	2.01925E-01	3.67293E-02
Anthranilic Acid	p	9.02036E-02	8.45696E-01	4.35348E-01	**4.65929E-02**	8.41753E-02	3.46540E-01	6.13152E-02	3.73002E-01	**4.70970E-02**	1.85964E-01
	log2FC	-5.35400E-01	3.68882E-02	-2.64372E-01	-7.04707E-01	5.72288E-01	2.71028E-01	-1.69307E-01	-3.01260E-01	-7.41595E-01	-4.40335E-01
Betaine	p	4.36638E-01	**3.96428E-03**	8.29540E-01	1.00226E-01	5.79696E-02	3.64713E-01	3.57651E-01	**4.66280E-03**	7.92549E-01	1.01251E-01
	log2FC	-1.15342E-01	-3.69069E-01	-1.23318E-02	-4.29718E-01	-2.53727E-01	1.03010E-01	-3.14375E-01	3.56737E-01	-6.06485E-02	-4.17386E-01

Choline	p	3.10927E-01	3.84846E-01	9.71173E-01	5.37018E-01	9.87220E-01	3.61852E-01	2.75420E-01	4.32484E-01	2.86195E-01	5.50012E-01
	log2FC	-1.07996E-01	-1.10170E-01	-1.51858E-02	1.12445E-01	-2.17367E-03	9.28107E-02	2.20442E-01	9.49843E-02	2.22616E-01	1.27631E-01
Creatine	p	**1.84906E-02**	**1.15204E-02**	5.54051E-01	8.95469E-01	6.61950E-01	**4.91173E-02**	5.48710E-02	**2.80513E-02**	1.01705E-01	6.46388E-01
	log2FC	-2.44812E-01	-2.81101E-01	-5.10657E-02	2.53979E-02	-3.62896E-02	1.93746E-01	2.70209E-01	2.30035E-01	3.06499E-01	7.64636E-02
Creatinine	p	4.29146E-01	1.50614E-01	**4.54417E-02**	5.11274E-01	5.80396E-02	1.77083E-01	4.21497E-01	**7.75905E-03**	4.94530E-01	**2.06272E-02**
	log2FC	-2.33548E-01	4.09818E-01	-7.97881E-01	1.94436E-01	6.43366E-01	-5.64334E-01	4.27984E-01	-1.20770E+00	-2.15382E-01	9.92317E-01
Cytosine	p	**4.74911E-02**	9.63001E-01	2.65828E-01	8.48382E-01	**2.52855E-02**	**2.60064E-02**	4.13698E-01	2.18669E-01	8.86917E-01	**3.66618E-02**
	log2FC	1.40650E+00	2.73848E-02	5.92724E-01	7.71438E-02	-1.37912E+00	-8.13777E-01	-1.32936E+00	5.65339E-01	4.97590E-02	-5.15580E-01
Dimethylamine	p	2.15761E-01	4.08899E-01	1.93959E-01	3.40723E-01	5.36053E-01	8.82349E-01	8.32681E-01	5.60853E-01	7.84337E-01	8.24504E-01
	log2FC	-2.64260E-01	-1.41175E-01	-2.61757E-01	-1.92298E-01	1.23085E-01	2.50251E-03	7.19615E-02	-1.20582E-01	-5.11234E-02	6.94589E-02
Glucose	p	8.27778E-01	2.93101E-01	2.23188E-01	6.72604E-01	3.00039E-01	2.54770E-01	9.80780E-01	6.47756E-01	7.01509E-01	5.48616E-01
	log2FC	5.78229E-02	-2.91584E-01	-4.86828E-01	-1.39510E-01	-3.49407E-01	-5.44650E-01	-1.97333E-01	-1.95243E-01	1.52075E-01	3.47318E-01
Guanine	p	5.22009E-01	**2.83031E-02**	9.31228E-01	4.37302E-01	8.26139E-02	4.46397E-01	5.42985E-01	**2.38567E-02**	3.06005E-01	4.14608E-01
	log2FC	-2.67855E-01	-1.00131E+00	-5.55929E-02	-4.70336E-01	-7.33456E-01	2.12263E-01	-2.02480E-01	9.45719E-01	5.30976E-01	-4.14743E-01
Histamine	p	1.37759E-01	**1.02470E-02**	**3.51601E-04**	**2.85771E-03**	6.34452E-02	**9.28341E-03**	6.05740E-01	9.24217E-01	8.70888E-01	9.26821E-01
	log2FC	-4.45126E-01	-1.21184E+00	-1.27215E+00	-1.27213E+00	-7.66717E-01	-8.27024E-01	-8.27001E-01	-6.03070E-02	-6.02839E-02	2.31221E-05
Hypotaurine	p	1.48648E-01	**1.23278E-02**	1.14237E-01	**3.66384E-03**	7.79937E-01	**4.72937E-02**	4.15717E-01	**1.28892E-03**	8.40273E-01	**1.43077E-04**
	log2FC	-8.93363E-01	-1.02713E+00	6.81878E-01	-9.67361E-01	-1.33770E-01	**1.57524E+00**	-7.39981E-02	**1.70901E+00**	5.97717E-02	**-1.64924E+00**
Hypoxanthine	p	1.73205E-01	**2.07691E-02**	5.67899E-01	6.63934E-01	6.02011E-02	4.21894E-01	7.64599E-01	**4.64768E-02**	4.70480E-01	8.70974E-01
	log2FC	-9.69184E-02	-2.08681E-01	-4.72230E-02	-8.01058E-02	-1.11763E-01	4.96955E-02	1.68126E-02	1.61458E-01	1.28576E-01	-3.28828E-02
Inosine	p	5.38622E-01	9.61486E-01	**2.27616E-02**	2.91496E-01	6.17162E-01	**3.50024E-02**	1.46384E-01	6.11169E-02	3.33002E-01	7.09540E-01
	log2FC	-1.14742E-01	-8.12183E-03	2.96270E-01	2.54341E-01	1.06620E-01	4.11012E-01	3.69083E-01	3.04392E-01	2.62463E-01	-4.19289E-02
Isoleucine	p	1.40695E-01	**9.55759E-03**	8.76906E-02	**2.20414E-02**	1.80487E-01	4.96619E-01	1.98355E-01	6.97777E-01	3.02066E-01	2.31248E-01
	log2FC	-1.42847E-01	-2.86329E-01	-2.57371E-01	-4.93699E-01	-1.43482E-01	-1.14524E-01	-3.50852E-01	2.89580E-02	-2.07370E-01	-2.36328E-01
Leucine	p	**2.93944E-02**	**3.89041E-03**	**2.98074E-02**	**1.69141E-02**	2.55223E-01	5.29492E-01	1.80341E-01	7.51706E-01	2.86325E-01	2.32359E-01

	log2FC	-1.96303E-01	-3.08068E-01	-2.86184E-01	-5.23297E-01	-1.11765E-01	-8.98809E-02	-3.26994E-01	2.18839E-02	-2.15229E-01	-2.37113E-01
Mannose	p	5.90021E-01	2.98462E-01	4.01064E-01	3.49290E-01	5.97209E-01	7.59529E-01	9.68286E-01	6.48218E-01	4.37518E-01	3.88875E-01
	log2FC	4.93487E-01	8.72450E-01	7.18499E-01	1.19557E+00	3.78962E-01	2.25011E-01	7.02087E-01	-1.53951E-01	3.23125E-01	4.77075E-01
Methionine	p	7.31898E-02	5.33919E-01	4.17822E-01	9.39654E-01	5.49492E-01	6.57412E-01	5.39377E-01	8.94615E-01	7.30875E-01	6.46904E-01
	log2FC	-1.60954E-01	-8.55745E-02	-1.41740E-01	-1.37314E-02	7.53795E-02	1.92136E-02	1.47223E-01	-5.61659E-02	7.18431E-02	1.28009E-01
Methylamine	p	9.66945E-01	6.77090E-01	4.65120E-01	7.03875E-02	6.37655E-01	4.77749E-01	**3.40165E-02**	2.01996E-01	1.27017E-01	**2.31354E-02**
	log2FC	2.03082E-02	-1.89292E-01	4.89869E-01	-9.28520E-01	-2.09600E-01	4.69561E-01	-9.48828E-01	6.79161E-01	-7.39228E-01	-1.41839E+00
Monoisamylamine	p	**6.85424E-03**	2.68864E-01	2.37946E-01	**3.04631E-03**	**2.62589E-02**	**7.64707E-03**	1.15243E-01	9.95550E-01	**9.61663E-03**	**2.65342E-03**
	log2FC	1.31564E+00	4.48255E-01	4.74032E-01	**1.68583E+00**	-8.67387E-01	-8.41610E-01	3.70187E-01	2.57770E-02	1.23757E+00	1.21180E+00
Nicotinamide	p	1.24917E-01	1.54830E-01	1.98519E-01	3.65773E-01	4.62315E-01	2.56054E-01	6.61945E-01	4.94315E-01	8.62844E-01	5.91109E-01
	log2FC	**-2.42773E+00**	**-1.59294E+00**	-1.07376E+00	**-1.83895E+00**	8.34787E-01	1.35396E+00	5.88776E-01	5.19176E-01	-2.46011E-01	-7.65187E-01
Nicotinic Acid	p	4.50601E-01	4.32381E-01	6.93191E-01	8.28312E-01	9.31649E-01	1.16738E-01	3.43103E-01	1.07876E-01	4.25467E-01	9.64593E-01
	log2FC	-1.22421E-01	-1.32751E-01	4.19913E-02	5.63510E-02	-1.03296E-02	1.64412E-01	1.78772E-01	1.74742E-01	1.89102E-01	1.43597E-02
Valine	p	4.01968E-01	5.06028E-02	2.87005E-01	2.20383E-01	1.79387E-01	7.14365E-01	5.99241E-01	3.92019E-01	8.92190E-01	5.42854E-01
	log2FC	-8.18681E-02	-2.14390E-01	-1.45359E-01	-2.40010E-01	-1.32522E-01	-6.34905E-02	-1.58142E-01	6.90315E-02	-2.56203E-02	-9.46518E-02
Pantothenate	p	7.99537E-01	6.88582E-02	9.75822E-01	4.87973E-01	8.44227E-02	7.79842E-01	8.13011E-01	7.58221E-02	3.92808E-01	4.92760E-01
	log2FC	-6.13497E-02	-5.05463E-01	-2.64067E-02	-2.31938E-01	-4.44113E-01	3.49430E-02	-1.70588E-01	4.79056E-01	2.73525E-01	-2.05531E-01
Phenylalanine	p	9.47768E-01	2.75491E-01	1.23546E-01	**4.97005E-02**	5.32213E-01	3.39374E-01	1.43505E-01	6.63629E-01	2.24504E-01	3.65422E-01
	log2FC	-1.22458E-02	-1.43926E-01	-2.49310E-01	-4.11931E-01	-1.31680E-01	-2.37064E-01	-3.99685E-01	-1.05384E-01	-2.68005E-01	-1.62621E-01
Proline	p	5.78209E-01	8.81502E-01	6.60775E-01	4.71959E-01	5.19528E-01	3.53027E-01	5.49081E-01	7.93747E-01	4.57797E-01	3.78385E-01
	log2FC	-5.30540E-02	1.66090E-02	4.14249E-03	-1.45161E-01	6.96630E-02	5.71965E-02	-9.21067E-02	-1.24666E-02	-1.61770E-01	-1.49303E-01
Putrescine	p	1.11942E-01	2.03365E-01	3.24973E-01	5.06016E-01	7.78634E-01	6.63077E-01	**7.29692E-03**	8.68250E-01	7.81148E-02	1.57943E-01
	log2FC	-6.33057E-01	-5.17948E-01	-4.92895E-01	1.82658E-01	1.15109E-01	1.40162E-01	8.15715E-01	2.50527E-02	7.00606E-01	6.75553E-01
Pyridoxine	p	4.98155E-01	2.26859E-01	1.77987E-01	7.69271E-01	8.51694E-02	1.10179E-01	9.77310E-01	5.16528E-01	3.66023E-01	2.31034E-01
	log2FC	2.34107E-01	-4.76003E-01	-9.81009E-01	-1.09244E-01	-7.10110E-01	-1.21512E+00	-3.43351E-01	-5.05006E-01	3.66759E-01	8.71766E-01

Riboflavin	p	8.08334E-01	1.18023E-01	1.88182E-01	1.59459E-01	**3.99898E-03**	**1.92882E-02**	3.53313E-01	4.36242E-01	8.81991E-01	7.71888E-01
	log2FC	7.75165E-02	6.28894E-01	4.79703E-01	5.87308E-01	5.51377E-01	4.02187E-01	5.09792E-01	-1.49191E-01	-4.15855E-02	1.07605E-01
Taurine	p	**1.18259E-03**	6.74476E-01	9.24237E-01	4.29133E-01	**1.26455E-02**	**1.01138E-03**	1.55423E-01	6.22306E-01	3.18869E-01	4.81624E-01
	log2FC	-3.87884E-01	-6.11777E-02	-2.28153E-02	1.63449E-01	3.26706E-01	3.65068E-01	5.51333E-01	3.83624E-02	2.24627E-01	1.86265E-01
Thiamine	p	1.89118E-01	**4.96462E-02**	**1.20853E-02**	7.62949E-02	1.66189E-01	**3.09675E-02**	3.17081E-01	7.68861E-01	6.34865E-01	7.48393E-01
	log2FC	-1.34123E-01	-2.84194E-01	-3.21492E-01	-3.80037E-01	-1.50071E-01	-1.87369E-01	-2.45914E-01	-3.72984E-02	-9.58436E-02	-5.85452E-02
Thymidine	p	4.03213E-01	1.05862E-01	**3.21740E-02**	5.86573E-01	3.56170E-01	1.55308E-01	3.32948E-01	6.69618E-01	7.68705E-02	**2.60850E-02**
	log2FC	-1.84295E-01	-4.48870E-01	-5.73981E-01	1.32028E-01	-2.64575E-01	-3.89686E-01	3.16323E-01	-1.25111E-01	5.80898E-01	7.06009E-01
Thymine	p	8.37336E-01	2.64206E-01	8.16639E-02	4.31953E-01	3.78577E-01	1.37538E-01	5.21093E-01	5.68136E-01	1.39661E-01	**4.58055E-02**
	log2FC	-4.37341E-02	-3.05352E-01	-4.93337E-01	1.94930E-01	-2.61618E-01	-4.49603E-01	2.38664E-01	-1.87985E-01	5.00282E-01	6.88266E-01
Trimethylamine	p	9.77733E-01	**2.67313E-02**	5.53589E-02	4.68043E-01	**1.57271E-02**	**3.47113E-02**	6.77676E-01	7.14333E-01	2.04363E-01	3.37653E-01
	log2FC	-8.26867E-03	-8.44133E-01	-6.84576E-01	-3.24050E-01	-8.35864E-01	-6.76307E-01	-3.15781E-01	1.59557E-01	5.20083E-01	3.60526E-01
Trimethylamine-N-Oxide	p	6.32171E-02	2.72663E-01	**2.78446E-02**	5.30314E-01	8.35605E-01	5.81898E-01	**3.60957E-02**	5.51155E-01	1.58587E-01	**1.50435E-02**
	log2FC	-8.25916E-01	-7.17316E-01	-1.07483E+00	3.97329E-01	1.08601E-01	-2.48917E-01	1.22325E+00	-3.57517E-01	1.11464E+00	1.47216E+00
Tryptophan	p	1.33396E-01	1.99545E-01	**7.04411E-03**	6.44217E-02	9.08967E-01	1.03122E-01	2.86330E-01	1.11168E-01	2.55668E-01	7.99555E-01
	log2FC	-1.84326E-01	-1.71132E-01	-3.75265E-01	-4.19557E-01	1.31938E-02	-1.90939E-01	-2.35231E-01	-2.04133E-01	-2.48425E-01	-4.42922E-02
Tyrosine	p	6.49377E-02	1.10375E-01	1.43606E-01	2.19973E-01	8.44339E-01	8.90634E-01	6.59403E-01	7.87914E-01	8.35720E-01	7.07872E-01
	log2FC	-1.92439E-01	-2.12577E-01	-2.02530E-01	-2.54533E-01	-2.01382E-02	-1.00916E-02	-6.20943E-02	1.00466E-02	-4.19561E-02	-5.20027E-02
Uridine	p	8.38893E-01	5.80242E-01	9.82208E-02	5.03941E-02	5.69428E-01	**2.29189E-02**	**1.26404E-02**	5.44592E-02	**4.84151E-02**	4.71487E-01
	log2FC	4.13377E-02	1.17432E-01	3.40827E-01	5.73826E-01	7.60944E-02	2.99489E-01	5.32489E-01	2.23395E-01	4.56394E-01	2.32999E-01
Xanthine	p	4.08189E-01	6.53243E-01	5.22051E-01	8.06711E-01	1.74823E-01	7.86297E-01	7.85321E-01	2.01211E-01	6.10551E-01	8.93249E-01
	log2FC	1.07836E-01	-5.02562E-02	5.44348E-02	4.83821E-02	-1.58092E-01	-5.34010E-02	-5.94536E-02	1.04691E-01	9.86383E-02	-6.05268E-03

Table S4. outlines the characteristics of the constructed unsupervised and supervised models for pairwise comparisons among the studied groups from the targeted HILIC-MS/MS method. The first column identifies the groups under comparison, while the second column designates the model type, with N denoting the number of samples. R2X(cum) and Q2(cum) apply to both unsupervised and supervised models, whereas R2Y(cum) and CV-ANOVA specifically relate to unsupervised models. Bold indicates the comparisons where supervised models yielded statistically significant results.

Model	Type	N	R2X(cum)	R2Y(cum)	Q2(cum)	CV-ANOVA
G1-G2	PCA-X	20	3.78000E-01		1.01000E-01	
G1-G3	PCA-X	20	6.58000E-01		4.13000E-02	
G1-G4	PCA-X	20	5.51000E-01		1.06000E-01	
G1-G5	PCA-X	20	6.70000E-01		2.52000E-01	
G2-G3	PCA-X	20	5.83000E-01		1.61000E-01	
G2-G4	PCA-X	20	4.34000E-01		1.47000E-01	
G2-G5	PCA-X	20	6.58000E-01		1.21000E-01	
G3-G4	PCA-X	20	6.99000E-01		2.34000E-01	
G3-G5	PCA-X	20	6.62000E-01		1.13000E-01	
G4-G5	PCA-X	20	7.70000E-01		3.48000E-01	
G1-G2	OPLS-DA	20	4.16000E-01	9.05000E-01	6.02000E-01	5.27270E-02
**G1-G3**	OPLS-DA	20	3.70000E-01	8.48000E-01	6.15000E-01	**4.23414E-03**
**G1-G4**	OPLS-DA	20	5.01000E-01	9.09000E-01	6.77000E-01	**1.14466E-02**
**G1-G5**	OPLS-DA	20	7.74000E-01	9.98000E-01	8.91000E-01	**3.42597E-04**
**G2-G3**	OPLS-DA	20	3.42000E-01	7.99000E-01	4.94000E-01	**2.88771E-02**
**G2-G4**	OPLS-DA	20	5.87000E-01	9.67000E-01	8.23000E-01	**9.41539E-04**
**G2-G5**	OPLS-DA	20	7.31000E-01	9.87000E-01	8.23000E-01	**2.19242E-03**
G3-G4	OPLS-DA	20	5.14000E-01	8.62000E-01	4.29000E-01	2.18567E-01
**G3-G5**	OPLS-DA	20	8.09000E-01	9.87000E-01	8.03000E-01	**2.62553E-02**
**G4-G5**	OPLS-DA	20	8.82000E-01	9.99000E-01	9.60000E-01	**2.78399E-04**

Table S5. presents p-values and log2 fold changes from pairwise comparisons of the untargeted RP-LC-HRMS/MS method. Bold indicates p ≤ 0.05, red for p ≤ 0.01, and blue for |Log2 Fold Change| ≥ 1.5. Metabolites with identifications confirmed by comparison of accurate mass, retention time, isotopic distribution and spectral integrity of MS/MS with authentic standard are marked with *.

		G1-G2	G1-G3	G1-G4	G1-G5	G2-G3	G2-G4	G2-G5	G3-G4	G3-G5	G4-G5
2-Aminoadipate*	p	6.48844E-01	2.02759E-01	5.68685E-02	**1.04279E-02**	3.23417E-01	7.18016E-02	**7.87106E-03**	1.90535E-01	**8.17934E-03**	2.28459E-01
	log2FC	1.07970E-01	2.82316E-01	4.86220E-01	7.08999E-01	1.74346E-01	3.78250E-01	6.01029E-01	2.03904E-01	4.26683E-01	2.22778E-01
4-Oxoproline*	p	4.09952E-01	9.69389E-01	5.21153E-01	4.94262E-01	3.44376E-01	1.76271E-01	1.41952E-01	4.93798E-01	4.46645E-01	9.53039E-01
	log2FC	-1.30886E-01	5.04159E-03	1.05164E-01	9.60934E-02	1.35927E-01	2.36050E-01	2.26979E-01	1.00122E-01	9.10518E-02	-9.07049E-03
4-Trimethylammoniobutanoate*	p	2.11818E-01	3.89496E-01	7.29430E-01	**5.81891E-03**	5.96900E-01	4.46114E-01	2.77472E-01	7.20890E-01	**4.61585E-02**	6.39735E-02
	log2FC	4.64246E-01	2.56636E-01	1.21616E-01	9.35672E-01	-2.07610E-01	-3.42630E-01	4.71426E-01	-1.35020E-01	6.79036E-01	8.14056E-01
5'-Methylthioadenosine*	p	2.39246E-01	1.53600E-01	1.97874E-01	1.27535E-01	6.11653E-01	**4.63217E-02**	7.33571E-01	**4.41790E-02**	8.22488E-01	**2.25406E-02**
	log2FC	-2.75491E-01	-4.29976E-01	2.34551E-01	-3.62905E-01	-1.54485E-01	5.10041E-01	-8.74147E-02	6.64526E-01	6.70702E-02	-5.97456E-01
Adenosine monophosphate*	p	8.76507E-01	4.34890E-01	1.70110E-01	6.78578E-01	3.84816E-01	**1.04110E-02**	7.18462E-01	**7.08518E-03**	8.52765E-01	1.34217E-01
	log2FC	-3.80951E-02	-2.04730E-01	3.61686E-01	-1.47591E-01	-1.66635E-01	3.99781E-01	-1.09496E-01	5.66416E-01	5.71390E-02	-5.09277E-01
Adrenic acid	p	9.74617E-01	4.99474E-01	6.75770E-01	1.82567E-01	6.88310E-01	7.66785E-01	5.90181E-01	4.18359E-01	1.91677E-01	8.21467E-01
	log2FC	-8.34812E-03	-1.29791E-01	9.01348E-02	1.41744E-01	-1.21443E-01	9.84829E-02	1.50092E-01	2.19926E-01	2.71534E-01	5.16090E-02
alpha-Linolenic acid	p	3.17247E-01	9.60237E-02	3.00452E-01	1.59429E-01	2.43483E-01	9.74011E-01	5.29247E-01	2.21981E-01	2.04088E-01	5.25594E-01
	log2FC	8.06479E-01	1.54124E+00	8.27689E-01	1.17027E+00	7.34764E-01	2.12101E-02	3.63793E-01	-7.13553E-01	-3.70971E-01	3.42583E-01
Arachidonic acid*	p	6.15324E-01	5.24193E-01	3.02047E-01	8.71032E-01	9.65150E-01	8.61592E-01	5.78165E-01	8.89537E-01	4.91942E-01	2.93300E-01
	log2FC	-7.13142E-02	-7.89717E-02	-9.85907E-02	1.12392E-02	-7.65757E-03	-2.72765E-02	8.25534E-02	-1.96190E-02	9.02110E-02	1.09830E-01
Arginine*	p	4.69464E-01	6.64538E-02	7.85712E-01	7.98142E-01	**1.30981E-02**	6.03284E-01	3.27300E-01	**4.94358E-02**	**8.89320E-04**	9.33904E-01
	log2FC	1.78833E-01	5.12961E-01	7.57498E-02	6.01081E-02	3.34128E-01	-1.03083E-01	-1.18725E-01	-4.37212E-01	-4.52853E-01	-1.56416E-02
Asparagine*	p	3.68913E-01	8.37632E-02	**8.71116E-03**	**2.54584E-03**	5.51124E-02	**1.12662E-02**	1.61508E-01	9.55026E-02	**1.37848E-05**	**5.02098E-06**

	log2FC	-2.42853E-01	3.10617E-01	6.66237E-01	-5.66074E-01	5.53470E-01	9.09090E-01	-3.23221E-01	3.55620E-01	-8.76691E-01	-1.23231E+00
Aspartic acid*	p	2.25657E-01	**1.81757E-02**	9.14351E-01	9.33237E-01	**1.01210E-03**	1.71895E-01	1.64333E-01	**3.14323E-03**	**3.27528E-03**	9.72958E-01
	log2FC	-1.38037E-01	3.12076E-01	-1.02048E-02	-7.93851E-03	4.50113E-01	1.27832E-01	1.30098E-01	-3.22281E-01	-3.20015E-01	2.26624E-03
Butyrylcarnitine	p	7.28967E-02	**2.81326E-02**	7.28246E-02	**5.59210E-03**	3.48245E-01	8.09916E-01	6.23786E-02	5.32924E-01	5.87644E-01	1.93954E-01
	log2FC	-3.51875E-01	-4.74139E-01	-3.82474E-01	-5.39181E-01	-1.22263E-01	-3.05986E-02	-1.87305E-01	9.16646E-02	-6.50421E-02	-1.56707E-01
Carnitine*	p	8.22428E-01	2.82263E-01	**2.31281E-02**	**4.18000E-02**	3.26939E-01	**1.83882E-02**	**4.27145E-02**	1.98228E-01	2.13471E-01	7.77569E-01
	log2FC	1.65580E-02	8.89873E-02	1.90091E-01	2.18103E-01	7.24294E-02	1.73533E-01	2.01545E-01	1.01103E-01	1.29116E-01	2.80124E-02
Cholesterol*	p	8.69687E-01	6.07010E-02	6.34363E-02	**1.07054E-04**	2.40033E-01	3.10279E-01	**1.51759E-02**	7.18629E-01	8.50863E-02	**1.33275E-02**
	log2FC	7.08607E-03	6.46645E-02	5.25342E-02	1.23162E-01	5.75784E-02	4.54481E-02	1.16076E-01	-1.21303E-02	5.84973E-02	7.06276E-02
Cholic acid*	p	2.05988E-01	8.87784E-01	6.00299E-01	8.52482E-01	1.14344E-01	5.27905E-02	1.21749E-01	5.82906E-01	9.43863E-01	6.91752E-01
	log2FC	-7.66408E-01	8.56222E-02	3.24446E-01	1.22100E-01	8.52030E-01	1.09085E+00	8.88509E-01	2.38824E-01	3.64782E-02	-2.02346E-01
Citrulline*	p	1.53763E-01	6.67921E-01	1.50976E-01	**2.81758E-02**	1.51256E-01	8.48061E-01	1.28865E-01	1.64739E-01	**9.50243E-03**	3.18177E-01
	log2FC	4.56590E-01	1.27764E-01	5.07359E-01	7.88862E-01	-3.28827E-01	5.07682E-02	3.32271E-01	3.79595E-01	6.61098E-01	2.81503E-01
cyclic Adenosine monophosphate*	p	1.46799E-01	9.97237E-01	1.67072E-01	7.54245E-02	**2.62187E-02**	8.36311E-01	5.06338E-01	6.19280E-02	**4.71464E-03**	8.56787E-01
	log2FC	1.23757E+00	2.05974E-03	1.45862E+00	1.66679E+00	-1.23551E+00	2.21053E-01	4.29224E-01	1.45656E+00	1.66473E+00	2.08171E-01
Cytidine*	p	5.13801E-01	1.09528E-01	3.36151E-01	**2.94599E-03**	**4.06950E-02**	1.24138E-01	**1.60314E-03**	3.61376E-01	**4.77427E-02**	**4.25183E-03**
	log2FC	-2.32206E-01	5.99184E-01	3.11176E-01	1.54131E+00	8.31390E-01	5.43382E-01	1.77352E+00	-2.88008E-01	9.42126E-01	1.23013E+00
Cytidine monophosphate*	p	8.06644E-01	5.86825E-01	4.13284E-01	7.66865E-01	2.57252E-01	3.49499E-01	5.68307E-01	8.20363E-02	8.87319E-01	2.94004E-01
	log2FC	6.15614E-02	-1.41129E-01	2.33113E-01	-1.00787E-01	-2.02690E-01	1.71551E-01	-1.62349E-01	3.74241E-01	4.03412E-02	-3.33900E-01
Deoxycholic acid*	p	9.51131E-02	**1.42841E-02**	2.41055E-01	**1.51111E-04**	3.27707E-01	6.80418E-01	**6.44041E-03**	1.96827E-01	5.90383E-02	**4.35552E-03**
	log2FC	-6.30451E-01	-8.99311E-01	-4.94154E-01	-1.32819E+00	-2.68859E-01	1.36297E-01	-6.97743E-01	4.05156E-01	-4.28883E-01	-8.34040E-01

Dihomo-gamma-linolenic acid	p	9.80847E-01	3.37356E-01	4.54029E-01	2.28761E-01	5.13814E-01	6.70782E-01	5.02223E-01	7.05713E-01	9.23091E-01	7.01405E-01
	log2FC	4.98219E-03	1.72166E-01	9.94082E-02	1.53900E-01	1.67184E-01	9.44260E-02	1.48918E-01	-7.27575E-02	-1.82656E-02	5.44919E-02
Dimethylglycine*	p	8.88795E-01	1.57994E-01	6.84889E-02	**6.92790E-04**	1.98459E-01	8.62069E-02	**7.89051E-04**	4.65536E-01	**3.81131E-04**	**6.10897E-03**
	log2FC	2.96213E-02	2.70857E-01	3.78989E-01	8.34872E-01	2.41236E-01	3.49368E-01	8.05251E-01	1.08132E-01	5.64015E-01	4.55883E-01
Docosahexaenoic acid*	p	9.96163E-01	9.09805E-01	9.27755E-01	6.71407E-01	9.51550E-01	9.59725E-01	8.70502E-01	8.61406E-01	8.73456E-01	6.49768E-01
	log2FC	6.45236E-04	9.55455E-03	-6.33622E-03	2.20292E-02	8.90931E-03	-6.98145E-03	2.13839E-02	-1.58908E-02	1.24746E-02	2.83654E-02
Docosenamide	p	5.47100E-01	1.29521E-01	4.00024E-01	9.29513E-02	**4.64939E-02**	1.48103E-01	**3.49013E-02**	3.53814E-01	7.62692E-01	2.44637E-01
	log2FC	-8.62586E-02	2.42673E-01	1.09563E-01	2.99485E-01	3.28931E-01	1.95822E-01	3.85744E-01	-1.33110E-01	5.68123E-02	1.89922E-01
Galacturonic acid*	p	**3.10764E-02**	**9.19359E-03**	**2.79512E-02**	**4.24367E-02**	9.63186E-01	9.29813E-01	8.82672E-01	8.80658E-01	8.95561E-01	8.15411E-01
	log2FC	4.31907E-01	4.22607E-01	4.53828E-01	3.96626E-01	-9.30038E-03	2.19207E-02	-3.52810E-02	3.12211E-02	-2.59806E-02	-5.72017E-02
Gluconic acid*	p	8.02208E-02	**5.17858E-03**	**3.37148E-02**	**2.45027E-02**	1.03378E-01	6.12578E-01	6.01257E-01	2.11795E-01	**2.68860E-02**	9.02738E-01
	log2FC	4.20544E-01	7.01336E-01	5.14092E-01	4.98354E-01	2.80792E-01	9.35482E-02	7.78104E-02	-1.87244E-01	-2.02982E-01	-1.57378E-02
Glucose 6-phosphate*	p	1.29758E-01	2.81943E-01	3.76296E-01	**1.65893E-02**	**1.92165E-02**	**1.52506E-02**	**2.85899E-04**	7.44551E-01	2.16957E-01	8.55187E-02
	log2FC	2.11204E-01	-1.73156E-01	-1.24120E-01	-3.58215E-01	-3.84361E-01	-3.35324E-01	-5.69419E-01	4.90364E-02	-1.85058E-01	-2.34095E-01
Glutamic acid*	p	5.85291E-01	4.40656E-01	2.15156E-01	**2.36551E-02**	7.43584E-01	**3.30712E-02**	**1.42420E-03**	**2.90655E-02**	**1.77326E-03**	1.50060E-01
	log2FC	-3.80113E-02	-5.81568E-02	9.16167E-02	1.84018E-01	-2.01455E-02	1.29628E-01	2.22029E-01	1.49774E-01	2.42175E-01	9.24010E-02
Glutamine*	p	5.05454E-01	8.52037E-01	7.60635E-01	**4.49816E-02**	2.89846E-01	2.05588E-01	**3.99230E-03**	8.81064E-01	**2.23676E-02**	**1.96942E-02**
	log2FC	-6.68900E-02	1.80967E-02	2.81680E-02	2.37875E-01	8.49867E-02	9.50580E-02	3.04765E-01	1.00713E-02	2.19778E-01	2.09707E-01
Glutathione*	p	6.43777E-02	**2.82018E-02**	**4.99273E-02**	1.19506E-01	9.11154E-01	8.15201E-01	6.89534E-01	8.82410E-01	5.76833E-01	5.36051E-01
	log2FC	-1.11715E+00	-1.16554E+00	-1.23133E+00	-9.22229E-01	-4.83894E-02	-1.14181E-01	1.94925E-01	-6.57920E-02	2.43314E-01	3.09106E-01
Glutathione disulfide*	p	8.91012E-01	5.25202E-01	4.37417E-01	**1.31029E-02**	6.79641E-01	5.64097E-01	**1.44005E-02**	7.83333E-01	**2.01253E-03**	**2.79802E-03**
	log2FC	3.45698E-02	1.29936E-01	1.88291E-01	-5.91446E-01	9.53664E-02	1.53721E-01	-6.26016E-01	5.83548E-02	-7.21382E-01	-7.79737E-01

Glycine*	p	7.61275E-01	1.36340E-01	1.74878E-01	**3.72623E-03**	2.48373E-01	2.94455E-01	**9.32414E-03**	9.87559E-01	6.25435E-02	9.76134E-02
	log2FC	3.29989E-02	1.53168E-01	1.51585E-01	3.24421E-01	1.20169E-01	1.18586E-01	2.91422E-01	-1.58303E-03	1.71253E-01	1.72836E-01
Guanosine*	p	9.50744E-01	6.98896E-01	**4.66452E-03**	9.19258E-01	7.42683E-01	**5.23785E-03**	8.98481E-01	**8.47275E-03**	7.71373E-01	2.48800E-01
	log2FC	-1.04070E-02	-7.64961E-02	5.96977E-01	3.97450E-02	-6.60891E-02	6.07384E-01	5.01520E-02	6.73473E-01	1.16241E-01	-5.57232E-01
Histidine*	p	6.75599E-01	**3.81213E-02**	1.48538E-01	**1.48043E-02**	**3.00765E-02**	1.80482E-01	**7.69161E-03**	3.85073E-01	5.92003E-01	1.63447E-01
	log2FC	4.16728E-02	2.28644E-01	1.54457E-01	2.71359E-01	1.86971E-01	1.12784E-01	2.29686E-01	-7.41874E-02	4.27147E-02	1.16902E-01
Homoserine*	p	2.10887E-01	**3.09581E-02**	1.16860E-01	**2.79759E-03**	1.41162E-01	5.41037E-01	**7.28284E-03**	4.35702E-01	2.16400E-01	5.19213E-02
	log2FC	1.11259E-01	2.30158E-01	1.58382E-01	3.52141E-01	1.18899E-01	4.71233E-02	2.40882E-01	-7.17754E-02	1.21984E-01	1.93759E-01
Linoleic acid	p	8.91094E-01	1.49716E-01	4.26592E-01	4.27708E-01	3.04239E-01	6.31150E-01	6.40521E-01	3.94968E-01	3.55477E-01	9.69739E-01
	log2FC	2.93791E-02	2.58475E-01	1.27711E-01	1.22825E-01	2.29096E-01	9.83320E-02	9.34457E-02	-1.30764E-01	-1.35650E-01	-4.88623E-03
LPC 14:0 sn-1	p	8.20596E-01	7.10639E-01	3.64148E-01	3.00770E-01	6.13683E-01	6.05544E-01	2.92402E-01	2.81496E-01	5.88790E-01	8.77902E-02
	log2FC	5.26860E-02	-8.14034E-02	1.87766E-01	-2.05363E-01	-1.34089E-01	1.35080E-01	-2.58049E-01	2.69169E-01	-1.23960E-01	-3.93129E-01
LPC 14:0 sn-2	p	8.13353E-01	4.68295E-01	4.70121E-01	9.19458E-01	3.55434E-01	6.33958E-01	7.28246E-01	1.78874E-01	5.07351E-01	3.87642E-01
	log2FC	4.97452E-02	-1.67963E-01	1.47761E-01	-2.00738E-02	-2.17708E-01	9.80161E-02	-6.98189E-02	3.15724E-01	1.47889E-01	-1.67835E-01
LPC 15:0 sn-1	p	9.22966E-02	3.94346E-01	**3.06620E-02**	7.08587E-01	4.14202E-01	7.16514E-01	**4.01208E-02**	2.19397E-01	2.23291E-01	**1.04083E-02**
	log2FC	5.04713E-01	2.36514E-01	6.21009E-01	-8.63242E-02	-2.68199E-01	1.16296E-01	-5.91038E-01	3.84495E-01	-3.22838E-01	-7.07333E-01
LPC 15:0 sn-2	p	8.21731E-02	6.23586E-01	5.77255E-02	8.75092E-01	1.82853E-01	9.43069E-01	6.49901E-02	1.38178E-01	5.22210E-01	**4.52140E-02**
	log2FC	4.48928E-01	1.08278E-01	4.67939E-01	-3.45886E-02	-3.40650E-01	1.90112E-02	-4.83517E-01	3.59661E-01	-1.42867E-01	-5.02528E-01
LPC 16:0 sn-1	p	2.98838E-01	4.58317E-01	1.26267E-01	9.84579E-01	6.97313E-01	7.29165E-01	3.13238E-01	4.12327E-01	4.77793E-01	1.37214E-01
	log2FC	5.23280E-02	3.26005E-02	7.01718E-02	8.39590E-04	-1.97276E-02	1.78438E-02	-5.14884E-02	3.75714E-02	-3.17609E-02	-6.93322E-02
LPC 16:0 sn-2	p	6.53440E-01	6.61523E-01	2.73831E-01	7.35533E-01	9.80267E-01	4.92178E-01	4.06667E-01	4.63780E-01	4.06201E-01	1.32076E-01
	log2FC	7.47646E-02	7.08832E-02	1.89112E-01	-5.30282E-02	-3.88143E-03	1.14348E-01	-1.27793E-01	1.18229E-01	-1.23911E-01	-2.42140E-01
LPC 16:1 sn-1	p	8.43931E-01	7.97507E-01	1.34888E-01	8.56849E-01	9.59287E-01	2.54711E-01	7.34651E-01	2.71403E-01	6.93447E-01	1.33183E-01
	log2FC	4.41220E-02	5.70464E-02	3.47749E-01	-3.84477E-02	1.29244E-02	3.03627E-01	-8.25697E-02	2.90702E-01	-9.54941E-02	-3.86196E-01

LPC 16:1 sn-2	p	5.48918E-01	6.20085E-01	8.78950E-02	3.97437E-01	9.30001E-01	1.93782E-01	7.83617E-01	1.89738E-01	7.27792E-01	2.85631E-01
	log2FC	1.44783E-01	1.24192E-01	4.71059E-01	2.05879E-01	-2.05911E-02	3.26276E-01	6.10958E-02	3.46867E-01	8.16869E-02	-2.65180E-01
LPC 17:0 sn-1	p	9.13597E-02	5.80070E-01	5.51020E-02	3.54222E-01	2.73428E-01	8.90670E-01	**1.87559E-02**	1.99228E-01	1.70841E-01	**1.02716E-02**
	log2FC	4.64222E-01	1.36622E-01	5.05813E-01	-2.08603E-01	-3.27600E-01	4.15915E-02	-6.72825E-01	3.69191E-01	-3.45225E-01	-7.14416E-01
LPC 17:0 sn-2	p	6.53227E-02	8.44601E-01	**2.97591E-02**	2.10790E-01	9.33108E-02	8.65320E-01	**4.42310E-03**	**4.51979E-02**	1.50805E-01	**1.38610E-03**
	log2FC	5.62431E-01	4.92449E-02	6.14946E-01	-2.93776E-01	-5.13186E-01	5.25148E-02	-8.56206E-01	5.65701E-01	-3.43021E-01	-9.08721E-01
LPC 18:0 sn-1	p	1.94789E-01	4.03481E-01	2.08124E-01	7.18570E-01	5.03538E-01	8.61529E-01	3.56580E-01	4.59663E-01	6.97370E-01	3.39134E-01
	log2FC	8.12679E-02	4.08678E-02	9.59034E-02	1.99171E-02	-4.04001E-02	1.46355E-02	-6.13507E-02	5.50356E-02	-2.09506E-02	-7.59862E-02
LPC 18:0 sn-2	p	7.96879E-01	8.33233E-01	5.59041E-01	5.53675E-01	9.30609E-01	7.21032E-01	3.49863E-01	6.15215E-01	3.22263E-01	1.91064E-01
	log2FC	6.25032E-02	4.54956E-02	1.43145E-01	-1.32648E-01	-1.70076E-02	8.06419E-02	-1.95151E-01	9.76495E-02	-1.78143E-01	-2.75793E-01
LPC 18:1 sn-1	p	2.86943E-01	5.68840E-01	9.86876E-02	5.83955E-01	6.37141E-01	5.48790E-01	5.91146E-01	2.92421E-01	9.63488E-01	2.54322E-01
	log2FC	1.50347E-01	7.87286E-02	2.45329E-01	7.21636E-02	-7.16187E-02	9.49815E-02	-7.81838E-02	1.66600E-01	-6.56503E-03	-1.73165E-01
LPC 18:1 sn-2	p	2.25319E-01	5.48651E-01	8.02349E-02	1.21450E-01	5.42846E-01	4.69457E-01	6.61767E-01	2.21932E-01	3.24440E-01	7.64128E-01
	log2FC	2.40799E-01	1.22598E-01	3.83872E-01	3.22630E-01	-1.18201E-01	1.43073E-01	8.18306E-02	2.61274E-01	2.00032E-01	-6.12422E-02
LPC 18:2 sn-1	p	4.97545E-01	9.15478E-01	6.44117E-01	5.57615E-01	3.99242E-01	7.92228E-01	1.98442E-01	5.34586E-01	5.98257E-01	2.69359E-01
	log2FC	1.42659E-01	-2.01255E-02	9.06473E-02	-1.13200E-01	-1.62785E-01	-5.20120E-02	-2.55859E-01	1.10773E-01	-9.30744E-02	-2.03847E-01
LPC 18:2 sn-2	p	4.61119E-01	9.45965E-01	4.04790E-01	6.02478E-01	4.39797E-01	9.56602E-01	7.92468E-01	3.69133E-01	5.98800E-01	7.31643E-01
	log2FC	2.02224E-01	1.68174E-02	2.15487E-01	1.36224E-01	-1.85407E-01	1.32627E-02	-6.59998E-02	1.98669E-01	1.19407E-01	-7.92625E-02
LPC 20:0 sn-1	p	5.16044E-01	1.84161E-01	8.54343E-02	8.42487E-01	7.50790E-01	4.97185E-01	6.44449E-01	5.96078E-01	3.16756E-01	1.64641E-01
	log2FC	1.58020E-01	2.38983E-01	3.43436E-01	3.80218E-02	8.09635E-02	1.85416E-01	-1.19998E-01	1.04453E-01	-2.00961E-01	-3.05414E-01
LPC 20:0 sn-2	p	5.97230E-01	1.83892E-01	2.49504E-01	8.93847E-01	5.29054E-01	5.97755E-01	4.81508E-01	9.54191E-01	8.48957E-02	1.47404E-01
	log2FC	1.59472E-01	3.25074E-01	3.13593E-01	-3.35537E-02	1.65602E-01	1.54121E-01	-1.93026E-01	-1.14805E-02	-3.58627E-01	-3.47147E-01
LPC 20:1 sn-1	p	8.98857E-01	7.25471E-01	3.43990E-01	6.95074E-01	7.04654E-01	5.84731E-01	6.87241E-01	2.79619E-01	9.98893E-01	2.32609E-01
	log2FC	3.24553E-02	-7.54017E-02	1.87005E-01	-7.50849E-02	-1.07857E-01	1.54550E-01	-1.07540E-01	2.62407E-01	3.16800E-04	-2.62090E-01
LPC 20:1 sn-2	p	9.77884E-01	9.94617E-01	2.71327E-01	6.02697E-01	9.81797E-01	3.47521E-01	6.81400E-01	2.46444E-01	5.92502E-01	9.22532E-02

	log2FC	-8.21624E-03	-1.63985E-03	2.79541E-01	-1.22300E-01	6.57639E-03	2.87758E-01	-1.14084E-01	2.81181E-01	-1.20660E-01	-4.01841E-01
LPC 20:4 sn-1	p	6.07831E-01	5.19433E-01	7.66832E-01	9.82523E-01	1.41670E-01	7.01580E-01	5.03371E-01	1.81113E-01	3.83408E-01	6.88709E-01
	log2FC	1.40611E-01	-1.66471E-01	7.36218E-02	5.74333E-03	-3.07082E-01	-6.69894E-02	-1.34868E-01	2.40093E-01	1.72214E-01	-6.78785E-02
LPC 20:4 sn-2	p	4.72262E-01	9.02727E-01	3.69795E-01	1.33328E-01	3.22264E-01	9.38495E-01	3.79447E-01	1.87787E-01	**4.60292E-02**	2.71240E-01
	log2FC	1.91344E-01	-2.91435E-02	2.06762E-01	3.93359E-01	-2.20488E-01	1.54173E-02	2.02015E-01	2.35905E-01	4.22502E-01	1.86597E-01
LPC 20:5 sn-1	p	2.90048E-01	4.12727E-01	4.06071E-01	5.95950E-01	6.65694E-01	6.23718E-01	5.12533E-01	9.78292E-01	7.49068E-01	7.50302E-01
	log2FC	4.14350E-01	2.95232E-01	2.89305E-01	2.02255E-01	-1.19118E-01	-1.25045E-01	-2.12094E-01	-5.92705E-03	-9.29762E-02	-8.70491E-02
LPC 20:5 sn-2	p	1.14271E-01	**4.12582E-02**	6.61841E-02	**4.87124E-02**	5.80836E-01	8.13137E-01	5.02665E-01	7.01104E-01	7.82886E-01	5.87159E-01
	log2FC	5.05424E-01	6.42081E-01	5.63551E-01	7.23849E-01	1.36656E-01	5.81263E-02	2.18424E-01	-7.85297E-02	8.17682E-02	1.60298E-01
LPC 22:4 sn-1	p	9.01385E-01	3.76373E-01	4.12129E-01	7.38568E-01	4.60259E-01	3.67486E-01	6.55755E-01	9.27688E-02	2.21480E-01	6.07006E-01
	log2FC	-2.99603E-02	-2.15414E-01	1.63129E-01	7.25767E-02	-1.85454E-01	1.93090E-01	1.02537E-01	3.78544E-01	2.87991E-01	-9.05528E-02
LPC 22:4 sn-2	p	9.13239E-01	4.90377E-01	1.31056E-01	**3.47736E-02**	4.89173E-01	3.06869E-01	1.20498E-01	**4.48695E-02**	**1.36985E-02**	1.95765E-01
	log2FC	3.05897E-02	-1.70624E-01	3.02435E-01	4.97609E-01	-2.01214E-01	2.71846E-01	4.67019E-01	4.73059E-01	6.68233E-01	1.95174E-01
LPC 22:6 sn-1	p	6.07450E-01	3.15303E-01	6.50464E-01	1.86197E-01	7.34720E-01	3.83461E-01	6.17799E-01	1.37583E-01	8.81537E-01	**4.87554E-02**
	log2FC	-1.20635E-01	-2.01313E-01	7.17462E-02	-2.28389E-01	-8.06783E-02	1.92381E-01	-1.07755E-01	2.73059E-01	-2.70765E-02	-3.00136E-01
LPC 22:6 sn-2	p	6.68305E-01	4.92457E-01	3.20468E-01	3.21038E-01	9.81256E-01	3.52167E-01	3.46076E-01	1.18777E-01	1.21507E-01	9.47459E-01
	log2FC	-1.39751E-01	-1.47414E-01	1.65217E-01	1.73712E-01	-7.66329E-03	3.04969E-01	3.13463E-01	3.12632E-01	3.21127E-01	8.49489E-03
LPC O-16:0	p	7.01213E-01	6.99109E-01	6.21988E-01	9.33862E-01	9.76435E-01	9.92477E-01	6.97903E-01	9.64986E-01	6.99752E-01	6.41379E-01
	log2FC	-1.53609E-01	-1.39612E-01	-1.57804E-01	2.69729E-02	1.39972E-02	-4.19442E-03	1.80582E-01	-1.81916E-02	1.66585E-01	1.84777E-01
LPC O-18:0	p	4.23305E-01	4.77620E-01	4.94945E-01	8.20971E-01	9.49757E-01	7.56706E-01	5.36285E-01	8.15664E-01	5.91428E-01	6.84436E-01
	log2FC	-4.42960E-01	-4.01111E-01	-2.62928E-01	-7.76307E-02	4.18486E-02	1.80032E-01	3.65329E-01	1.38183E-01	3.23480E-01	1.85297E-01
LPC O-18:1	p	5.71741E-01	4.97447E-01	4.95332E-01	9.62541E-01	9.55123E-01	9.46883E-01	6.37060E-01	8.91758E-01	5.74037E-01	6.08365E-01
	log2FC	-2.62813E-01	-2.93798E-01	-2.29594E-01	-1.57125E-02	-3.09852E-02	3.32187E-02	2.47100E-01	6.42040E-02	2.78086E-01	2.13882E-01
LPC O-18:2	p	4.47447E-01	4.58426E-01	2.94404E-01	7.28782E-01	9.28564E-01	9.66030E-01	6.05051E-01	9.50287E-01	6.44470E-01	5.25789E-01

	log2FC	-4.36928E-01	-3.78670E-01	-4.12185E-01	-1.22485E-01	5.82578E-02	2.47429E-02	3.14442E-01	-3.35149E-02	2.56185E-01	2.89699E-01
LPC P-16:0	p	6.42833E-01	9.87463E-01	7.14856E-01	5.94175E-01	7.13767E-01	9.21360E-01	9.56983E-01	7.78201E-01	7.12424E-01	9.54218E-01
	log2FC	-1.86295E-01	6.18637E-03	-1.37057E-01	-1.61938E-01	1.92481E-01	4.92379E-02	2.43562E-02	-1.43243E-01	-1.68125E-01	-2.48817E-02
LPC P-18:1	p	6.75118E-01	6.96596E-01	7.56511E-01	5.36985E-01	9.83057E-01	8.95226E-01	9.92335E-01	8.86828E-01	9.73520E-01	8.77436E-01
	log2FC	-1.76021E-01	-1.88399E-01	-1.09889E-01	-1.71693E-01	-1.23780E-02	6.61324E-02	4.32884E-03	7.85104E-02	1.67068E-02	-6.18036E-02
LPE 16:0 sn-1	p	3.22356E-01	9.94904E-01	3.36037E-01	6.47865E-01	3.72579E-01	9.60689E-01	1.85883E-01	3.88353E-01	6.89116E-01	1.92340E-01
	log2FC	1.12776E-01	-7.11992E-04	1.06633E-01	-4.75640E-02	-1.13488E-01	-6.14362E-03	-1.60340E-01	1.07345E-01	-4.68520E-02	-1.54197E-01
LPE 16:0 sn-2	p	6.98267E-01	9.93482E-01	4.98694E-01	6.03493E-01	6.37734E-01	7.85347E-01	2.97800E-01	3.71930E-01	4.90394E-01	1.21218E-01
	log2FC	9.60062E-02	1.76570E-03	1.51872E-01	-1.09561E-01	-9.42405E-02	5.58660E-02	-2.05567E-01	1.50107E-01	-1.11326E-01	-2.61433E-01
LPE 16:1 sn-1	p	4.69971E-01	6.87300E-01	6.43782E-02	5.66577E-01	7.09849E-01	2.80566E-01	8.30405E-01	1.19263E-01	8.59199E-01	1.58876E-01
	log2FC	1.65592E-01	8.14931E-02	4.49242E-01	1.16935E-01	-8.40985E-02	2.83650E-01	-4.86564E-02	3.67749E-01	3.54421E-02	-3.32307E-01
LPE 16:1 sn-2	p	6.45534E-01	**3.32587E-02**	**3.12663E-03**	**1.13761E-02**	**1.30569E-02**	**1.41414E-03**	**4.72931E-03**	**2.14667E-02**	1.77923E-01	2.25318E-01
	log2FC	-1.08996E-01	4.54167E-01	8.39045E-01	6.10200E-01	5.63163E-01	9.48041E-01	7.19196E-01	3.84878E-01	1.56033E-01	-2.28845E-01
LPE 17:0 sn-1	p	8.13342E-02	5.76974E-01	6.28855E-02	4.35231E-01	2.20173E-01	9.26607E-01	**1.74871E-02**	1.80462E-01	1.92087E-01	**1.27864E-02**
	log2FC	4.77656E-01	1.37011E-01	5.04004E-01	-1.80705E-01	-3.40645E-01	2.63483E-02	-6.58361E-01	3.66993E-01	-3.17717E-01	-6.84710E-01
LPE 17:0 sn-2	p	4.47195E-01	6.33238E-01	1.14685E-01	1.10287E-01	6.88907E-01	3.59206E-01	**1.93794E-02**	1.47164E-01	**2.61580E-02**	**2.49754E-03**
	log2FC	2.26498E-01	1.26194E-01	4.95072E-01	-4.40383E-01	-1.00305E-01	2.68573E-01	-6.66881E-01	3.68878E-01	-5.66577E-01	-9.35454E-01
LPE 18:0 sn-1	p	7.29611E-01	4.72281E-01	2.52296E-01	9.27940E-01	8.15188E-01	5.13899E-01	7.95516E-01	6.19237E-01	5.58949E-01	3.10998E-01
	log2FC	1.91391E-02	3.35904E-02	6.44938E-02	3.87517E-03	1.44512E-02	4.53547E-02	-1.52640E-02	3.09035E-02	-2.97152E-02	-6.06186E-02
LPE 18:0 sn-2	p	4.83287E-01	8.52706E-01	4.61137E-01	4.85752E-01	5.44644E-01	9.90775E-01	1.26701E-01	5.16534E-01	3.14537E-01	1.08458E-01
	log2FC	1.43134E-01	3.55882E-02	1.45208E-01	-1.31124E-01	-1.07546E-01	2.07393E-03	-2.74258E-01	1.09620E-01	-1.66712E-01	-2.76332E-01
LPE 18:1 sn-1	p	4.30378E-01	6.27205E-01	1.29553E-01	3.67393E-01	7.43889E-01	4.39631E-01	9.43197E-01	2.70475E-01	6.75336E-01	4.55495E-01
	log2FC	1.37160E-01	8.05009E-02	2.86987E-01	1.49402E-01	-5.66591E-02	1.49827E-01	1.22417E-02	2.06486E-01	6.89008E-02	-1.37585E-01
LPE 18:1 sn-2	p	9.33170E-01	**1.31660E-02**	**2.66205E-03**	**1.06668E-03**	**3.50047E-02**	**5.90997E-03**	**3.53222E-03**	9.09699E-02	5.84024E-02	7.37489E-01

	log2FC	1.05511E-02	2.65489E-01	4.87382E-01	4.38530E-01	2.54938E-01	4.76831E-01	4.27979E-01	2.21893E-01	1.73040E-01	-4.88523E-02
LPE 18:2 sn-1	p	7.53484E-01	3.59592E-01	6.24098E-01	5.73928E-01	1.98505E-01	8.35883E-01	3.41516E-01	1.54981E-01	6.70240E-01	2.65944E-01
	log2FC	6.77976E-02	-2.06447E-01	1.10384E-01	-1.19397E-01	-2.74245E-01	4.25863E-02	-1.87195E-01	3.16831E-01	8.70497E-02	-2.29781E-01
LPE 18:2 sn-2	p	9.27922E-01	1.44626E-01	**3.62181E-02**	5.66638E-02	9.04777E-02	**1.84214E-02**	**2.82547E-02**	1.82441E-01	3.33298E-01	6.14810E-01
	log2FC	1.74798E-02	2.72409E-01	4.81522E-01	3.94514E-01	2.54929E-01	4.64042E-01	3.77035E-01	2.09113E-01	1.22105E-01	-8.70074E-02
LPE 20:1 sn-1	p	7.76108E-01	7.29323E-01	3.03098E-01	6.95435E-01	5.70374E-01	2.50951E-01	5.45704E-01	5.10871E-01	9.60757E-01	5.49402E-01
	log2FC	-6.07141E-02	6.93067E-02	2.19512E-01	8.01514E-02	1.30021E-01	2.80226E-01	1.40865E-01	1.50205E-01	1.08447E-02	-1.39361E-01
LPE 20:1 sn-2	p	8.78603E-01	2.31369E-01	**9.35403E-03**	**1.79308E-02**	2.13963E-01	**1.19975E-02**	**2.16954E-02**	8.55561E-02	1.60872E-01	6.87111E-01
	log2FC	-2.37429E-02	1.71814E-01	4.29204E-01	3.70382E-01	1.95557E-01	4.52946E-01	3.94125E-01	2.57389E-01	1.98568E-01	-5.88213E-02
LPE 20:4 sn-1	p	6.26496E-01	7.37813E-01	3.14054E-01	2.23534E-01	1.59969E-01	2.91177E-01	1.55484E-01	**3.35643E-02**	**1.82371E-02**	6.72495E-01
	log2FC	9.59295E-02	-6.56864E-02	2.12778E-01	2.67982E-01	-1.61616E-01	1.16849E-01	1.72053E-01	2.78464E-01	3.33668E-01	5.52040E-02
LPE 20:4 sn-2	p	9.02797E-01	1.27256E-01	1.41072E-01	**2.10292E-03**	1.98383E-01	1.88674E-01	**4.59822E-03**	6.71546E-01	**1.44620E-02**	1.66282E-01
	log2FC	1.20567E-02	1.27164E-01	1.70661E-01	3.39065E-01	1.15107E-01	1.58604E-01	3.27008E-01	4.34971E-02	2.11901E-01	1.68404E-01
LPE 22:4 sn-1	p	8.00387E-01	3.51268E-01	5.32276E-01	3.98054E-01	4.00713E-01	2.39675E-01	1.54085E-01	5.11086E-02	**3.26093E-02**	6.96333E-01
	log2FC	-4.89273E-02	-1.82956E-01	1.15984E-01	1.63654E-01	-1.34028E-01	1.64911E-01	2.12581E-01	2.98939E-01	3.46609E-01	4.76699E-02
LPE 22:4 sn-2	p	4.38140E-01	6.15672E-01	4.83472E-01	**1.45558E-02**	6.11506E-01	2.05201E-01	**1.48050E-02**	1.90917E-01	**9.06292E-04**	7.13613E-02
	log2FC	-1.61614E-01	-6.65188E-02	1.16197E-01	4.37024E-01	9.50948E-02	2.77810E-01	5.98637E-01	1.82715E-01	5.03543E-01	3.20827E-01
LPE 22:6 sn-1	p	3.26173E-01	7.36359E-02	9.58544E-01	9.58113E-01	8.45854E-01	3.23290E-01	2.69555E-01	5.54044E-02	**1.94077E-02**	8.95082E-01
	log2FC	-2.32308E-01	-2.72654E-01	-8.02432E-03	7.01998E-03	-4.03467E-02	2.24283E-01	2.39328E-01	2.64630E-01	2.79674E-01	1.50443E-02
LPE 22:6 sn-2	p	4.14193E-01	3.96932E-01	1.37366E-01	**4.79033E-03**	1.67844E-01	7.25590E-02	**9.42269E-03**	3.49100E-01	**9.27750E-03**	2.32414E-01
	log2FC	-1.11743E-01	7.29083E-02	1.63587E-01	2.88218E-01	1.84651E-01	2.75330E-01	3.99961E-01	9.06788E-02	2.15310E-01	1.24631E-01
LPE P-15:0	p	4.07824E-01	4.01300E-01	2.69776E-01	8.49365E-01	8.99458E-01	9.17435E-01	5.06781E-01	7.91670E-01	5.25651E-01	3.70531E-01
	log2FC	2.92502E-01	2.39387E-01	3.38417E-01	4.50129E-02	-5.31150E-02	4.59151E-02	-2.47489E-01	9.90302E-02	-1.94374E-01	-2.93405E-01

LPE P-16:0	p	7.17527E-01	7.46128E-01	4.12407E-01	9.27135E-01	9.64197E-01	8.31239E-01	6.82803E-01	7.81485E-01	7.08600E-01	3.90036E-01
	log2FC	5.20598E-02	4.38862E-02	8.63632E-02	-7.92854E-03	-8.17356E-03	3.43035E-02	-5.99883E-02	4.24770E-02	-5.18147E-02	-9.42918E-02
LPE P-17:0	p	6.58293E-01	7.46739E-01	5.95958E-01	1.70451E-01	5.51473E-01	9.89299E-01	2.01479E-01	5.04900E-01	4.94632E-01	1.47049E-01
	log2FC	1.56829E-01	-9.43785E-02	1.62857E-01	-2.93904E-01	-2.51208E-01	6.02777E-03	-4.50733E-01	2.57235E-01	-1.99526E-01	-4.56761E-01
LPE P-18:0	p	6.78717E-01	7.50012E-01	6.65908E-01	7.60595E-01	9.31604E-01	9.33335E-01	5.69828E-01	9.88762E-01	6.30545E-01	5.24267E-01
	log2FC	6.97598E-02	5.08876E-02	5.35119E-02	-2.59845E-02	-1.88721E-02	-1.62479E-02	-9.57443E-02	2.62423E-03	-7.68722E-02	-7.94964E-02
LPE P-18:1	p	5.48503E-01	9.37149E-01	8.05225E-01	8.88866E-01	6.91635E-01	7.61473E-01	4.92547E-01	9.06401E-01	8.73171E-01	7.35395E-01
	log2FC	1.09651E-01	1.44517E-02	4.09044E-02	-1.44851E-02	-9.51989E-02	-6.87462E-02	-1.24136E-01	2.64527E-02	-2.89368E-02	-5.53895E-02
LPE P-18:2	p	8.76796E-01	6.77675E-01	7.04151E-01	1.65296E-01	8.78907E-01	9.12405E-01	5.61455E-01	9.54526E-01	6.16157E-01	5.35832E-01
	log2FC	-5.51912E-02	-1.16501E-01	-9.80271E-02	-2.46779E-01	-6.13100E-02	-4.28359E-02	-1.91587E-01	1.84741E-02	-1.30277E-01	-1.48751E-01
LPE P-20:0	p	8.82978E-01	7.91906E-01	8.18190E-01	5.53533E-01	7.45766E-01	7.66642E-01	8.31919E-01	9.46329E-01	4.91880E-01	4.54598E-01
	log2FC	-5.55587E-02	9.20903E-02	6.61722E-02	-1.30032E-01	1.47649E-01	1.21731E-01	-7.44728E-02	-2.59181E-02	-2.22122E-01	-1.96204E-01
LPE P-20:1	p	6.76481E-01	9.42174E-01	7.24034E-01	4.06373E-01	7.58068E-01	5.57625E-01	9.71191E-01	7.51544E-01	6.27837E-01	3.36141E-01
	log2FC	-1.59128E-01	-2.23026E-02	9.35663E-02	-1.72601E-01	1.36826E-01	2.52695E-01	-1.34730E-02	1.15869E-01	-1.50299E-01	-2.66168E-01
LPE P-22:1	p	6.41614E-01	7.07156E-01	6.47214E-01	7.27537E-01	5.16300E-01	4.86643E-01	7.70460E-01	9.90309E-01	5.37616E-01	4.61063E-01
	log2FC	-1.96434E-01	1.23455E-01	1.28076E-01	-7.61549E-02	3.19889E-01	3.24510E-01	1.20279E-01	4.62171E-03	-1.99610E-01	-2.04231E-01
LPG 16:0 sn-1	p	1.31639E-01	6.33147E-01	1.38709E-01	7.45032E-01	3.47407E-01	8.33365E-01	1.06396E-01	4.06961E-01	4.80355E-01	1.13415E-01
	log2FC	3.28983E-01	9.20693E-02	2.77661E-01	-5.76703E-02	-2.36913E-01	-5.13220E-02	-3.86653E-01	1.85592E-01	-1.49740E-01	-3.35331E-01
LPG 16:0 sn-2	p	4.57168E-01	9.18605E-01	3.75673E-01	4.01274E-01	4.96590E-01	9.97976E-01	1.35536E-01	4.08682E-01	3.31917E-01	8.23156E-02
	log2FC	2.07992E-01	2.44286E-02	2.08662E-01	-2.03323E-01	-1.83563E-01	6.69515E-04	-4.11316E-01	1.84233E-01	-2.27752E-01	-4.11985E-01
LPG 18:0 sn-1	p	2.15132E-01	8.21275E-01	1.19903E-01	3.59805E-01	3.34135E-01	9.43375E-01	6.51198E-02	2.31554E-01	2.95270E-01	**3.30296E-02**
	log2FC	3.42591E-01	5.33133E-02	3.63442E-01	-2.18951E-01	-2.89278E-01	2.08511E-02	-5.61542E-01	3.10129E-01	-2.72264E-01	-5.82393E-01
LPG 18:0 sn-2	p	6.47175E-01	8.64344E-01	2.99343E-01	2.63203E-01	5.01567E-01	6.46028E-01	1.25768E-01	1.49253E-01	2.77502E-01	**1.99267E-02**
	log2FC	1.27231E-01	-3.91616E-02	2.41271E-01	-2.62519E-01	-1.66393E-01	1.14040E-01	-3.89750E-01	2.80433E-01	-2.23357E-01	-5.03790E-01
LPG 18:1 sn-1	p	2.33801E-01	6.85304E-01	3.32579E-01	6.11778E-01	3.90142E-01	7.03921E-01	4.08608E-01	5.62918E-01	9.31849E-01	5.97789E-01

	log2FC	2.59133E-01	7.66015E-02	1.80746E-01	9.15758E-02	-1.82532E-01	-7.83876E-02	-1.67558E-01	1.04144E-01	1.49743E-02	-8.91701E-02
LPG 18:1 sn-2	p	5.05606E-01	9.44071E-01	2.51883E-01	7.82484E-02	3.82241E-01	6.22245E-01	2.45179E-01	1.39560E-01	**2.40875E-02**	4.79778E-01
	log2FC	1.31288E-01	-1.17039E-02	2.24517E-01	3.51715E-01	-1.42992E-01	9.32286E-02	2.20426E-01	2.36221E-01	3.63419E-01	1.27198E-01
LPG 18:2 sn-1	p	2.00443E-01	3.13917E-01	4.38437E-01	8.34276E-01	6.21363E-01	4.40566E-01	1.58510E-01	7.39716E-01	2.42018E-01	3.36992E-01
	log2FC	4.14513E-01	2.50745E-01	1.75494E-01	-5.15098E-02	-1.63768E-01	-2.39019E-01	-4.66023E-01	-7.52507E-02	-3.02255E-01	-2.27004E-01
LPG 18:2 sn-2	p	5.07957E-01	5.52836E-01	4.61973E-01	7.60870E-01	7.95648E-01	8.75014E-01	6.90352E-01	8.59343E-01	8.13860E-01	7.10640E-01
	log2FC	1.42248E-01	9.28651E-02	1.12727E-01	5.60443E-02	-4.93826E-02	-2.95209E-02	-8.62034E-02	1.98617E-02	-3.68208E-02	-5.66826E-02
LPG 20:4 sn-1	p	7.22221E-01	6.30445E-01	7.71692E-01	2.84896E-01	9.43354E-01	8.73004E-01	6.59330E-01	8.62473E-01	3.63155E-01	3.73153E-01
	log2FC	6.22238E-02	5.11716E-02	3.55066E-02	1.39284E-01	-1.10522E-02	-2.67172E-02	7.70606E-02	-1.56651E-02	8.81127E-02	1.03778E-01
LPG 20:4 sn-2	p	5.65415E-01	5.06735E-01	1.18145E-01	**1.69622E-02**	9.37368E-01	5.10038E-01	1.84916E-01	3.06950E-01	**4.78526E-02**	3.27006E-01
	log2FC	1.12213E-01	9.72017E-02	2.44337E-01	3.86057E-01	-1.50113E-02	1.32124E-01	2.73844E-01	1.47135E-01	2.88855E-01	1.41720E-01
LPG 22:6 sn-1	p	5.86754E-01	9.32367E-02	1.13164E-01	**2.26200E-02**	**4.33064E-02**	5.22246E-02	**1.23842E-02**	9.13276E-01	6.46333E-02	2.15717E-01
	log2FC	-1.09247E-01	2.56461E-01	2.65711E-01	4.02452E-01	3.65709E-01	3.74959E-01	5.11699E-01	9.24994E-03	1.45991E-01	1.36741E-01
LPG 22:6 sn-2	p	8.40870E-01	1.73273E-01	7.19941E-02	**1.86166E-05**	4.10091E-01	2.78217E-01	**2.04071E-02**	4.96048E-01	**6.21939E-05**	**1.83612E-03**
	log2FC	-5.33235E-02	1.74686E-01	2.63723E-01	7.60391E-01	2.28009E-01	3.17047E-01	8.13715E-01	8.90375E-02	5.85706E-01	4.96668E-01
LPI 16:0 sn-1	p	1.26607E-01	1.49401E-01	**9.25711E-03**	1.13959E-01	9.04779E-01	2.29734E-01	9.15020E-01	1.78437E-01	9.79521E-01	1.35127E-01
	log2FC	2.93617E-01	2.71088E-01	5.19118E-01	2.75375E-01	-2.25292E-02	2.25501E-01	-1.82421E-02	2.48030E-01	4.28710E-03	-2.43743E-01
LPI 16:0 sn-2	p	2.64716E-01	1.71748E-01	**4.62815E-02**	7.61590E-01	7.57917E-01	2.08873E-01	3.01677E-01	2.95773E-01	1.69712E-01	**2.94057E-02**
	log2FC	2.44015E-01	2.95277E-01	4.71299E-01	6.30903E-02	5.12613E-02	2.27283E-01	-1.80925E-01	1.76022E-01	-2.32186E-01	-4.08208E-01
LPI 18:0 sn-1	p	1.66891E-01	2.69409E-01	**1.06646E-02**	7.69571E-02	6.33741E-01	2.44468E-01	9.18521E-01	7.08903E-02	4.70103E-01	1.91666E-01
	log2FC	1.84226E-01	1.23277E-01	3.58791E-01	1.97012E-01	-6.09486E-02	1.74565E-01	1.27863E-02	2.35514E-01	7.37349E-02	-1.61779E-01
LPI 18:0 sn-2	p	4.15937E-01	3.15421E-01	8.42711E-02	6.10963E-01	7.47449E-01	1.14936E-01	5.40115E-01	1.52050E-01	3.02303E-01	**3.33258E-02**
	log2FC	1.69427E-01	2.06807E-01	4.24281E-01	1.00173E-01	3.73793E-02	2.54853E-01	-6.92546E-02	2.17474E-01	-1.06634E-01	-3.24108E-01

LPI 18:2 sn-1	p	2.35384E-01	3.28451E-01	2.93680E-01	6.97263E-01	6.17046E-01	8.04735E-01	9.06384E-02	8.33967E-01	1.34804E-01	1.20708E-01
	log2FC	4.28576E-01	3.36619E-01	3.77154E-01	-1.48769E-01	-9.19575E-02	-5.14221E-02	-5.77345E-01	4.05354E-02	-4.85387E-01	-5.25923E-01
LPI 18:2 sn-2	p	1.27052E-01	3.02633E-01	2.07660E-01	9.48064E-01	3.31572E-01	7.19686E-01	9.85550E-02	6.31813E-01	2.52087E-01	1.69889E-01
	log2FC	5.80404E-01	3.63122E-01	4.85226E-01	-2.53860E-02	-2.17282E-01	-9.51781E-02	-6.05790E-01	1.22104E-01	-3.88508E-01	-5.10612E-01
LPI 20:4 sn-1	p	3.66360E-01	6.00748E-01	3.05309E-01	2.41824E-01	3.10418E-01	7.02272E-01	3.85133E-01	2.22717E-01	7.76268E-02	7.43271E-01
	log2FC	3.50501E-01	1.93919E-01	4.16964E-01	4.74089E-01	-1.56582E-01	6.64629E-02	1.23588E-01	2.23045E-01	2.80171E-01	5.71254E-02
LPI 20:4 sn-2	p	2.69945E-01	5.74252E-01	2.06606E-01	9.05406E-02	1.60426E-01	6.89221E-01	1.11394E-01	6.87110E-02	**2.16733E-03**	2.27941E-01
	log2FC	4.40093E-01	2.01922E-01	5.17840E-01	7.46745E-01	-2.38171E-01	7.77475E-02	3.06652E-01	3.15918E-01	5.44823E-01	2.28905E-01
LPI 22:6 sn-1	p	4.20260E-01	2.90545E-01	1.05463E-01	2.09993E-01	6.90593E-01	1.27315E-01	4.43030E-01	1.61524E-01	6.63680E-01	3.17279E-01
	log2FC	2.59082E-01	3.35543E-01	5.38942E-01	4.05047E-01	7.64603E-02	2.79859E-01	1.45965E-01	2.03399E-01	6.95045E-02	-1.33894E-01
LPI 22:6 sn-2	p	3.88089E-01	3.79812E-01	**4.84252E-02**	**2.77952E-02**	9.27001E-01	1.52200E-01	7.55270E-02	**3.90687E-02**	**1.19066E-02**	3.34934E-01
	log2FC	2.96921E-01	2.73091E-01	6.73988E-01	7.85163E-01	-2.38299E-02	3.77066E-01	4.88242E-01	4.00896E-01	5.12072E-01	1.11175E-01
LPS 18:0 sn-1	p	6.43396E-02	3.56822E-01	7.17089E-02	6.65256E-01	3.43956E-01	6.66097E-01	1.37332E-01	4.94876E-01	6.00601E-01	1.79091E-01
	log2FC	3.71730E-01	1.60502E-01	2.84456E-01	6.62543E-02	-2.11228E-01	-8.72739E-02	-3.05476E-01	1.23954E-01	-9.42477E-02	-2.18202E-01
LPS 18:0 sn-2	p	2.62467E-01	3.59917E-01	2.55678E-01	8.77567E-01	7.16436E-01	9.46659E-01	9.31489E-02	7.38011E-01	1.35669E-01	7.24490E-02
	log2FC	4.24040E-01	3.15473E-01	4.03659E-01	-4.71215E-02	-1.08568E-01	-2.03808E-02	-4.71162E-01	8.81868E-02	-3.62594E-01	-4.50781E-01
Lysine*	p	3.00962E-01	1.78192E-01	7.66379E-01	4.75678E-01	6.60774E-01	3.90154E-01	**1.40113E-02**	2.10966E-01	**2.26092E-03**	2.11816E-01
	log2FC	1.37892E-01	1.74490E-01	4.19837E-02	-8.63068E-02	3.65987E-02	-9.59078E-02	-2.24198E-01	-1.32507E-01	-2.60797E-01	-1.28290E-01
Maleic acid*	p	2.92477E-01	**2.24866E-02**	**3.31428E-02**	**1.71951E-02**	**2.63261E-02**	**2.96631E-02**	**1.94292E-02**	9.86482E-01	6.64255E-01	6.74256E-01
	log2FC	-2.71910E-01	4.01191E-01	3.98097E-01	4.87703E-01	6.73101E-01	6.70007E-01	7.59612E-01	-3.09362E-03	8.65116E-02	8.96052E-02
Methyl arachidonate (20:4)	p	1.87740E-01	4.71455E-01	6.90865E-01	**2.65333E-02**	6.26677E-01	3.69721E-01	7.20536E-01	7.26386E-01	3.51856E-01	1.21778E-01
	log2FC	4.01313E-01	2.14200E-01	9.92105E-02	5.15597E-01	-1.87113E-01	-3.02102E-01	1.14284E-01	-1.14990E-01	3.01397E-01	4.16387E-01
Methyl docosahexaenoate (22:6)	p	**4.70638E-02**	1.35659E-01	4.27490E-01	**1.24516E-02**	5.55384E-01	2.58533E-01	5.71914E-01	5.29217E-01	2.34674E-01	1.06934E-01
	log2FC	6.31653E-01	4.60606E-01	2.54427E-01	7.63385E-01	-1.71047E-01	-3.77226E-01	1.31731E-01	-2.06179E-01	3.02778E-01	5.08958E-01

Methyl linoleate (18:3)	p	2.24912E-01	1.64258E-01	4.74055E-01	1.76475E-01	6.84015E-01	4.69802E-01	7.76758E-01	2.97431E-01	8.15496E-01	3.31450E-01
	log2FC	1.26633E+00	1.50413E+00	6.71059E-01	1.42764E+00	2.37797E-01	-5.95274E-01	1.61309E-01	-8.33071E-01	-7.64883E-02	7.56583E-01
Methyl oleate (18:1)	p	9.44917E-02	2.18617E-01	4.18889E-01	1.01703E-01	4.64900E-01	3.38814E-01	7.37008E-01	6.95362E-01	5.67594E-01	3.98906E-01
	log2FC	8.17002E-01	5.32198E-01	3.70510E-01	7.02473E-01	-2.84804E-01	-4.46493E-01	-1.14530E-01	-1.61689E-01	1.70274E-01	3.31963E-01
Methyl palmitoleate (16:1)	p	1.76275E-01	1.27463E-01	4.15272E-01	1.40895E-01	7.58019E-01	5.15269E-01	8.64901E-01	3.71744E-01	8.30416E-01	4.16857E-01
	log2FC	1.16474E+00	1.32309E+00	6.65501E-01	1.25115E+00	1.58355E-01	-4.99238E-01	8.64101E-02	-6.57593E-01	-7.19446E-02	5.85648E-01
MG 14:0 (1)	p	1.26343E-01	1.29576E-01	3.80856E-01	1.01889E-01	8.84484E-01	3.36169E-01	5.09919E-01	3.47645E-01	1.89333E-01	2.45435E-01
	log2FC	2.86193E+00	2.72343E+00	1.15467E+00	3.59506E+00	-1.38502E-01	-1.70726E+00	7.33133E-01	-1.56875E+00	8.71636E-01	2.44039E+00
MG 14:0 (2)	p	6.32575E-02	5.68376E-02	3.23805E-01	**4.14861E-02**	9.27038E-01	3.44390E-01	4.51446E-01	3.16894E-01	2.53703E-01	2.31659E-01
	log2FC	1.96167E+00	2.00993E+00	9.02713E-01	2.36723E+00	4.82592E-02	-1.05896E+00	4.05556E-01	-1.10722E+00	3.57297E-01	1.46452E+00
MG 16:0 (1)	p	1.05022E-01	8.01741E-02	3.97290E-01	7.00090E-02	7.89001E-01	3.98057E-01	6.28374E-01	3.14767E-01	6.50022E-01	2.74628E-01
	log2FC	1.80802E+00	1.99624E+00	8.22752E-01	2.14694E+00	1.88218E-01	-9.85270E-01	3.38922E-01	-1.17349E+00	1.50704E-01	1.32419E+00
MG 16:0 (2)	p	7.21262E-02	3.75634E-02	3.67966E-01	2.88790E-02	7.64354E-01	3.29666E-01	5.96046E-01	1.98868E-01	6.85810E-01	1.53336E-01
	log2FC	6.89386E-01	7.59801E-01	3.42001E-01	8.15141E-01	7.04149E-02	-3.47385E-01	1.25756E-01	-4.17800E-01	5.53407E-02	4.73141E-01
MG 16:1 (1)	p	8.96417E-02	6.13042E-02	3.46369E-01	**4.94877E-02**	7.58008E-01	4.14023E-01	5.57159E-01	3.03523E-01	5.79523E-01	2.45703E-01
	log2FC	2.13371E+00	2.47572E+00	9.74964E-01	2.82640E+00	3.42006E-01	-1.15875E+00	6.92692E-01	-1.50075E+00	3.50686E-01	1.85144E+00
MG 16:1 (2)	p	**4.77023E-02**	**2.51309E-02**	2.50736E-01	**3.15431E-02**	6.60805E-01	4.19354E-01	8.58783E-01	2.73947E-01	6.28896E-01	3.33992E-01
	log2FC	1.99682E+00	2.41297E+00	1.00882E+00	2.15030E+00	4.16154E-01	-9.87994E-01	1.53489E-01	-1.40415E+00	-2.62665E-01	1.14148E+00
MG 18:0 (1)	p	5.04587E-01	7.65485E-01	1.84347E-01	4.30422E-01	2.74014E-01	**1.43019E-02**	1.13308E-01	2.67267E-01	5.85544E-01	6.61969E-01
	log2FC	1.82792E-01	-8.43651E-02	-3.22186E-01	-2.26648E-01	-2.67157E-01	-5.04978E-01	-4.09440E-01	-2.37821E-01	-1.42283E-01	9.55376E-02
MG 18:0 (2)	p	9.35744E-01	3.40577E-01	9.37427E-02	2.42419E-01	3.11941E-01	8.65595E-02	2.22416E-01	4.37854E-01	7.87911E-01	6.35110E-01

	log2FC	1.68760E-02	-1.79832E-01	-3.04881E-01	-2.26829E-01	-1.96708E-01	-3.21757E-01	-2.43705E-01	-1.25049E-01	-4.69973E-02	7.80520E-02
MG 18:1 (1)	p	7.15651E-02	6.85721E-02	2.89974E-01	5.13195E-02	8.98354E-01	4.01815E-01	9.88829E-01	4.14074E-01	8.31931E-01	3.32935E-01
	log2FC	1.53442E+00	1.44112E+00	7.87747E-01	1.54371E+00	-9.33007E-02	-7.46669E-01	9.29684E-03	-6.53368E-01	1.02598E-01	7.55966E-01
MG 18:1 (2)	p	**3.34138E-02**	**2.25118E-02**	2.05677E-01	**4.97631E-02**	9.34446E-01	3.67371E-01	4.96887E-01	3.01807E-01	3.25342E-01	5.82569E-01
	log2FC	1.37698E+00	1.42515E+00	7.40730E-01	1.04904E+00	4.81722E-02	-6.36247E-01	-3.27939E-01	-6.84419E-01	-3.76112E-01	3.08307E-01
MG 18:2 (1)	p	8.35211E-02	5.58256E-02	2.68711E-01	5.00134E-02	8.83923E-01	4.93852E-01	8.53128E-01	3.85807E-01	9.63910E-01	3.59399E-01
	log2FC	1.71298E+00	1.84993E+00	9.57228E-01	1.87494E+00	1.36950E-01	-7.55755E-01	1.61961E-01	-8.92706E-01	2.50108E-02	9.17716E-01
MG 18:2 (2)	p	**4.18367E-02**	**1.89841E-02**	1.80856E-01	**4.66381E-02**	7.66569E-01	4.77116E-01	6.46247E-01	3.10978E-01	2.48758E-01	6.24720E-01
	log2FC	1.55457E+00	1.77956E+00	9.23196E-01	1.26717E+00	2.24988E-01	-6.31374E-01	-2.87399E-01	-8.56362E-01	-5.12387E-01	3.43975E-01
MG 18:3 (1)	p	1.52141E-01	1.22857E-01	4.20502E-01	1.13012E-01	6.47815E-01	4.27191E-01	4.90466E-01	3.31651E-01	5.50450E-01	2.98326E-01
	log2FC	2.60296E+00	3.16994E+00	1.10947E+00	3.52581E+00	5.66984E-01	-1.49349E+00	9.22851E-01	-2.06047E+00	3.55867E-01	2.41634E+00
MG 18:3 (2)	p	9.92046E-02	6.57625E-02	3.51706E-01	7.13001E-02	5.54134E-01	4.42921E-01	6.60033E-01	3.05355E-01	6.62136E-01	3.32193E-01
	log2FC	2.32963E+00	3.00206E+00	1.08399E+00	2.78997E+00	6.72430E-01	-1.24563E+00	4.60348E-01	-1.91806E+00	-2.12082E-01	1.70598E+00
MG 20:4 (1)	p	6.38065E-02	1.87352E-01	3.78809E-01	**4.49356E-02**	5.30476E-01	1.86950E-01	7.06591E-01	5.17548E-01	6.59900E-01	1.49228E-01
	log2FC	8.23549E-01	5.12570E-01	2.79338E-01	6.70572E-01	-3.10980E-01	-5.44212E-01	-1.52978E-01	-2.33232E-01	1.58002E-01	3.91234E-01
MG 20:4 (2)	p	**2.78098E-03**	**1.01083E-02**	3.00069E-01	1.85555E-01	6.39942E-01	**2.18537E-02**	**2.44742E-02**	6.97985E-02	8.45329E-02	8.05684E-01
	log2FC	9.12354E-01	7.49507E-01	2.27151E-01	2.78913E-01	-1.62847E-01	-6.85203E-01	-6.33442E-01	-5.22356E-01	-4.70594E-01	5.17615E-02
MG 22:6 (1)	p	1.75864E-01	1.48715E-01	4.82632E-01	1.22718E-01	8.41124E-01	4.12480E-01	5.91322E-01	3.39914E-01	6.35687E-01	2.66729E-01
	log2FC	1.44751E+00	1.56777E+00	6.71058E-01	1.74721E+00	1.20262E-01	-7.76448E-01	2.99706E-01	-8.96709E-01	1.79445E-01	1.07615E+00
MG 22:6 (2)	p	7.58080E-02	**4.65367E-02**	4.70567E-01	1.00901E-01	6.03028E-01	2.91773E-01	5.10748E-01	1.92355E-01	8.92720E-02	3.91795E-01
	log2FC	1.30682E+00	1.54050E+00	5.01413E-01	1.07475E+00	2.33683E-01	-8.05406E-01	-2.32066E-01	-1.03909E+00	-4.65749E-01	5.73340E-01
N2-Acetyllysine*	p	3.71782E-01	1.03547E-01	1.44726E-01	**1.30654E-02**	2.25408E-01	3.47437E-01	**3.71384E-03**	9.02547E-01	1.22662E-01	1.67323E-01

	log2FC	3.31115E-01	7.00873E-01	6.53420E-01	1.17878E+00	3.69758E-01	3.22305E-01	8.47667E-01	-4.74530E-02	4.77909E-01	5.25362E-01
N-Acetylneuraminic acid*	p	**4.11036E-03**	7.16197E-01	2.32986E-01	**3.38151E-03**	**1.72768E-02**	6.71807E-02	8.91698E-01	4.43690E-01	**1.61304E-02**	6.58038E-02
	log2FC	5.63943E-01	6.53983E-02	2.12023E-01	5.42146E-01	-4.98545E-01	-3.51920E-01	-2.17972E-02	1.46625E-01	4.76748E-01	3.30123E-01
N-arachidonoyl taurine	p	4.27104E-01	2.11308E-01	1.85344E-01	**1.89598E-02**	7.64675E-01	**3.76287E-02**	1.10648E-01	**2.84896E-03**	1.16138E-01	**3.65326E-04**
	log2FC	-1.98624E-01	-2.60185E-01	2.97143E-01	-5.57689E-01	-6.15607E-02	4.95767E-01	-3.59065E-01	5.57328E-01	-2.97504E-01	-8.54832E-01
N-linoleoyl taurine	p	4.10581E-01	3.58471E-01	1.41329E-01	**2.21731E-03**	8.74279E-01	**3.48000E-02**	**2.33359E-02**	**1.89262E-03**	**4.49118E-03**	**2.93658E-05**
	log2FC	-2.86991E-01	-2.42601E-01	4.53134E-01	-9.60135E-01	4.43899E-02	7.40125E-01	-6.73145E-01	6.95735E-01	-7.17535E-01	-1.41327E+00
Oleic acid	p	4.37376E-01	1.94092E-01	3.32913E-01	2.46169E-01	6.79665E-01	9.11058E-01	8.77273E-01	7.33019E-01	6.81737E-01	9.77782E-01
	log2FC	1.61884E-01	2.42967E-01	1.84362E-01	1.88363E-01	8.10835E-02	2.24781E-02	2.64788E-02	-5.86055E-02	-5.46047E-02	4.00075E-03
Ornithine*	p	8.38423E-01	4.01852E-01	4.43716E-01	**9.03403E-03**	2.77616E-01	2.75896E-01	**9.57635E-04**	8.42424E-01	**1.26065E-02**	**5.20614E-03**
	log2FC	-5.52175E-02	-2.33473E-01	-2.01758E-01	-7.41484E-01	-1.78256E-01	-1.46541E-01	-6.86266E-01	3.17151E-02	-5.08011E-01	-5.39726E-01
Orotic acid*	p	5.27907E-01	9.06380E-01	8.16332E-01	3.51290E-01	4.65064E-01	4.34649E-01	7.42916E-01	9.00827E-01	3.07679E-01	2.96362E-01
	log2FC	1.39321E-01	-2.43931E-02	-5.31715E-02	2.22262E-01	-1.63714E-01	-1.92493E-01	8.29408E-02	-2.87783E-02	2.46655E-01	2.75433E-01
Orotidine*	p	1.60969E-01	8.29783E-01	3.17373E-01	**1.74705E-04**	1.19435E-01	9.65769E-01	**4.22671E-04**	2.68330E-01	**1.25640E-04**	**7.40225E-03**
	log2FC	-4.43813E-01	4.87185E-02	-4.26049E-01	1.41975E+00	4.92532E-01	1.77645E-02	1.86356E+00	-4.74767E-01	1.37103E+00	1.84579E+00
Orthophosphate*	p	1.24685E-01	7.88181E-01	**1.75374E-02**	2.62026E-01	2.75536E-01	7.73462E-01	6.67891E-01	1.11773E-01	4.76672E-01	4.00818E-01
	log2FC	1.75363E-01	2.93243E-02	2.06530E-01	1.19885E-01	-1.46039E-01	3.11664E-02	-5.54785E-02	1.77205E-01	9.05605E-02	-8.66450E-02
Palmitamide	p	8.57868E-01	8.27126E-01	2.73904E-01	**4.56553E-02**	9.53035E-01	2.94857E-01	7.57822E-02	3.32195E-01	1.09342E-01	3.90645E-01
	log2FC	-2.54055E-02	-3.64854E-02	1.46329E-01	2.81768E-01	-1.10799E-02	1.71734E-01	3.07174E-01	1.82814E-01	3.18254E-01	1.35439E-01
Palmitoylcarnitine	p	5.03557E-01	6.57186E-01	6.76327E-01	9.47342E-01	6.44362E-01	3.57973E-01	4.49940E-01	3.03141E-01	4.56088E-01	5.91811E-01
	log2FC	-2.47333E-01	-8.94953E-02	8.96112E-02	1.20483E-02	1.57838E-01	3.36944E-01	2.59381E-01	1.79106E-01	1.01544E-01	-7.75629E-02
Phenylacetylglycine*	p	8.12958E-01	1.71290E-01	**2.91048E-02**	2.82510E-01	8.98684E-02	**1.20664E-02**	1.60912E-01	**2.40926E-02**	5.27307E-01	**5.95655E-03**

	log2FC	-1.48012E-01	9.61570E-01	2.12881E+00	6.94005E-01	1.10958E+00	2.27682E+00	8.42017E-01	1.16724E+00	-2.67565E-01	-1.43480E+00
Phosphoethanolamine*	p	1.61672E-01	**1.39643E-02**	8.60981E-02	1.94297E-01	2.87103E-01	4.07341E-01	9.01707E-01	9.65160E-01	4.02247E-01	4.91198E-01
	log2FC	-2.03096E-01	-3.86815E-01	-3.96548E-01	-2.26973E-01	-1.83719E-01	-1.93452E-01	-2.38774E-02	-9.73346E-03	1.59842E-01	1.69575E-01
Phosphorylcholine*	p	1.98240E-01	7.94884E-01	8.26150E-01	3.23556E-01	7.25608E-02	1.70228E-01	**9.49533E-03**	5.68324E-01	4.04435E-01	1.48633E-01
	log2FC	-3.76308E-01	9.64359E-02	-7.26086E-02	4.08929E-01	4.72744E-01	3.03699E-01	7.85237E-01	-1.69045E-01	3.12493E-01	4.81538E-01
Pipecolic acid	p	2.99524E-01	1.69948E-01	5.71486E-01	5.67129E-01	5.64196E-01	5.62576E-01	**4.84527E-03**	2.94627E-01	**3.24853E-04**	9.54076E-02
	log2FC	2.14442E-01	2.84271E-01	1.22770E-01	-9.98609E-02	6.98297E-02	-9.16720E-02	-3.14303E-01	-1.61502E-01	-3.84132E-01	-2.22630E-01
Ribose 5-phosphate*	p	3.19330E-01	1.23765E-01	1.38386E-01	3.99537E-01	4.18149E-01	4.24808E-01	8.70147E-01	8.38593E-01	3.64986E-01	3.74114E-01
	log2FC	2.56289E-01	4.02805E-01	4.50532E-01	2.22616E-01	1.46516E-01	1.94242E-01	-3.36727E-02	4.77264E-02	-1.80189E-01	-2.27915E-01
Serine*	p	4.60086E-01	**3.27472E-03**	1.04571E-01	1.12032E-01	**8.42588E-03**	2.86546E-01	3.16647E-01	9.38653E-02	6.31874E-02	9.02907E-01
	log2FC	7.26775E-02	3.84621E-01	1.79719E-01	1.67172E-01	3.11944E-01	1.07041E-01	9.44946E-02	-2.04902E-01	-2.17449E-01	-1.25467E-02
sn-glycero-3-phosphocholine*	p	8.25765E-01	3.33875E-01	5.81354E-01	2.70713E-01	3.21463E-01	5.27569E-01	2.83091E-01	6.08191E-01	9.47401E-01	5.95410E-01
	log2FC	-7.63815E-02	4.57257E-01	1.80750E-01	4.17633E-01	5.33638E-01	2.57132E-01	4.94014E-01	-2.76506E-01	-3.96240E-02	2.36882E-01
sn-glycerol 3-phosphate*	p	9.22263E-01	5.09729E-01	6.48280E-01	2.33257E-01	4.25038E-01	5.68949E-01	1.95761E-01	8.76917E-01	4.63948E-01	4.15266E-01
	log2FC	3.38789E-02	-2.23467E-01	-1.70232E-01	-5.10790E-01	-2.57346E-01	-2.04110E-01	-5.44669E-01	5.32353E-02	-2.87324E-01	-3.40559E-01
Spermidine	p	7.53877E-01	5.48059E-01	**1.61875E-02**	1.50944E-01	3.06583E-01	**2.19173E-03**	5.08046E-02	**2.37479E-02**	3.17240E-01	1.69886E-01
	log2FC	-7.84137E-02	1.57127E-01	6.87422E-01	3.94810E-01	2.35541E-01	7.65835E-01	4.73223E-01	5.30294E-01	2.37682E-01	-2.92612E-01
Taurocholic acid*	p	7.19516E-02	3.35173E-01	6.11633E-01	1.65587E-01	**3.49444E-02**	1.02497E-01	**2.67661E-02**	9.22162E-02	7.11954E-02	**3.51835E-02**
	log2FC	-2.20605E+00	1.10284E+00	-4.59737E-01	2.09044E+00	3.30889E+00	1.74631E+00	4.29649E+00	-1.56258E+00	9.87606E-01	2.55018E+00
Taurodeoxycholic acid*	p	1.70514E-01	2.18633E-01	2.54638E-01	**2.00971E-02**	8.23692E-01	4.24125E-01	3.24120E-01	5.63311E-01	2.16206E-01	5.32008E-02
	log2FC	-6.03765E-01	-5.11052E-01	-3.14292E-01	-9.86023E-01	9.27135E-02	2.89473E-01	-3.82258E-01	1.96760E-01	-4.74971E-01	-6.71731E-01
Timnodonic acid	p	7.13019E-01	4.15941E-01	9.74027E-01	5.96228E-01	3.82119E-01	7.24168E-01	5.05254E-01	4.97268E-01	7.22457E-01	6.89101E-01

	log2FC	-1.06008E-01	2.14669E-01	6.98503E-03	1.08030E-01	3.20677E-01	1.12994E-01	2.14039E-01	-2.07684E-01	-1.06638E-01	1.01045E-01
trans-4-Hydroxyproline*	p	8.54734E-01	3.11626E-01	2.83962E-01	**4.25379E-02**	3.87717E-01	3.45476E-01	5.54885E-02	8.46319E-01	2.96442E-01	4.68418E-01
	log2FC	3.96746E-02	2.58232E-01	3.23779E-01	5.99156E-01	2.18558E-01	2.84105E-01	5.59481E-01	6.55468E-02	3.40923E-01	2.75377E-01
Trimethyllysine (N6.N6.N6)	p	5.77633E-01	1.97319E-01	1.96108E-01	1.24056E-01	3.33549E-01	3.31213E-01	1.75287E-01	8.56393E-01	6.63144E-01	9.24491E-01
	log2FC	1.22819E-01	2.75420E-01	3.04316E-01	3.17782E-01	1.52601E-01	1.81497E-01	1.94963E-01	2.88959E-02	4.23618E-02	1.34660E-02
Uracil	p	4.58215E-01	5.14132E-01	**6.27637E-03**	**1.82217E-02**	9.08860E-01	**1.24062E-03**	**1.34703E-02**	**1.54265E-03**	**1.35515E-02**	7.25661E-01
	log2FC	1.37114E-01	1.22335E-01	6.33747E-01	5.71500E-01	-1.47790E-02	4.96633E-01	4.34386E-01	5.11412E-01	4.49165E-01	-6.22467E-02
Uridine monophosphate*	p	2.43850E-01	8.59370E-01	5.77840E-02	3.67267E-01	1.71624E-01	5.37300E-02	9.38123E-01	**1.68576E-02**	3.65297E-01	3.07398E-01
	log2FC	6.06788E-01	8.73174E-02	1.21281E+00	5.66285E-01	-5.19471E-01	6.06025E-01	-4.05026E-02	1.12550E+00	4.78968E-01	-6.46528E-01
Ursodeoxycholic acid*	p	7.84394E-01	1.04591E-01	4.26950E-01	**3.44334E-03**	9.17226E-02	3.45318E-01	**3.18981E-03**	2.16988E-01	**3.10449E-02**	**5.43935E-03**
	log2FC	8.10738E-02	-1.05182E+00	-2.93285E-01	-2.46165E+00	-1.13289E+00	-3.74358E-01	-2.54272E+00	7.58532E-01	-1.40983E+00	-2.16837E+00

Table S6. outlines the characteristics of the constructed unsupervised and supervised models for pairwise comparisons among the studied groups from the untargeted RP-LC-HRMS/MS method. The first column identifies the groups under comparison, while the second column designates the model type, with N denoting the number of samples. R2X(cum) and Q2(cum) apply to both unsupervised and supervised models, whereas R2Y(cum) and CV-ANOVA specifically relate to unsupervised models. Bold indicates the comparisons where supervised models yielded statistically significant results.

Model	Type	N	R2X(cum)	R2Y(cum)	Q2(cum)	CV-ANOVA
G1-G2	PCA-X	20	4.75000E-01		2.30000E-01	
G1-G3	PCA-X	20	6.54000E-01		2.10000E-01	
G1-G4	PCA-X	20	7.29000E-01		3.09000E-01	
G1-G5	PCA-X	20	6.64000E-01		3.19000E-01	
G2-G3	PCA-X	20	7.04000E-01		3.03000E-01	

G2-G4	PCA-X	20	7.05000E-01		3.31000E-01	
G2-G5	PCA-X	20	7.34000E-01		3.45000E-01	
G3-G4	PCA-X	20	6.01000E-01		2.50000E-01	
G3-G5	PCA-X	20	6.01000E-01		3.29000E-01	
G4-G5	PCA-X	20	6.47000E-01		2.61000E-01	
G1-G2	OPLS-DA	20	6.89000E-01	9.60000E-01	4.39000E-01	4.42440E-01
G1-G3	OPLS-DA	20	2.74000E-01	8.44000E-01	2.32000E-01	3.78394E-01
G1-G4	OPLS-DA	20	3.54000E-01	7.73000E-01	1.89000E-01	5.01287E-01
**G1-G5**	OPLS-DA	20	8.23000E-01	1.00000E+00	9.14000E-01	**3.79516E-03**
G2-G3	OPLS-DA	20	3.31000E-01	8.05000E-01	4.43000E-01	5.36885E-02
G2-G4	OPLS-DA	20	4.70000E-01	9.12000E-01	4.16000E-01	2.39809E-01
**G2-G5**	OPLS-DA	20	6.80000E-01	9.62000E-01	7.69000E-01	**4.37929E-03**
G3-G4	OPLS-DA	20	6.15000E-01	9.60000E-01	4.66000E-01	2.57174E-01
**G3-G5**	OPLS-DA	20	4.20000E-01	9.73000E-01	8.17000E-01	**3.61661E-04**
**G4-G5**	OPLS-DA	20	6.20000E-01	9.94000E-01	8.36000E-01	**2.09597E-03**

Table S7. presents p-values and log2 fold changes from pairwise comparisons of the untargeted GC-MS method. Bold indicates p ≤ 0.05, red for p ≤ 0.01, and blue for |Log2 Fold Change| ≥ 1.5.

		G1-G2	G1-G3	G1-G4	G1-G5	G2-G3	G2-G4	G2-G5	G3-G4	G3-G5	G4-G5
2-Palmitoylglycerol	p	3.97452E-01	8.35252E-01	1.48701E-01	2.64199E-01	2.29174E-01	3.07578E-01	7.12570E-01	**4.00674E-03**	**1.75557E-02**	2.57616E-01
	log2FC	2.55346E-01	-7.38310E-03	3.42389E-01	1.74521E-01	-2.62729E-01	3.49772E-01	1.81904E-01	3.49772E-01	1.81904E-01	-1.67868E-01
Allocholic acid	p	7.34181E-02	7.10631E-01	6.64189E-01	9.52888E-01	8.16839E-02	**1.99561E-02**	5.27626E-02	1.69240E-01	6.99037E-01	5.14473E-01
	log2FC	-9.39136E-01	-1.49709E-01	8.40237E-02	-1.29040E-01	7.89427E-01	2.33732E-01	2.06684E-02	2.33732E-01	2.06684E-02	-2.13064E-01
Allose	p	2.66848E-01	**2.18678E-02**	**1.12431E-02**	**4.31819E-04**	1.44284E-01	7.68031E-02	**1.73356E-03**	5.97675E-01	**2.65250E-03**	5.18344E-02
	log2FC	3.09912E-01	7.11160E-01	7.54792E-01	1.27377E+00	4.01248E-01	4.36317E-02	5.62607E-01	4.36317E-02	5.62607E-01	5.18975E-01
Arabitol	p	4.51566E-01	**4.06558E-03**	**5.03227E-04**	**1.75832E-03**	**1.41164E-02**	**1.38272E-03**	**6.42758E-03**	1.00465E-01	6.89167E-01	1.61515E-01
	log2FC	7.35951E-02	3.66407E-01	4.50469E-01	2.84063E-01	2.92812E-01	8.40619E-02	-8.23441E-02	8.40619E-02	-8.23441E-02	-1.66406E-01
Arachidic acid	p	1.00963E-01	2.50548E-01	7.27132E-02	**9.11912E-04**	5.52531E-01	5.44596E-01	**3.01592E-02**	3.08890E-01	**5.45389E-03**	3.69143E-01

	log2FC	2.11180E-01	1.00884E-01	2.12115E-01	3.50931E-01	-1.10296E-01	1.11231E-01	2.50047E-01	1.11231E-01	2.50047E-01	1.38816E-01
Cholestan-3-ol	p	7.21099E-01	8.12469E-01	3.81610E-01	2.44030E-01	2.97297E-01	2.08516E-01	**2.31896E-02**	5.68908E-02	**1.18187E-02**	3.51151E-01
	log2FC	1.40223E-01	-1.98216E-01	3.11239E-01	4.62710E-01	-3.38439E-01	5.09455E-01	6.60926E-01	5.09455E-01	6.60926E-01	1.51471E-01
Ethanolamine	p	2.67675E-01	7.66557E-01	3.96002E-01	5.17481E-02	1.76647E-01	8.56685E-02	**1.72990E-02**	5.03446E-01	**4.28922E-02**	3.40429E-01
	log2FC	-2.96261E-01	8.11394E-03	4.88033E-02	1.96858E-01	3.04375E-01	4.06894E-02	1.88744E-01	4.06894E-02	1.88744E-01	1.48055E-01
Ethyl alpha-d-glucopyranoside	p	3.72106E-01	8.41039E-01	5.33364E-01	**1.51232E-06**	3.45486E-01	2.02711E-01	**6.12198E-04**	7.62654E-01	**4.84376E-04**	**6.49450E-05**
	log2FC	-3.80340E-01	2.39919E-02	4.17605E-02	2.48681E+00	4.04332E-01	1.77686E-02	2.46282E+00	1.77686E-02	2.46282E+00	2.44505E+00
Fructose	p	3.44987E-01	1.19385E-01	6.72096E-01	**2.38076E-02**	2.61421E-01	7.93468E-01	**1.72949E-02**	3.80885E-01	5.50406E-01	1.83264E-01
	log2FC	2.18457E-01	4.65690E-01	1.69622E-02	4.95031E-01	2.47233E-01	-4.48727E-01	2.93413E-02	-4.48727E-01	2.93413E-02	4.78069E-01
Galactaric acid	p	4.42298E-01	**1.45658E-03**	**5.01414E-04**	**3.19086E-04**	**1.83239E-03**	**5.93749E-04**	**2.98254E-04**	3.80032E-01	3.64500E-01	7.41208E-01
	log2FC	1.22536E-01	6.72416E-01	7.14642E-01	6.42181E-01	5.49880E-01	4.22260E-02	-3.02350E-02	4.22260E-02	-3.02350E-02	-7.24611E-02
Galactose	p	4.49248E-01	3.63076E-01	5.14574E-02	**8.83335E-04**	8.05155E-01	1.58293E-01	**2.36407E-03**	2.88744E-01	**1.10315E-02**	1.14255E-01
	log2FC	2.37342E-01	-1.44450E-01	4.82368E-01	-1.47890E-01	-3.81792E-01	6.26818E-01	-3.44063E-03	6.26818E-01	-3.44063E-03	-6.30258E-01
Glucose	p	1.06499E-01	9.87604E-01	8.13726E-01	3.64659E-01	1.40257E-01	2.04038E-01	3.82505E-01	8.10242E-01	4.11078E-01	4.04968E-01
	log2FC	5.51898E-01	-6.05957E-02	-2.61957E-01	1.29546E-01	-6.12493E-01	-2.01362E-01	1.90142E-01	-2.01362E-01	1.90142E-01	3.91503E-01
Gluonic acid, γ-lactone	p	1.13489E-01	5.02398E-02	**3.44560E-02**	**3.79246E-03**	4.86228E-01	3.21991E-01	**1.94875E-02**	6.70156E-01	5.88690E-02	3.48917E-01
	log2FC	3.80112E-01	4.82966E-01	5.06165E-01	6.91385E-01	1.02854E-01	2.31983E-02	2.08419E-01	2.31983E-02	2.08419E-01	1.85221E-01
Glycerol	p	3.74733E-01	**4.20951E-02**	**6.30319E-03**	1.61849E-01	3.02234E-01	5.87825E-02	7.28544E-01	7.82874E-02	2.71403E-01	**2.35156E-02**
	log2FC	1.73018E-01	3.48028E-01	4.73303E-01	9.53191E-02	1.75010E-01	1.25274E-01	-2.52709E-01	1.25274E-01	-2.52709E-01	-3.77984E-01
Glycerol 1-myristate	p	3.29350E-01	9.32744E-01	1.80238E-01	2.15746E-01	2.45849E-01	6.43685E-01	8.34825E-01	1.06565E-01	1.08813E-01	7.50014E-01
	log2FC	8.30552E-02	-5.09864E-02	1.04079E-02	-4.31468E-02	-1.34042E-01	6.13943E-02	7.83960E-03	6.13943E-02	7.83960E-03	-5.35547E-02
Glycerol monostearate	p	7.89568E-01	2.40498E-01	9.29362E-01	7.03693E-01	**3.58942E-02**	4.88526E-01	3.54996E-01	**4.60830E-02**	2.69754E-01	5.97531E-01
	log2FC	-1.25779E-02	-1.16929E-01	-1.54845E-01	-1.91048E-01	-1.04351E-01	-3.79160E-02	-7.41193E-02	-3.79160E-02	-7.41193E-02	-3.62033E-02
Hydroxyoctanoic acid	p	2.02960E-01	**4.14531E-02**	**4.07836E-03**	**7.98094E-03**	6.38465E-01	1.47069E-01	2.30115E-01	**9.06105E-03**	8.12739E-02	5.55046E-01
	log2FC	2.53985E-01	3.02766E-01	4.07368E-01	3.32152E-01	4.87803E-02	1.04602E-01	2.93861E-02	1.04602E-01	2.93861E-02	-7.52163E-02

Lactose	p	3.76766E-01	**1.09409E-03**	**3.07686E-04**	**2.08074E-04**	1.07823E-01	**4.41469E-02**	**3.39088E-02**	3.80306E-01	2.53890E-01	7.97255E-01
	log2FC	3.04247E-01	1.01564E+00	1.25863E+00	1.34375E+00	7.11390E-01	2.42991E-01	3.28109E-01	2.42991E-01	3.28109E-01	8.51179E-02
Mannitol	p	**4.13035E-02**	3.64125E-01	3.44503E-01	1.33102E-01	1.82758E-01	3.91357E-01	3.44026E-01	8.60241E-01	5.47729E-01	7.88355E-01
	log2FC	5.95247E-01	2.03542E-01	1.85054E-01	2.44073E-01	-3.91705E-01	-1.84885E-02	4.05312E-02	-1.84885E-02	4.05312E-02	5.90197E-02
Methyl alpha-D-ribofuranoside	p	5.73920E-01	4.45948E-01	6.63898E-01	**4.18402E-02**	7.98697E-01	9.91756E-01	7.03102E-02	8.67324E-01	1.14698E-01	2.34636E-01
	log2FC	1.39841E-01	1.80350E-01	4.29083E-02	5.12296E-01	4.05092E-02	-1.37442E-01	3.31946E-01	-1.37442E-01	3.31946E-01	4.69388E-01
Methyl aminoacetate	p	8.47625E-01	2.19589E-01	**1.82285E-03**	**4.14766E-03**	3.55804E-01	**5.88021E-03**	**1.39461E-02**	**4.70638E-03**	**1.31759E-02**	3.21376E-01
	log2FC	-2.44776E-03	4.36977E-02	1.32558E-01	6.17468E-02	4.61455E-02	8.88599E-02	1.80491E-02	8.88599E-02	1.80491E-02	-7.08108E-02
Myo-Inositol	p	7.74989E-02	4.52212E-01	1.37480E-01	3.49191E-01	2.03144E-01	**6.20019E-03**	**1.27261E-02**	**3.55515E-02**	7.96765E-02	3.97655E-01
	log2FC	-2.39109E-01	-1.29448E-01	3.59775E-02	-7.94701E-02	1.09660E-01	1.65426E-01	4.99783E-02	1.65426E-01	4.99783E-02	-1.15448E-01
Oleic acid	p	2.82628E-01	2.10671E-01	**2.49446E-02**	7.32661E-02	8.93928E-01	1.00052E-01	3.80267E-01	**8.09798E-03**	1.90453E-01	**3.68104E-02**
	log2FC	4.51022E-01	4.54995E-01	9.36052E-01	5.90816E-01	3.97328E-03	4.81056E-01	1.35821E-01	4.81056E-01	1.35821E-01	-3.45235E-01
Palmitic acid	p	2.00608E-01	**4.09549E-02**	**4.35336E-03**	**8.59925E-03**	6.41271E-01	1.61232E-01	2.48994E-01	**1.04630E-02**	1.00732E-01	5.54419E-01
	log2FC	2.72152E-01	3.25800E-01	4.32610E-01	3.55353E-01	5.36475E-02	1.06810E-01	2.95527E-02	1.06810E-01	2.95527E-02	-7.72572E-02
Propane-1,3-diol	p	6.08759E-01	**2.01343E-03**	4.79959E-01	**1.08258E-03**	**7.08434E-03**	7.33651E-01	**3.35644E-03**	1.04550E-01	5.60624E-01	5.46592E-02
	log2FC	-1.53437E-02	-1.96757E-02	-1.38163E-01	-1.30764E-01	-4.33206E-03	-1.18487E-01	-1.11088E-01	-1.18487E-01	-1.11088E-01	7.39887E-03
Rhamnose	p	6.90577E-02	4.11409E-01	9.31963E-01	4.53086E-01	3.79327E-01	2.15764E-01	1.97999E-01	5.21292E-01	8.41000E-01	5.65464E-01
	log2FC	6.15751E-01	2.52726E-01	-1.86616E-01	6.44432E-02	-3.63025E-01	-4.39342E-01	-1.88282E-01	-4.39342E-01	-1.88282E-01	2.51060E-01
Ribitol	p	9.25144E-01	1.46136E-01	2.29494E-01	**1.47443E-02**	1.86541E-01	2.06166E-01	**1.78820E-02**	**1.70099E-02**	**2.40149E-04**	4.25900E-01
	log2FC	-5.57275E-02	-3.18782E-01	1.05146E-01	2.28260E-01	-2.63055E-01	4.23928E-01	5.47043E-01	4.23928E-01	5.47043E-01	1.23115E-01
Ribonic acid	p	2.83683E-01	2.88062E-01	8.09912E-02	1.04850E-01	9.49998E-01	1.44097E-01	2.46066E-01	1.46869E-01	2.58541E-01	7.25654E-01
	log2FC	3.81178E-01	3.51576E-01	5.73375E-01	4.82634E-01	-2.96016E-02	2.21798E-01	1.31058E-01	2.21798E-01	1.31058E-01	-9.07402E-02
Ribonolactone	p	4.46917E-01	4.01999E-01	**4.72744E-02**	**8.26897E-04**	8.61437E-01	1.36599E-01	**1.36541E-03**	2.40329E-01	**6.75122E-03**	6.83739E-02
	log2FC	1.12026E-01	1.16706E-01	2.77136E-01	6.06425E-01	4.68055E-03	1.60429E-01	4.89719E-01	1.60429E-01	4.89719E-01	3.29290E-01
Ribulose	p	**2.28215E-02**	8.14210E-02	1.09954E-01	**3.47854E-02**	1.09686E-01	1.58484E-01	3.91665E-01	7.50801E-01	3.18269E-01	3.21360E-01

	log2FC	1.32369E+00	8.78027E-01	6.85150E-01	9.72331E-01	-4.45665E-01	-1.92878E-01	9.43033E-02	-1.92878E-01	9.43033E-02	2.87181E-01
Scyllo-Inositol	p	4.42678E-01	4.12097E-01	7.76383E-01	3.12342E-01	8.65177E-01	8.67434E-01	7.13621E-01	8.05691E-01	8.65988E-01	7.16430E-01
	log2FC	9.65675E-02	9.70047E-02	-5.70132E-02	1.72548E-02	4.37156E-04	-1.54018E-01	-7.97498E-02	-1.54018E-01	-7.97498E-02	7.42681E-02
Sorbose	p	3.05646E-01	7.14257E-02	2.20292E-01	**4.05186E-03**	2.17652E-01	6.73917E-01	**3.42181E-03**	5.44032E-01	8.78096E-02	6.03449E-02
	log2FC	2.40203E-01	4.99641E-01	2.34316E-01	7.43571E-01	2.59437E-01	-2.65324E-01	2.43930E-01	-2.65324E-01	2.43930E-01	5.09254E-01
Stearic acid	p	1.64998E-01	2.97240E-01	7.34200E-02	5.52503E-02	3.46315E-01	1.52250E-01	**4.99640E-02**	**3.82420E-02**	**1.05731E-02**	6.88476E-01
	log2FC	1.75954E-01	1.36899E-01	2.38895E-01	2.56626E-01	-3.90551E-02	1.01996E-01	1.19727E-01	1.01996E-01	1.19727E-01	1.77309E-02
Threonine	p	6.28653E-01	**5.16889E-03**	**4.94986E-03**	**9.63527E-03**	5.88312E-02	5.55228E-02	1.01675E-01	9.50876E-01	5.22538E-01	5.67271E-01
	log2FC	4.55004E-02	3.47972E-01	2.60760E-01	1.64328E-01	3.02472E-01	-8.72121E-02	-1.83645E-01	-8.72121E-02	-1.83645E-01	-9.64326E-02
Xylose	p	2.41832E-01	5.75561E-01	4.34957E-01	**7.41416E-03**	1.83017E-01	1.67129E-01	3.94568E-01	7.36267E-01	**3.93712E-02**	6.51003E-02
	log2FC	4.61655E-01	-2.38107E-01	-4.32403E-01	8.29304E-01	-6.99762E-01	-1.94296E-01	1.06741E+00	-1.94296E-01	1.06741E+00	1.26171E+00

Table S8. outlines the characteristics of the constructed unsupervised and supervised models for pairwise comparisons among the studied groups from the untargeted GC-MS method. The first column identifies the groups under comparison, while the second column designates the model type, with N denoting the number of samples. R2X(cum) and Q2(cum) apply to both unsupervised and supervised models, whereas R2Y(cum) and CV-ANOVA specifically relate to unsupervised models. Bold indicates the comparisons where supervised models yielded statistically significant results.

Model	Type	N	R2X(cum)	R2Y(cum)	Q2(cum)	CV_ANOVA
G1-G2	PCA-X	20	6.78000E-01		3.84000E-01	
G1-G3	PCA-X	20	7.26000E-01		3.31000E-01	
G1-G4	PCA-X	20	7.91000E-01		4.33000E-01	
G1-G5	PCA-X	20	7.10000E-01		3.96000E-01	
G2-G3	PCA-X	20	6.98000E-01		4.40000E-01	
G2-G4	PCA-X	20	7.50000E-01		4.77000E-01	
G2-G5	PCA-X	20	7.45000E-01		5.08000E-01	
G3-G4	PCA-X	20	7.13000E-01		5.20000E-01	
G3-G5	PCA-X	20	6.51000E-01		3.80000E-01	

G4-G5	PCA-X	20	7.52000E-01		4.47000E-01	
G1-G2	OPLS-DA	20	4.71000E-01	5.34000E-01	-3.37000E-02	1.00000E+00
G1-G3	OPLS-DA	20	4.60000E-01	6.52000E-01	2.78000E-01	2.68591E-01
**G1-G4**	OPLS-DA	20	5.48000E-01	7.32000E-01	5.24000E-01	**1.87782E-02**
**G1-G5**	OPLS-DA	20	7.39000E-01	9.82000E-01	8.56000E-01	**3.14664E-04**
G2-G3	OPLS-DA	20	4.95000E-01	5.73000E-01	1.28000E-01	7.03172E-01
**G2-G4**	OPLS-DA	20	7.84000E-01	8.76000E-01	6.96000E-01	**1.86633E-02**
**G2-G5**	OPLS-DA	20	7.83000E-01	9.37000E-01	7.44000E-01	**7.56497E-03**
G3-G4	OPLS-DA	20	7.28000E-01	9.51000E-01	5.85000E-01	8.60127E-02
**G3-G5**	OPLS-DA	20	6.65000E-01	9.69000E-01	8.22000E-01	**1.05170E-03**
**G4-G5**	OPLS-DA	20	5.94000E-01	9.18000E-01	6.93000E-01	**7.92885E-03**

Table S9. compiles the statistically significant compounds identified via univariate statistics among the 286 compounds analyzed, focusing on comparisons between the control group and three distinct treatments (G1: metronidazole; G2: probiotics; G3: FMT; G4: untreated controls).

G1-G5	G2-G5	G3-G5
3-methyl-2-oxovaleric acid[1]	3,4-dihydroxyhydrocinnmaic acid[1]	3-hydroxybutyric acid[1]
2-aminoadipate[3]	2-aminoadipate[3]	Allose[4]
3-hydroxybutyric acid[1]	3-hydroxybutyric acid[1]	2-aminoadipate[3]
4-aminobutyric acid[1]	4-aminobutyric acid[1]	2-palmitoylglycerol[4]
4-hydroxybenzoic acid[1]	4-hydroxybenzoic acid[1]	4-aminobutyric acid[1]
4-hydroxyphenylacetic acid[1]	5-hydroxyindole-3-acetic acid[1]	4-trimethylammoniobutanoate3
4-trimethylammoniobutanoate[3]	adipic acid[1]	anthranilic acid[2]
adenine[2]	allose[4]	arachidic acid[4]
adenosine[2]	arabitol[4]	arginine[3]
adipic acid[1]	arachidic acid[4]	asparagine[3]
allose[4]	carnitine[3]	aspartic acid[3]
anthranilic acid[2]	cholestan-3-ol[4]	azelaic acid[1]
arabitol[4]	cholesterol[3]	cholestan-3-ol[4]
arachidic acid[4]	citric acid[1]	citric acid[1]

asparagine[3]	cytidine[3]	citrulline[3]
benzoic acid[1]	deoxycholic acid[3]	cyclic adenosine monophosphate[3]
butyrylcarnitine[3]	dimethylglycine[3]	cytidine[3]
carnitine[3]	docosenamide[3]	dimethylglycine[3]
cholesterol[3]	ethanolamine[4]	ethanolamine[4]
citric acid[1]	ethyl alpha-d-glucopyranoside[4]	ethyl linoleate[3]
citrulline[3]	fructose[4]	galactose[4]
cytidine[3]	fumaric acid[1]	gluconic acid[3]
deoxycholic acid[3]	galactaric acid[4]	glutamic acid[3]
dimethylglycine[3]	galactose[4]	glutamine[3]
ethyl alpha-d-glucopyranoside[4]	glucose 6-phosphate[3]	glutathione disulfide[3]
ethylmalonic acid[1]	gluonic acid, γ-lactone[4]	glyceric acid[1]
fructose[4]	glutamic acid[3]	LPC 20:4[3]
fumaric acid[1]	glutamine[3]	LPC 22:4[3]
galactaric acid[4]	glutathione disulfide[3]	LPE 17:0[3]
galactose[4]	glyceric acid[1]	LPE 20:4[3]
galactose[4]	glycine[3]	LPE 22:4[3]
galacturonic acid[3]	histidine[3]	LPE 22:6[3]
gluconic acid[3]	homoserine[3]	LPG 18:1[3]
glucose 6-phosphate / fructose 6-phosphate[3]	lactic acid[1]	LPG 20:4[3]
gluonic acid, γ-lactone[4]	lactose[4]	LPG 22:6[3]
glutamic acid[3]	LPC 15:0[3]	LPI 20:4[3]
glutamine[3]	LPC 17:0[3]	LPI 22:6[3]
glutathione disulfide[3]	LPE 16:1[3]	lysine[3]
glyceric acid[1]	LPE 17:0[3]	methyl aminoacetate[4]
glycine[3]	LPE 18:1[3]	methylmalonic acid[1]
glycolic acid[1]	LPE 18:2[3]	monoisoamylamine[2]
histamine[2]	LPE 20:1[3]	N-acetylneuraminic acid[3]
histidine[3]	LPE 20:4 [3]	N-linoleoyl taurine[3]
homoserine / threonine[3]	LPE 22:4[3]	ornithine[3]
hydroxyoctanoic acid[4]	LPE 22:6[3]	orotidine[3]

hypotaurine[2]	LPG 22:6[3]	pipecolic acid[3]
isoleucine[2]	lysine[3]	pyroglutamic acid[1]
lactose[4]	maleic acid[3]	ribitol[4]
leucine[2]	malic acid[1]	ribonolactone[4]
LPC 20:5 [3]	malonic acid[1]	stearic acid[4]
LPC 22:4 [3]	methyl aminoacetate[4]	succinic acid[3]
LPE 16:1 [3]	methylamine[2]	succinic acid[1]
LPE 18:1 [3]	methylmalonic acid[1]	uracil[3]
LPE 20:1 [3]	MG 20:4 [3]	uridine[2]
LPE 20:4 [3]	myo-Inositol[4]	ursodeoxycholic acid[3]
LPE 22:4[3]	N2-acetyllysine[3]	vallylmandelic acid[1]
LPE 22:6[3]	N-linoleoyl taurine[3]	xylose[4]
LPG 20:4[3]	ornithine[3]	
LPG 22:6[3]	orotidine[3]	
LPI 22:6[3]	phosphorylcholine[3]	
maleic acid[3]	pipecolic acid[3]	
malic acid[1]	propane-1,3-diol[4]	
malonic acid[1]	putrescine[2]	
methyl alpha-d-ribofuranoside[4]	pyroglutamic acid[1]	
methyl aminoacetate[4]	ribitol[4]	
methyl arachidonate (20:4)[3]	ribonolactone[4]	
methyl docosahexaenoate (22:6)[3]	sorbose[4]	
methylmalonic acid[1]	stearic acid[4]	
MG 14:0[3]	taurocholic acid[3]	
MG 16:1[3]	trimethylamine-n-oxide[2]	
MG 18:1 [3]	uracil[3]	
MG 18:2[3]	uridine[2]	
MG 20:4[3]	ursodeoxycholic acid[3]	
monoisoamylamine[2]	vallylmandelic acid [1]	
N2-acetyllysine[3]		
N-acetylneuraminic acid[3]		

N-arachidonoyl taurine[3]
n-linoleoyl taurine[3]
ornithine[3]
orotidine[3]
palmitamide[3]
palmitic acid[4]
phenylalanine[2]
propane-1,3-diol[4]
ribitol[4]
ribonolactone[4]
ribulose[4]
sorbose[4]
succinic acid[3]
taurodeoxycholic acid[3]
threonine[4]
trans-4-hydroxyproline[3]
uracil[3]
ursodeoxycholic acid[3]
vallylmandelic acid[1]
xylose[4]

[1] GC-MS/MS

[2] HILIC-MS/MS

[3] RP-LC-HRMS/MS

[4] GC-MS

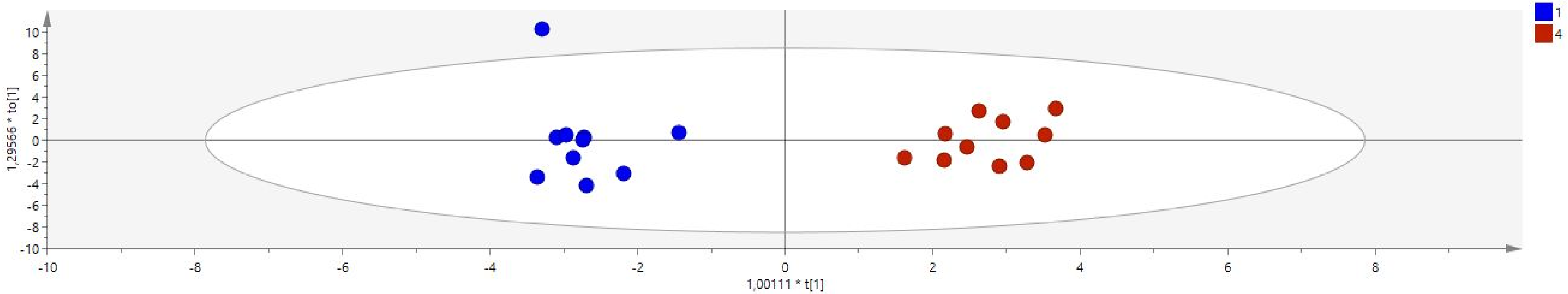

a.

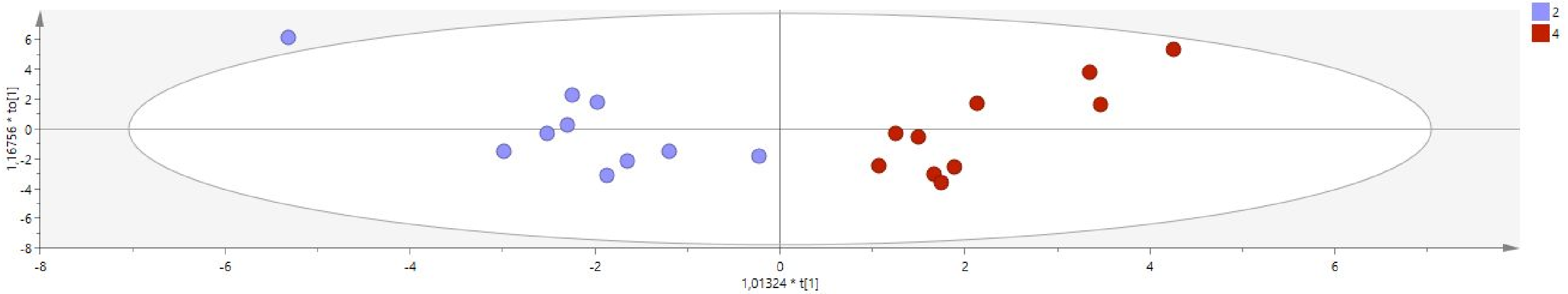

b.

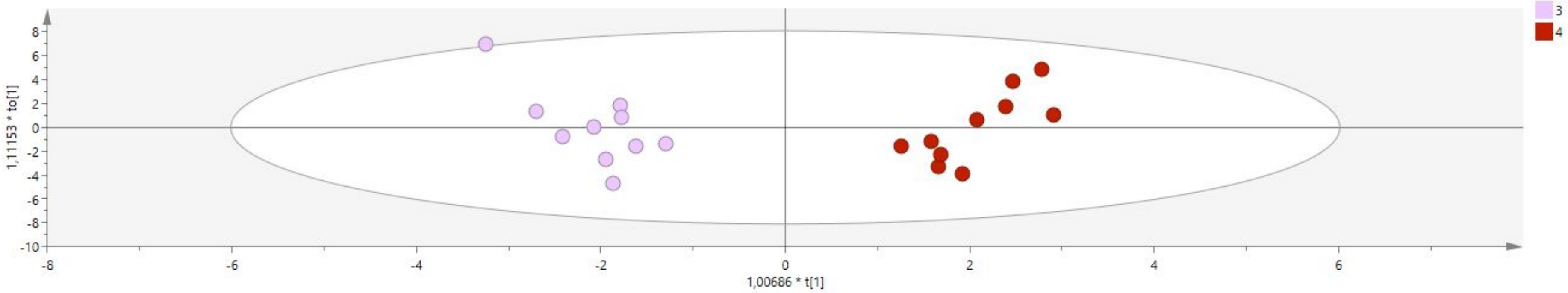

c.

Figure S1. *OPLS-DA score plots from the targeted* <u>*GC-MS/MS*</u> *method illustrate the model constructed to discern between G4 (C. difficile untreated group, represented in red) and three treatments: a. metronidazole (depicted in blue), b. probiotics (light blue), and c. FMT (purple). In each of the three discriminant models, the clear clustering of samples along the first component (t[1]) distinctly highlights alterations in the metabolome post-treatment for C. difficile infection. These results underscore the effectiveness of the treatments in inducing discernible metabolic shifts. Table X distinctly showcases statistically significant and strong group differentiation, as underscored by the CV-ANOVA results in their respective models.*

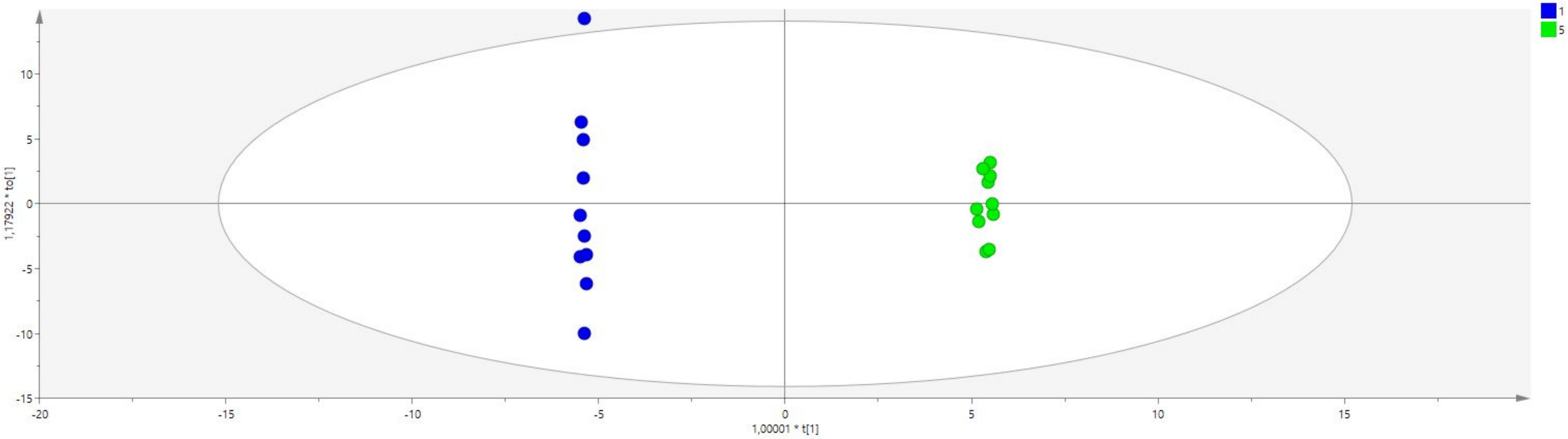
1
5
10
5
0
-5
-10
-15
1,17922 * to[1]
-20
-15
-10
-5
0
5
10
15
1,00001 * t[1]

a.

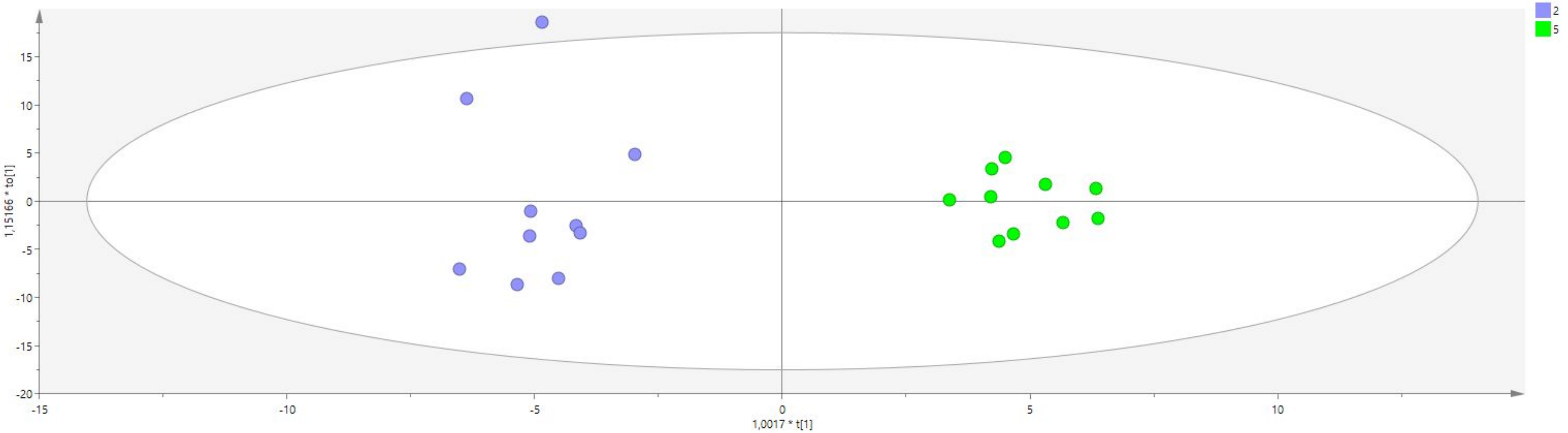
2
5
15
10
5
0
-5
-10
-15
-20
1,15166 * to[1]
-15
-10
-5
0
5
10
1,0017 * t[1]

b.

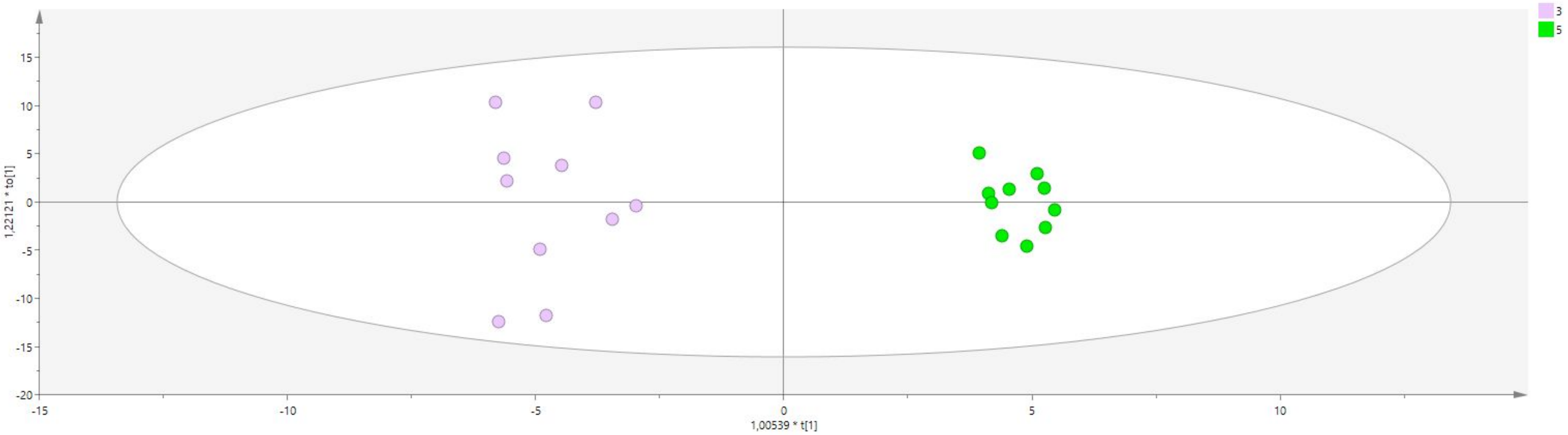

c.

Figure S2. *OPLS-DA score plots from the untargeted RP-LC-HRMS/MS analysis illustrate the constructed models between the C. difficile-infected mice group treated with three different treatments: a. metronidazole (depicted in blue), b. probiotics (light blue), and c. FMT (purple), compared to the control group. The clear clusters of the groups in every treatment, along with the low CV-ANOVA value characteristic of a strong model, indicate that none of the treatments could restore the metabolome to the baseline level. These results suggest that the changes made to the metabolome after antibiotic treatment, followed by C. difficile infection and subsequent treatment, led to irreversible changes in the cecal metabolome. Table X presents in detail the characteristics of the constructed models.*
